# Recovery of complete genomes and non-chromosomal replicons from activated sludge enrichment microbial communities with long read metagenome sequencing

**DOI:** 10.1038/s41522-021-00196-6

**Published:** 2021-03-16

**Authors:** Krithika Arumugam, Irina Bessarab, Mindia A. S. Haryono, Xianghui Liu, Rogelio E. Zuniga–Montanez, Samarpita Roy, Guanglei Qiu, Daniela I. Drautz–Moses, Ying Yu Law, Stefan Wuertz, Federico M. Lauro, Daniel H. Huson, Rohan B. H. Williams

**Affiliations:** 1 Singapore Centre for Environmental Life Sciences Engineering, Nanyang Technological University, Singapore, Singapore; 2 Singapore Centre for Environmental Life Sciences Engineering, National University of Singapore, Singapore, Singapore; 3 Department of Civil and Environmental Engineering, One Shields Avenue, University of California, Davis, CA USA; 4 School of Civil and Environmental Engineering, Nanyang Technological University, Singapore, Singapore; 5 Asian School of the Environment, Nanyang Technological University, Singapore, Singapore; 6 Institute for Bioinformatics and Medical Informatics, University of Tübingen, Tübingen, Germany; 7 Life Sciences Institute, National University of Singapore, Singapore, Singapore; 8 Present Address: School of Environment and Energy, South China University of Technology, Guangzhou, China

**Keywords:** Metagenomics, Next-generation sequencing, Water microbiology

## Abstract

New long read sequencing technologies offer huge potential for effective recovery of complete, closed genomes from complex microbial communities. Using long read data (ONT MinION) obtained from an ensemble of activated sludge enrichment bioreactors we recover 22 closed or complete genomes of community members, including several species known to play key functional roles in wastewater bioprocesses, specifically microbes known to exhibit the polyphosphate- and glycogen-accumulating organism phenotypes (namely *Candidatus* Accumulibacter and *Dechloromonas*, and *Micropruina*, *Defluviicoccus* and *Candidatus* Contendobacter, respectively), and filamentous bacteria (*Thiothrix*) associated with the formation and stability of activated sludge flocs. Additionally we demonstrate the recovery of close to 100 circularised plasmids, phages and small microbial genomes from these microbial communities using long read assembled sequence. We describe methods for validating long read assembled genomes using their counterpart short read metagenome-assembled genomes, and assess the influence of different correction procedures on genome quality and predicted gene quality. Our findings establish the feasibility of performing long read metagenome-assembled genome recovery for both chromosomal and non-chromosomal replicons, and demonstrate the value of parallel sampling of moderately complex enrichment communities to obtaining high quality reference genomes of key functional species relevant for wastewater bioprocesses.

## Introduction

The development of long read sequencing technologies, such as the Oxford Nanopore Technology MinION and Pacific Biosciences SMRT are presenting new opportunities for the effective recovery of complete, closed genomes^1,2^. While these new approaches have been mostly applied to single species isolates^3,4^, the ability of this new methodology to recover genomes of member taxa from complex microbial communities (microbiome) data is now actively being explored.

After long read sequencing technologies first became available, several studies pioneered the collection of long read data, or combined long and short read data, on complex microbial communities, for example from moderately to highly enriched bioreactor communities^5,6^, co–culture enrichments^7^, marine holobionts^8^ or from full-scale anaerobic digester communities^9^, as well as several datasets which provided benchmarking data from long and short read sequencing of mock communities^10–12^. New long read analysis methods^13,14^ and binning algorithms designed for long read metagenome data^15^ have also appeared, anticipating the future expansion of metagenome data generated from these new instruments. More recent studies^12,16–23^ have collectively established that full length (or near-full length genomes) can be recovered from long read sequencing of complex communities, which sets the stage for further development of genome-resolved long read metagenomics.

In this paper we extend our previous work^16^ on recovering metagenome-assembled genomes from long read data obtained from enrichment (continuous culture) reactors inoculated with activated sludge microbial communities. Enrichment reactor communities^24,25^ offer a moderate level of complexity compared to the inoculum communities^26^ and so are realistic, yet tractable, systems to use for developing approaches for recovery and validation of MAG analysis^27^. We report results and methodology of long read sequencing from multiple sets of enrichment reactor communities. We have obtained short read metagenome data (Illumina) from either the same DNA aliquots as used for long read sequencing, or the same biomass. Specifically we (1) describe new methods for validating long read assembled genomes using their counterpart short read metagenome-assembled genomes; (2) assess the influence of different correction procedures on genome quality and predicted gene quality; (3) contribute 22 new closed or complete genomes of community members, including several species known to play key functional roles in wastewater bioprocesses and (4) compare and contrast the use of long read metagenome assembly to the results obtained from hybrid metagenome assembly workflows.

## Results

### Overview, long read sequencing and genome recovery

We sampled biomass from four different activated sludge enrichment (continuous culture) bioreactors, each designed to enrich the abundance of different microbes exhibiting the *polyphosphate-accumulating organism* (PAO) phenotype. Such microbes underpin the feasibility of operating a nutrient removal bioprocess called *enhanced biological phosphorus removal*, which can remove excess phosphorus from soluble wastewater streams^28^. These laboratory-scale reactors are inoculated with microbial communities sampled from activated sludge tanks in full-scale operational wastewater treatment plants (operated by the Public Utilities Board, Republic of Singapore). Activated sludge communities are highly complex, with about an order of magnitude greater ecological richness than that observed in the human gut microbiome^26^. The use of medium- to long-term continuous culture methods, which apply specific physico-chemical conditions to the inoculum, results in the simplification of community complexity and an increase in the relative abundance of species of functional relevance. When viewed from the objectives of the present study, these long-term enrichment experiments hold a complexity that is less than the source community, while maintaining a realistic level of complexity, and therefore they are ideal systems for developing new methods for member genome recovery. As described in the Materials and Methods, we obtained both long and short read sequence from whole-community genomic DNA and performed metagenome assembly separately on both types of data as described below.

Long read sequencing depth improved from the beginning of the study period, reflecting rapid improvements in experimental protocols and flowcell technology (Supplementary Table 1), with the total amount of sequence generated ranging from around 1Gbp/run to just under 12Gbp/run (Supplementary Table 1). These data were assembled using three workflows, namely Canu^29^, Unicycler^30^ and Flye^31^ and we refer to these sequences as *long read assembled contig* (LRAC). We considered LRAC sequence that was at least 1Mbp in length as potential whole-chromosome sequence and these are described as *LR-chr sequence* hereon. The Canu assembly workflow generated a greater number of LR-chr (*n* = 90) on these data than did either Unicycler (*n* = 44) or Flye (*n* = 60) (Supplementary Table 2). As Canu generated a substantially larger number of LR-chr sequences, we subsequently focused attention on the results obtained with this workflow (see Supplementary Table 3 for comparative summary of LR-chr sequences from each workflow).

We corrected frame-shift errors in LR-chr sequence using MEGAN-LR^13,16^ and applied the CheckM workflow^32^ to estimate genome quality. We observed a total of 24 LR-chr sequences generated from Canu that could be considered plausible candidates for being whole-chromosomal sequences, due to their having completeness >90% and contamination <5%, as estimated by CheckM and we refer to them as *putative genomes* for convenience. A further 13 LR-chr sequences from Canu were classifiable as medium quality (SCG-estimated completeness ≥50% and contamination <10%). We de-replicated the entire set of 24 putative genomes using the dRep workflow^33^ with a relatedness threshold of ANImf > 99 (Supplementary Fig. 1), obtaining a reduced set of 22 putative genomes. The two redundant putative genomes were obtained from the PAO3A and PAO3B datasets, consistent with the fact that they are the same community sampled at different times.

We then studied each of these 22 de-replicated putative genomes in more detail to establish whether they were, or were not, likely to represent whole chromosomes. Using annotations from the Prokka workflow^34^, all 22 putative genomes met the complete MIMAG criteria^35^ for being classified as high quality metagenome-assembled genomes, including a minimum number of tRNA encoding genes, and the presence of each of the genes encoding 5S, 16S and 23S SSU-rRNAs (Table 1).

**Table 1:** Summary statistics for 22 putative genomes recovered in this study.

Genome identifier	Length (bp)	#CDS^a^	#rRNA^a^/#tRNA	Completeness^b^/contamination	Taxonomic annotation^c^	Genbank
PAO1-tig00000001	5,190,177	5116	2/53	95.28/1.11	s__Accumulibacter sp000584975	CP058708.1
PAO1-tig00000003	4,268,816	5123	1/36	92.82/0.25	g__OLB11	CP058707.1
PAO1–tig00000117	2,656,706	3153	1/38	97.53/0.15	f__UBA6002	CP058706.1
PAO1–tig00026549	4,352,448	4225	1/46	94.64/0.50	f__2–12–FULL–67–15	CP058705.1
PAO1-tig00026557	3,138,394	3619	2/44	94.19/1.58	f__Parachlamydiaceae	CP058704.1
PAO1–tig00026560	4,262,704	4373	2/37	93.99/0.55	g__ELB16–189	CP058703.1
PAO1–tig00198536	3,913,768	3521	2/38	92.57/1.98	s__OLB8 sp001567405	CP058702.1
PAO2-tig00000001^d^	5,027,886	4558	2/46	93.85/0.00	g__Accumulibacter	CP054595.1
PAO2–tig00000013	3,452,123	3382	2/46	94.49/0.18	g__Dechloromonas	CP054864.1
PAO3A–tig00000003	3,666,458	3454	1/53	94.56/0.28	g__Micropruina	CP059261.1
PAO3A–tig00000011^d^	3,813,965	3532	2/53	94.31/2.17	g__Contendobacter	Pending
PAO3A–tig00000024^d^	2,740,818	2753	1/44	96.92/0.00	s__Brevundimonas sp002426005	CP059260.1
PAO3A–tig00000209	3,302,829	3190	1/47	95.38/0.00	f__Nocardioidaceae	CP059259.1
PAO3A–tig00018026	4,685,957	4742	1/48	93.47/1.09	c__Anaerolineae	CP059258.1
PAO3A–tig00139797^d^	3,548,924	3508	2/47	99.04/0.48	s__Thiobacillus sp00189930	CP059257.1
PAO3B–tig00000024^d^	3,282,734	2937	2/51	98.10/0.23	f__Burkholderiaceae	CP059256.1
PAO3B–tig00000027^d^	3,375,962	3179	1/53	93.16/0.42	g__Rhodoblastus	CP059255.1
PAO4–tig00000001^d^	3,950,501	3652	2/58	98.64/2.07	g__Pseudoxanthomonas_A	CP059266.1
PAO4–tig00000030^d^	4,541,730	4623	2/47	97.10/2.30	g__Thiothrix	CP059265.1
PAO4–tig00000046^d^	3,961,963	3725	2/51	96.30/0.47	g__Dechloromonas	CP059264.1
PAO4–tig00000079^d^	2,921,657	3197	9/59	94.79/0.00	s__Exiguobacterium profundum	Pending
PAO4–tig00000228	4,261,978	4404	2/50	94.81/0.00	g__UBA1943	Pending

^a^As predicted using Prokka (see Methods). The rRNA count refers to the occurrence of complete rRNA operons (5S, 16S, 23S).

^b^Genome quality estimates from CheckM (see Methods).

^c^Taxonomic assignments from GTDB-Tk (see Methods).

^d^Sequence classified as *circular* by Canu.

Estimated SCG completeness was on average 95.27% (range 92.57–99.04%) and mean contamination was 0.72% (range 0.00–2.30%). Ten of the 22 sequences were classified as circular by Canu (Table 1). Coverage profiles generated using both long and short read data within a given community showed uniform coverage, with no substantive gaps observed (see Panel C of Fig. 1 and Supplementary Figs 2–31). On average 37.6% of long reads were utilised to produce putative genomes (range: 23.3–55.4; dataset specific see Supplementary Table 4). At the individual genome level as few as 1% of reads in a dataset could generate a complete chromosomal sequence Supplementary Table 4.

**Fig. 1:**
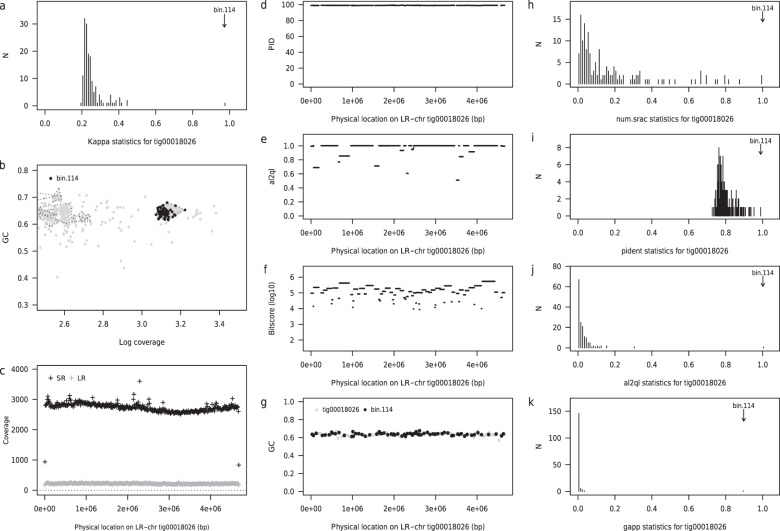
Summary of concordance statistic analysis for an LR-chr (tig00018026) from the PAO3A reactor community (annotated to class *Anaerolineae* showing close relationship to a short read metagenome-assembled genome from the same reactor community (bin 114). **a** Distribution of *κ*--scores for tig00018026 against 242 bins recovered from the corresponding short read assembly. Bin 114 has the highest *κ* at 0.97; **b** coverage--GC plot for the short read assembly, with bin 114 highlighted (closed black circles and dark grey convex hull; other bins highlighted by light grey convex hulls); **c** short read (*SR*, black crosses) and long read *(LR*, grey crosses) coverage profiles across tig00018026. **d**–**f** BLASTN statistics for alignments of short read contigs (bin 114) against tig00018026. Horizontal segments show alignment position on LR--chr and height of segment is value of corresponding statistic (*y*--axis) namely percent identity (PID) (**d**), the ratio of alignment length to query length (al2ql) (**e**) and *log*_10_--bit-score (**f**). **g** GC content as a function of position on tig00018026 (grey closed circles, computed in adjacent windows of length 46700 bp) and for aligned short read contigs (black closed circles); **h–k** distribution of four component statistics of *κ* (see Methods), with the position of the top scoring short read bin highlighted. **h** proportion of short read contigs in bin aligned to LR--chr (*p*_*srac*_); **i** mean percent identity ($\widehat{{\mathit{pid}}}$). **j** mean ratio of alignment length to query length $\widehat{{\mathit{al2ql}}}$ and **k** proportion of the long read contig that is covered by an alignment (*p*_*aln*_).

To gain further insight into the quality and completeness of detected genomes, we used the *concordance statistic* (*κ*), previously developed by us^16^ to identify metagenome-assembled genomes obtained from short read sequence data (see Methods: Analysis of short read sequence data and Supplementary Table 5) that were cognate to a long read assembled genome. The *κ*–statistic is computed for all combinations of short read MAGs and LR-chr sequences. An observed value of *κ* close to unity will imply that the LR-chr sequence is tiled by the contigs from the short read MAG, and the latter can be considered a likely candidate for being the cognate genome. For 21 of the 22 genomes in Table 1 the maximum observed *κ* values were high (mean: 0.95 range: 0.83–1.00) (Supplementary Data 1). The exception was a genome recovered from PAO4 (PAO4–tig00000079), annotated at species level to *Exiguobacterium profundum*, which held a *κ* value of 0.3 and from which there appeared to be no corresponding complete short read MAG (Supplementary Fig. 30). If we only considered near-full length alignments (*al*2*ql* > 0.95), the mean value of *κ* reduced by around 0.06 units (mean 0.89, range: 0.72–0.99). In Fig. 1 we provide a comprehensive visualisation of the concordance statistic analysis for the case of the PAO3A–tig00018026 genome against its cognate short read MAG (bin 114). Related plots for the remaining 21 genomes are provided in Supplementary Figs 2–31).

On average, for a given LR-chr sequence, *κ*–statistics were generated from around two thirds of available short read MAGs, but in most cases the magnitude of the *κ*–statistic itself was low. Of the four component statistics, $\widehat{{\mathit{pid}}}$ and *p*_*srac*_ showed consistently higher values in the bulk of associations than either $\widehat{{\mathit{al2ql}}}$ or *p*_*aln*_, with the latter two measures providing greater visual discrimination between the short read MAG holding the maximum *κ* value and the bulk distribution of (lower) *κ* scores. As expected, cognate short read MAGs were generally drawn from among the most abundant members of a given reactor community. Contigs from short read bins with related taxonomy usually scored highly on one or more component scores (data not shown), but in combination, only one short read MAG generated a high value *κ* score with component statistics that supported it being the cognate. In several cases, we observe two short read MAGs that tile adjacent black regions of a single LR-chr sequence, which is most likely due to the underlying genome being split by the MetaBAT2 binning algorithm into two or more component sub–MAGs (bin–splitting; see examples in Supplementary Figs 2–3, 5–6, 10–11, 21–23).

### Identification of probable mis-assemblies among LR-chr sequences

Among the entire set of 90 LR-chr sequences we identified several examples of LR-chr that are clearly mis-assemblies. In the PAO3A data, we observed one contig (tig00000001; assembled by Canu) that appeared to be comprised of two separate complete genomes joined together (see Supplementary Figs 32–35 for further dissection). In this case, the proximal two thirds of the LR-chr arises from one genome, while distal third from another, as evidenced by different GC proportions and divergent short bin associations, respectively. In the case of the PAO4 data we observed several LR-chr that were classified by CheckM to have completeness over 90% but which demonstrated substantial degrees of contamination (namely tig00017984, tig00017990 and tig00017987 from Canu, which show CheckM estimated contamination of 50%, 45% and 79%, respectively), most likely as the result of reads from closely related strains being combined.

We also manually checked two genomes (PAO1-tig00000003 and PAO1-tig00026557) whose coverage profiles (see Supplementary Figs 5–6 and Supplementary Fig. 9, respectively) suggested potential for mis-assembly. In the case of PAO1-tig00026557, a region of low coverage in approximately the first 300 kbp of sequence suggested the concatenation of non-cognate sequence and this was confirmed to be the case by examining read alignments. In the case of PAO1-tig00000003, the region between ~3.5 and 4 Mbp showed a clear reduction in GC content of around 0.5 units from the rest of the genome. Each region mapped to a different short read bin, suggesting the possibility of mis-assembly. However, examination of read alignments of the reduced GC region and flanking genome suggested no features consistent with mis-assembly, so we can conclude the cognate genome has been appropriately reconstructed.

### Effect of sequence error correction on coding sequence and genome quality

**Fig. 2:**
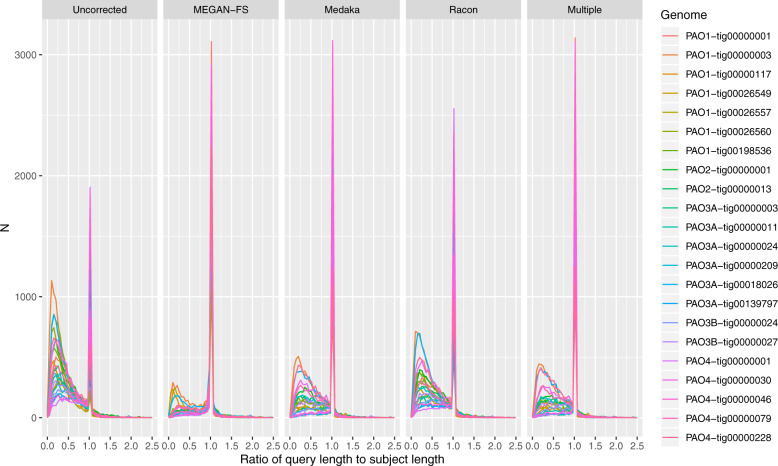
Density estimates for the length ratio statistics, computed from the length of predicted genes in long read assemblies (query) and length of their best hit counterparts in cognate short assemblies (subjects), and categorised by type of sequence correction employed (from left to right, raw assembled sequence [uncorrected], frame-shift correction using MEGAN-LR, sequence correction using Medaka, sequence correction using Racon and application of the multiple procedure approach. Results from individual recovered genomes are highlighted by colour, and *x*-axis truncated at 2.5 units. A version with a log-scale on the vertical axis is provided in Supplementary Fig. 36.

Although the recovered genomes are consistent with being bona fide whole-bacterial chromosomal sequence, the high error rate present in current nanopore-based sequencing implies these constructs may not meet current expectations of reference genome quality. Examining the length ratio histograms of the predicted genes from long read assembled genomes, against their best hit counterparts from the cognate short read assemblies, we observed that application of any of the three sequence correction procedures provided some degree of improvement compared to the case of raw sequence, with an increased frequency of the length ratio being located around a value of unity (Fig. 2 and Supplementary Fig. 36). The performance of Racon was highly variable but always less effective than either MEGAN-LR or Medaka. MEGAN-LR generally provided the best performance, followed by the multiple procedure approach, then Medaka.

We further examined the influence of sequence correction on genome quality statistics, as estimated by CheckM (Table 2). Of the 22 frame-shift corrected genomes classifiable as high quality (Table 1), 3, 14, 7 and 17 of these were also classifiable as high quality when examined in their uncorrected, Medaka–corrected, Racon–corrected and multiple procedure corrected forms (Table 2), with the mean completeness being 76.8% (range: 41.9–93.0), 91.8% (range: 78.0–98.0), 86.9% (range: 66.0–97.0) and 92.1% (range: 77.2–98.0), respectively, compared to a mean of 95.3% (range: 92.6–99.0) in the case of MEGAN-LR. Contamination was never observed to be greater than 2.7% in any version of the 22 genomes.

**Table 2:** Influence of sequence correction procedure on CheckM–derived genome quality statistics.

Quality measure	Completeness^a^					Contamination^a^				
	Uncorrected	MEGAN-LR	Medaka^b^	Racon^c^	Multiple^d^	Uncorrected	MEGAN-LR	Medaka^b^	Racon^c^	Multiple^d^
PAO1-tig00000001	84.28	**95.28**	**93.91**	87.88	**94.71**	0.66	**1.11**	**1.59**	1.62	**1.11**
PAO1-tig00000003	64.34	**92.82**	84.84	78.21	87.74	1.56	**0.25**	0.90	0.74	0.74
PAO1–tig00000117	82.46	**97.53**	**94.82**	**90.99**	**94.82**	0.15	**0.15**	**0.15**	**0.15**	**0.15**
PAO1–tig00026549	86.57	**94.64**	**97.13**	**91.45**	**96.73**	0.50	**0.50**	**0.50**	**0.50**	**0.50**
PAO1-tig00026557	75.56	**94.19**	**95.24**	86.16	**95.58**	2.70	**1.58**	**1.58**	1.58	**1.58**
PAO1–tig00026560	71.51	**93.99**	**94.24**	86.70	**94.52**	1.59	**0.55**	**0.00**	0.30	**0.00**
PAO1–tig00198536	74.83	**92.57**	**94.64**	88.68	**94.39**	2.74	**1.98**	**2.48**	2.23	**2.48**
PAO2-tig00000001	70.90	**93.85**	89.33	83.64	**90.88**	0.03	**0.00**	0.03	1.19	**0.03**
PAO2–tig00000013	45.13	**94.49**	89.16	86.32	**91.52**	0.00	**0.18**	0.59	0.38	**0.12**
PAO3A–tig00000003	75.75	**94.56**	**93.78**	83.48	**93.52**	0.10	**0.28**	**0.50**	0.10	**0.50**
PAO3A–tig00000011	82.70	**94.31**	**93.70**	89.08	**95.05**	2.23	**2.17**	**2.26**	1.44	**1.91**
PAO3A–tig00000024	79.79	**96.92**	89.46	84.66	89.86	0.81	**0.00**	0.32	0.32	0.32
PAO3A–tig00000209	87.00	**95.38**	**96.80**	**94.60**	**97.32**	0.00	**0.00**	**0.00**	**0.00**	**0.00**
PAO3A–tig00018026	41.99	**93.47**	81.67	66.02	77.17	0.28	**1.09**	1.09	1.27	1.37
PAO3A–tig00139797	**91.56**	**99.04**	**97.05**	**97.02**	**97.05**	**0.48**	**0.48**	**0.55**	**0.48**	**0.48**
PAO3B–tig00000024	89.02	**98.10**	**93.63**	**90.53**	**91.71**	0.23	**0.23**	**0.23**	**0.00**	**0.39**
PAO3B–tig00000027	73.78	**93.16**	85.11	81.80	87.97	0.42	**0.42**	0.63	0.16	0.73
PAO4–tig00000001	**93.04**	**98.64**	**97.97**	**96.58**	**97.95**	**1.90**	**2.07**	**2.07**	**2.06**	**2.06**
PAO4–tig00000030	76.77	**97.10**	**91.63**	85.14	**91.87**	1.30	**2.30**	**1.52**	1.63	**1.11**
PAO4–tig00000046	**91.85**	**96.30**	**97.64**	**94.90**	**97.40**	**0.47**	**0.47**	0.47	**0.50**	**0.47**
PAO4–tig00000079	80.26	**94.79**	89.66	89.85	**90.87**	0.00	**0.00**	0.66	0.72	**0.66**
PAO4–tig00000228	70.80	**94.81**	77.98	77.13	78.18	0.02	**0.00**	0.08	0.00	0.00

Versions of genomes shown in bold demonstrate CheckM completeness > 90% and contamination < 5%.

^a^Genome completeness and contamination estimates obtained from *CheckM* (see Materials and Methods).

^b^Medaka: single round of correction applied to each uncorrected genome sequence.

^c^Racon: single round of correction applied to each uncorrected genome sequence, run with default parameters for all datasets.

^d^Multiple: four sequential applications of Racon (run with default parameters using long read data) and then one application of Medaka (run with default parameters; the following models were used -m r941_min_high_g303 for PAO1, PAO2, PAO3A and PAO3B and -m r941_min_high_g330 for PAO4).

### Taxonomic analysis of recovered genomes

We inferred taxonomy of the recovered genomes against the Genome Taxonomy Database (GTDB)^36^ using the GTDB-Tk toolkit^37^ (see Materials and Methods: Analysis of long read sequence data and Supplementary Data 2). Of the 22 long read genomes, five had sufficiently high degree of similarity, defined as >95% Average Nucleotide Identity (ANI) to a representative genome^36^, to be classified to species level (for these five genomes the mean ANI was 97.1%). Of the remaining genomes, 11 were annotated to genus level, 5 to family level and 1 to class level (Table 1 and Supplementary Data 2). We also examined the taxonomic annotation of all full length 16S-SSU-rRNA gene sequences from all 22 genomes (Supplementary Data 3), observing that, in the case of 10 genomes the whole-genome GTDB annotation and the SILVA 16S-SSU-rRNA gene annotation where consistent at the genus level. In the case of the remaining genomes, 8 showed consistency at family level, 2 at order level and 2 at class level (Supplementary Data 4).

**Fig. 3:**
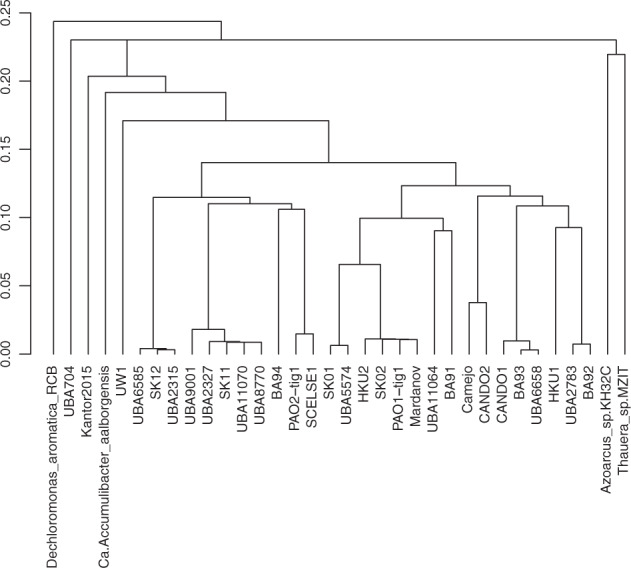
Dendrogram generated from MASH distances between draft genomes of *Candidatus* Accumulibacter, including two genomes recovered in the present study. Genomes from genera *Thaurea*, *Azozarcus* and *Dechloromonas* were used as an outgroup. Note the structure of the tree recapitulates previously defined clade associations (*Clade IIF*: BA94, SK11, SK12; *Clade IIC*: BA91, SK-02, SK01; *Clade I*: BA92 and BA93. With UW1 being a singleton for *Clade IIA*). See Supplementary Material for GenBank assembly accession identifiers corresponding to node labels.

We recovered genomes of five taxa that hold known relevance to wastewater bioprocesses, namely two genomes from the PAO species *Ca*. Accumulibacter: the PAO1-tig00000001 genome was closely related to *Ca*. Accumulibacter sp. SK-02^38^, and was also found in our previous analysis of the PAO1 data^16^, and the other (PAO2-tig00000001) was similar to *Ca*. Accumulibacter sp. BA94^38^ (Fig. 3) and a short read MAG previously recovered by us and denoted as *Ca*. Accumulibacter clade IIF Strain SCELSE-1^39^.

We recovered two genomes from members of genus *Dechloromonas*, generally considered as exhibiting the PAO phenotype^28^. Genomes from several organisms exhibiting the glycogen-accumulating organism (GAO) phenotype were recovered from genus *Micropruina*^40,41^ and genus *Ca*. Contendobacter^42^. The PAO1–tig00026549 sequence, annotated to the novel GTDB-derived family 2–12–FULL–67–15 and harbouring a 16S gene annotated to *Defluviicoccus* (Supplementary Data 3), represents a novel member of the latter genus, whose members exhibit the GAO phenotype^43^.

We also recovered a genome from a member of genus *Thiothrix*, a filamentous bacterium associated with the maintenance of floccular structure in activated sludge biomass^44,45^.

A set of four genomes recovered here have been previously identified in temperate climate activated sludge, namely three members of the CFB group recovered from the PAO1 data, OLB8^46^, OLB11 and OLB12^46^, as well as a genome classified to the *Rhodobacteraceae* genus UBA1943^47^. *Thiobacillus* has been previously identified in activated sludge from industrial wastewater treatment plants^48,49^. In the PAO4 community, we recovered a genome close to that of *Exiguobacterium profundum*, originally discovered in deep-sea hypothermal vents^50^. Members of this genus, namely *Exiguobacterium alkaliphilum* and *Exiguobacterium* sp. YS1, have been studied in relation to treatment of high alkaline brewery wastewater and solubilisation of activated sludge, respectively^51,52^. The genome of a member of family *Parachlamydiaceae*, an environmental *Chlamydia*^53^ previously recovered by us^16^ from the PAO1 dataset, is probably a symbiont species of protists that are known to inhabit activated sludge^54^. The genome of *Brevundimonas* was closely related to a short read MAG previously obtained from an activated sludge metagenome in Hong Kong^55^, and members of this genus have been observed previously in activated sludge systems^56^, where they have been associated with quinoline degradation from coking wastewater^57^.

A genome from a member of genus *Pseudoxanthomonas* was also recovered. The remaining genomes had no close references, and likely represent uncharacterised members of the microbial groups, namely family *Nocardioidaceae* (tig00157979 from PAO3B), from class *Anaerolineae* (tig00018026 from PAO3A), family *Burkholderiaceae* (tig00000024 from PAO3B) and a genome from the novel UBA6002 family (tig00000117 from PAO1).

### Further refinement of selected genomes

We applied manual refinement procedures, as described in Materials and Methods, to the two recovered genomes of *Ca*. Accumulibacter, namely PAO1-tig00000001 and PAO2-tig00000001, in order to obtain submission quality finished genomes. Detailed notes on refinement and manual curation procedures are provided in the Zenodo submission. For the PAO1-tig00026557 sequence, the first 288,176 bp were removed following their identification as an artefactual concatenation, as described above.

### Identification of non-chromosomal replicons and short microbial chromosomes

We identified 96 LRAC sequences less than 1Mbp in length that were classified as circular constructs, and these are plausible candidates for being plasmids^58^, phages/viruses^59^ or small microbial genomes characteristic of the Candidate phyla radiation (CPR) group^60^. These are summarised in Supplementary Data 5. Of the 96 sequences, four pairs of closely related sequences were observed from the PAO3A and PAO3B datasets, based on analysis with the dRep workflow (Supplementary Fig. 37).

Of these 96 sequences, three harboured both a complete ribosomal RNA operon and at least some tRNA genes, consistent with being chromosomal sequences. One such sequence (PAO4-tig00000333, of length 848,000 bp, with 1 complete rRNA operon and 46 tRNA genes) was identified as a previously undetected member of order *Saccharimonadales* (superphylum *Patescibacteria*)^61^. The other two sequences (PAO3A–tig00000268 and PAO3B–tig00001295) have low numbers of tRNA genes observed (8 and 3, respectively) and therefore may be chromids^62^. Although one of these sequences (PAO3A-tig00000268) was classified as a virus by the CheckV workflow^63^, the presence of an rRNA operon would suggest this is more likely related to the presence of a prophage sequence.

We observed further a subset of four sequences (PAO2-tig00000067, PAO2-tig00000097, PAO2-tig00000115 and PAO2-tig00000125) that were extremely short, and only contained hypothetical genes (none which were not classified as viral sequences by CheckV). We considered these to be artefactually circularised fragments of longer genomes.

Of the remaining 89 sequences, CheckV classified 38 as viruses/phages and 3 as prophages. For the remaining 48 sequences, we therefore considered them as putative plasmids. Ten of these we considered putative conjugative plasmids due to the presence of genes from the Type IV secretion system (T4SS)^64^ and genes known to be involved in plasmid replication. Two closely related plasmids (PAO3A–tig00002252 and PAO3B–tig00158345) showed BLASTN query coverages of 83% and 89% against a plasmid genome (NCBI RefSeq NZ_CP014059.2) previously obtained from a culture isolate of *Achromobacter xylosoxidans* (Supplementary Data 6). Some sequences classified as viruses showed query coverage scores of up to 50% when compared against virus reference databases (Supplementary Data 7). Screening all 96 sequences for the presence of KEGG Orthology annotated genes associated with plasmids or viruses, we observed a reasonable degree of partitioning between the two categories (Supplementary Data 5 and Supplementary Fig. 38). Collectively, these data suggest that viruses, phages and plasmids, along with small microbial genomes, can be recovered using long read sequence from complex microbial communities.

### Comparison against hybrid metagenome assembly workflows

Several metagenome assembly workflows have recently been developed that make use of long and short read data in combination (procedures that are referred to as *hybrid metagenome asembly* in the literature). While the analyses reported above have placed emphasis on long read metagenome assembly, we also examined the performance of two hybrid metagenome assembly workflows, OPERA-MS^18^ and hybridSPAdes^65^, on the long and short read data sourced from each of the four bioreactors (summarised in Supplementary Table 6). We first examined the total number of sequences generated that were at least 1 Mbp in length (that is, equivalent to our definition of LR-chr for long read assemblies, and referred to as HY-chr hereon), observing 72 and 78 HY-chr sequences were generated by OPERA-MS and hybridSPAdes, respectively, which was less than the number of LR-chr obtained from Canu (*n* = 90) but more than obtained from either Flye or Unicycler (Supplementary Data 8). The HY-chr sequences recovered from both hybrid workflows tended to be shorter (mean length: 1,548,471 bp and 1,678,095 bp for OPERA-MS and hybridSPAdes, respectively) than those obtained from Canu (mean length: 2,670,352 bp). Most of the HY-chr sequences appeared to constitute partial genome fragments, with only 8/150 (5.3%) sequence holding putative high quality status (2 from OPERA-MS and 6 from hybridSPAdes, respectively) (Supplementary Data 8).

To examine the extent to which these hybrid procedures generated complementary sequence to that obtained from long read assembly alone, we examined the degree of similarity among the combined set of 240 LR-chr and HY-chr sequence using the dRep workflow (Supplementary Data 9). From this set of sequences 134 secondary clusters were obtained. Of these 42 (31.3%), 17 (12.7%) and 20 (14.9%) were comprised of LR- or HY-chr sequences that arose only from Canu, OPERA-MS and hybridSPAdes, respectively. Twelve secondary clusters (9%) were comprised of sequences that were generated by all three workflows, with 15 (11.2%), 15 (11.2%) and 13 (9.7%) secondary clusters being generated from sequences obtained from Canu and OPERA-MS, Canu and hybridSPAdes, and OPERA-MS and hybridSPAdes, respectively (Supplementary Data 9 and Supplementary Fig. 39).

Of the eight complete genomes obtained from hybrid assemblies, six were cognates of genomes recovered by long read assembly (Table 1 and Supplementary Data 9). Only one of these eight, a member of the novel Alphaproteobacteria family UBA1301 (Supplementary Data 8), was not recovered from the application of long read assembly and the eighth, a member of genus OLB17 in family *Pyrinomonadaceae* (recovered by hybridSPAdes), was cognate to part of the artefactual contig generated by Canu (PAO3A–tig000000001) described above (see dRep secondary cluster 48_1 in Supplementary Data 8).

In summary, and conditional on the underlying microbial community complexity of the communities studied here and the sequencing depths obtained, hybrid assembly procedures return a lower rate of complete chromosomal genome sequence compared to using long read assembly, but they do capture chromosomal length sequences from species that are not obtained from the latter approach.

## Discussion

In this paper we explore how long read metagenome data, generated by a Nanopore MinION sequencer, can enhance the recovery of member genomes of microbial communities. Building on our previous analyses^16^, we obtain further data from activated sludge enrichment bioreactor communities, and obtain 22 non-redundant complete microbial chromosomes, of which nine are closed (circular) and six are from species with key functional relevance to wastewater bioprocesses. Additionally, we demonstrate the complete recovery of a short microbial genome from order *Saccharimonadales*, as well as probable phage and plasmids sequences present in these communities. We also present further details of methodology for assessing whether genomes obtained from short read assemblies recapitulate those obtained from assembled long read data, and examine aspects of genome quality not previously covered, including the quality of gene coding sequence and the sequences arising from mis-assembly and related artefacts. Further, we demonstrate that for the communities studied here, hybrid metagenome assembly does not provide improved performance in relation to overall genome recovery compared to the use of metagenome assembly on long read data alone. These new results highlight that by using long read sequencing in microbial communities of moderate complexity, it is clearly feasible to capture sequence constructions that are close to the requirements of high quality, closed genomes, of some abundant community members, without the use of contig binning procedures. However careful evaluation of such genomes still appears mandatory to assess overall quality and the presence of artefactual constructs.

The widespread use of metagenome-assembled genomes (MAG) methodology on short read metagenome data has provided a tremendous number of new draft genomes from diverse microbiomes and microbial communities (for example^19,66^ among others). However substantial limitations of this approach have become evident, including problems related to the use of multi-sample co-assemblies^19,66,67^, the challenges of resolving genomes to strain level^68^, difficulties related to extracting MAGs from communities of high ecological complexity^69,70^ and the limitations of automated binning procedures, requiring careful evaluation of recovered genomes^71^. In response to these challenges, recent efforts have combined short read with emerging complementary techniques such as HiC metagenomics^72–74^, synthetic long reads^75,76^, or long read sequencing^12,16–23^ and collectively these results suggest substantial improvements can be made in the quality and completeness of metagenome-assembled genomes using multiple types of sequence data.

In the present study, we make use of DNA extractions that are co-assayed with both long and short sequencing, or DNA extractions from sampling events close enough together in time, that we can discount the influence of the ecogenomic differences as major influence on any observed differences between the types of sequence data. Our analysis proceeds on the basis that neither short read nor long read data can be assumed to provide an accurate reference genome, and so we seek to understand and characterize the degree of agreement between assembled sequence generated from each data source. Although error prone MinION sequence can be corrected using higher quality short read sequences^77^, we have deliberately kept the two sources of data separate so as to not introduce any positive bias in the calculation of the concordance statistics. The concordance statistic was developed to provide a straightforward screening procedure for identifying short read MAGs that are cognate to assembled genomes from long read data, by capturing information from alignment statistics. The concordance statistic also may have broader utility, for example we can observe several instances of ’split’ bins from the short read assembly that are cognate to a given long read assembled genome, and cases where assembled long read sequence is demonstrably artefactual. We highlight that the concordance statistics capture more information than are contained in dot-plots (which require the imposition of arbitrary decision thresholds on alignment statistics), albeit at the cost of increased complexity. We provide R code for computing the concordance statistics from alignment results, and example workflows for visualisation.

While these are clearly vast improvements on the working models of genomes available from short read MAG analysis, several problems are still present that require attention and/or explicit correction. First, the high error rate implicit in MinION sequence generally not less than 5% sequencing error^78,79^, requires correction procedures to be applied either pre- or post-assembly, as already mentioned. In the present case, we are relying on the frame-shift correction algorithm implemented in MEGAN-LR^13,16^, which appears to perform slightly better than the next best correction procedure (the workflow described as ’multiple’ above). As previously discussed^16^, the use of this correction procedure results in better performance of existing genome quality workflows (CheckM in the present case), and the resulting sequence can be considered to be at least a high quality assembly under currently accepted criteria^35^. However further analysis of the corrected gene content suggests there remains a substantial proportion of genes that remain inadequately corrected when compared against genes predicted from the cognate short read assemblies. Because the MEGAN-LR correction is dependent on aligned sequence from database comparisons, a combination of false positives alignments and a lack of closely related reference genomes could result in inappropriate or inadequate correction of the query sequences in our analysis, and additionally mis-assembly of genes in the short read assembly (subject sequences) could also be a factor in patterning these findings. A second factor relates to the inclusion of artefactual sequence (mis-assembly), which we identify and remove using examination of read alignment and coverage profiles, in line with recent calls for more careful evaluation of the output of automated genome recovery procedures^71^. These results indicate that long read sequencing technology should be considered complementary to short read sequencing for studies on genome-resolved metagenomics until sequencing error rates decrease to more acceptable levels.

We have deliberately focused on long read assembled contigs that form single contiguous sequences that are consistent with being whole-bacterial chromosomes or accessory genomes (phages, plasmids, possibly chromids), a decision which plays to the full strengths of long read sequencing. The remaining subset of contigs, that do not meet our criteria for being considered putative genomes or non-chromosomal replicons, will be in part comprised of genome fragments that could be recovered into draft genomes using binning methods. We also show that while hybrid metagenome assembly generally did not recover complete chromosomal sequences, it does capture chromosomal length sequences that are not reconstructed from the use of long read data alone. Despite the fact that long read metagenome data collected from microbial communities of high to very high complexity are still emerging^18,20,22,23^, the analysis of datasets available to date suggests that binning procedures will have to be developed^18^ or adapted from short read methods^20,22^, for effective recovery of genomes from such metagenome data.

Our analysis of circular sequences less than 1 Mb in length shows the feasibility of capturing short microbial genomes as well as non-chromosomal replicons from whole-community long read sequencing. The categorisation of these sequences remains challenging and subject to ambiguity, consistent with our rapidly changing knowledge of such replicons^59^ and by a relative lack of study in the context of environmental settings^58^.

In this study, we have made use of recent developments in genome–based microbial taxonomy provided by the Genome Taxonomy Database (GTDB)^36^. The use of this resource permits taxonomic assignments to be made using whole-genome sequence, and which led to some genomes being classified to species level (Table 1). While this development provides an important advance compared to the use of 16S-SSU-rRNA based annotation, the different annotations systems are currently incommensurate and/or under rapid development, which can clearly impact interpretation. For example, in the present case less than 50% of genomes obtained had GTDB and SILVA annotations consistent at genus level (Supplementary Data 4). Another example can be seen in our comparative analysis of LR-chr and HY-chr sequence, which used a subsequent version of GTDB than that used to originally classify the LR-chr sequences, and where both Defluviicoccus (LR-chr) genomes were reclassified as being from the closely related genus *Azonexus*, despite their previous consistency at genus level with 16S classification (see Supplementary Data 8). More broadly these differences reflect the rapidly changing landscape of microbial taxonomy and systematics, including the need to deal with the increasingly large numbers of draft genomes being obtained from metagenome surveys, the need for more flexible nomenclature systems^80,81^ and the ongoing issue of how DNA sequence in general, and whole-genome sequence in particular, can be incorporated into the official procedures of prokaryotic systematics^82^. While we recognise that technical improvements in metagenome quality are only a small part of this landscape, it is worth highlighting that the kind of genomes recovered here would be virtually indistinguishable in quality from those obtained by the analysis of genomic DNA from cultured type material, and furthermore that both metagenome sequence data, and whole-genome sequence derived from them, are stable entities under any reasonable definition.

In the present study, we are able to draw strength from the fact that the communities under study are of moderate complexity, and in ecological terms, are of low evenness, compared to the source inoculum, namely activated sludge residing in full-scale wastewater treatment plants^26^. This suggests that one way to approach the systematic genome-resolved dissection of such complex communities would be to simply sample a diverse array of such enrichment communities, rather than rely on more deeper, expensive sequencing of a limited number of highly complex source communities (and indeed this approach has been useful in other settings, for example, in soil microcosm enrichments^27^). While such an approach may miss some relevant species, due to the biases of enrichment protocols, it would permit the recovery of many near-finished genomes from key species of direct functional relevance to wastewater bioprocess engineering.

## Methods

### Enrichment bioreactors and biomass sampling

We employed the biomass from a series of enrichment reactor microbial communities, each from activated sludge sourced from wastewater treatment plants located in Singapore. We sampled biomass via the in-built sampling port of each reactor vessel. Unless otherwise stated, 2 mL of suspended biomass was obtained and the DNA extraction protocol (see below) was commenced immediately afterwards. We sampled the following enrichment reactor communities:

(i) A lab-scale sequencing batch reactor, inoculated with activated sludge from a full-scale wastewater treatment plant (Public Utilities Board, Singapore), was operated using acetate as the primary carbon source to enrich for PAO species. The reactor was sampled on day 267 of the operation, with both long read (Nanopore MinION) and short read (Illumina Miseq 301 bp PE) sequencing data from the same DNA aliquot. These data have been previously published by us^16^ and are available via NCBI BioProject accession PRJNA509764. This dataset is referred to below as the *PAO1* data.
(ii) A lab-scale sequencing batch reactor, inoculated with activated sludge from a full-scale wastewater treatment plant (Public Utilities Board, Singapore), was operated using alternative carbon sources to enrich for PAO species. The reactor was sampled on April 6, 2018, gDNA extracted and both long read (Nanopore MinION) and short read (Illumina HiSeq2500 251bp PE) data obtained from the same DNA aliquot. These data are available available via NCBI BioProject accession PRJNA611629. This dataset is referred to below as the *PAO2* data.
(iii) An enrichment targeting putative PAO species, namely members of genera *Tetrasphaera* and *Dechloromonas*. Following inoculation with activated sludge from a full-scale wastewater treatment plant (Public Utilities Board, Singapore), the reactor was fed with synthetic wastewater containing either glutamate or glucose as the main carbon source, with the feed type switched in a weekly manner, and operated at 31 °C. Short read data (Illumina HiSeq2500 251bp PE) had been previously obtained from sampled biomass on days 272, 279 and 286 of operation, and long read data (Nanopore MinION) obtained from samples taken on days 264 and 293 of operation. These samples had been snap frozen in liquid nitrogen and stored at −80 °C. They were thawed on ice immeditately prior to the commencement of the DNA extraction protocol. These data are are available via NCBI BioProject accession PRJNA606905. The long read data obtained from each sampling day is referred to below as the *PAO3A* and *PAO3B* data, respectively.
(iv) An enrichment targeting PAO species capable of performing denitrification. A lab-scale sequencing batch reactor was inoculated with activated sludge from a full-scale wastewater treatment plant (PUB, Singapore). The reactors were operated at 35 °C using acetate as the primary carbon source fed under anaerobic conditions, but without the addition of allyl-thiourea (ATU) in order to suppress the growth of ammonia oxidizing bacteria, with the aim of targeting polyphosphate-accumulating organisms that could also reduce nitrogen oxides (nitrite and/or nitrate). For this study we obtained long read data (Nanopore MinION) and short read data (Illumina HiSeq2500 251bp PE) from the same DNA aliquot extracted from biomass sampled on day 292 of operation. These data are available via NCBI BioProject accession PRJNA607349. These data are referred to below as the *PAO4* data.

### DNA extraction

Genomic DNA in the case of the samples from PAO1, PAO3A, PAO3B and PAO4 was extracted from the sampled biomass as described previously by us^16^, briefly, we used the FastDNA^TM^SPIN Kit for Soil (MP Biomedicals), using 2 × bead beating with a FastPrep homogenizer (MP Biomedicals). Extracted gDNA from the PAO1, PAO3A and PAO3B samples was then size-selected on a BluePippin DNA size selection device (SageScience) using a BLF-7510 cassette with high pass filtering with a 8 kbp cut-off. The gDNA from the PAO4 sample was size-selected using Circulomics Short Read Eliminator XS kit (Circulomics Inc). Size-selected DNA was then taken for Nanopore library construction (see below).

From the biomass from PAO2 sampling, high molecular weight (HMW) DNA was extracted using a modified xanthogenate-SDS protocol^83^. Briefly, 2 mL of biomass from lab-scale sequencing batch reactor was harvested by centrifugation, the pellet was resuspended in 0.6 mL of DNA/RNA shield (Zymo Research) and added to 5.4 mL of pre-heated (65 °C) XSP buffer (1:1 volumes of XS buffer and phenol). The tubes were incubated at 65 °C for 15 min, vortexed for 10–15 sec, placed on ice for 15 min and centrifuged at 14,000 rpm for 5 min. The aqueous phase was transferred to a fresh tube and extracted with equal volume of phenol:chloroform:isoamyl alcohol (25:24:1) followed by extraction with chloroform:isoamyl alcohol (24:1). The aqueous phase after chloroform:isoamyl alcohol (24:1) extraction was ethanol precipitated and resuspended in TE buffer. The extracted DNA was further treated with RNase A (Promega) then extraction with phenol, followed by phenol:chloroform:isoamyl alchohol (25:24:1), and ethanol precipitation. Purified DNA was taken to library construction for Nanopore sequencing.

### Short read sequencing

Genomic DNA Library preparation was performed using a modified version of the Illumina TruSeq DNA Sample Preparation protocol. We then performed a MiSeq sequencing run with a read length of 301 bp (paired-end) or a HiSeq2500 sequencing run with a read length of 251 bp (paired-end) as specified above.

### Long read sequencing

Nanopore sequencing was performed on a MinION Mk1B instrument (Oxford Nanopore Technologies) using a SpotON FLO MIN106 flowcells and R9.4 chemistry. Data acquisition was performed using MinKNOW software, without live basecalling, running on a HP ProDesk 600G2 computer (64 bit, 16 GB RAM, 2 Tb SSD HD; Windows 10). The runs were continued until active pores in flowcells were depleted. For PAO1, PAO3A and PAO3B extractions, the sequencing library was constructed from ~4–4.5 μg of size-selected genomic DNA using SQK-LSK 108 Ligation Sequencing Kit and ~900 ng of the library was loaded onto each flowcells. For PAO2 dataset, sequencing libraries were constructed from HMW DNA using two different sequencing kits from ONT. The first kit was the Rapid Sequencing kit SQK-RAD004, for which the library was constructed from 400 ng of HMW DNA and the entire library loaded onto the flowcell. The second kit was the Ligation Sequencing kit SQK-LSK 108, for which 1.0 μg of genomic DNA was used for library construction, and 400 ng was loaded onto the flowcell. For PAO4 dataset, the sequencing library was constructed from 1.2–1.3 μg of size-selected DNA using SQK–LSK109 Ligation Sequencing Kit (Oxford Nanopore Technologies). The library was diluted to allow 250 ng of the library to be loaded on the flowcell.

### Analysis of long read sequence data

Basecalling was performed with guppy (CPU version 3.2.1, 3.2.2 or 3.3.0 for Linux 64-bit machines; see Table S1). Adaptor trimming was performed using Porechop (version 0.2.2)^84^ with default settings except -v 3 -t 20. We assembled long read data using Canu (version 1.8 or 1.9, default settings except corOutCoverage=10000, corMhapSensitivity=high, corMinCoverage=0, redMemory=32, oeaMemory=32 and batMemory=200 useGrid=false)^29^, Unicycler (version 0.4.7 or version 0.4.8 with default settings except -t 20 --keep 3)^30^ and Flye (version 2.4 with default settings except -t 20 --plasmids --debug --meta)^31^. Contigs generated from long read data at least 500 bp in length were denoted as long read assembled contigs (LRAC). The number of reads used in each assembly was estimated by mapping long read to LRAC sequence with minimap2 (version 2.17)^85^ and using samtools-1.6 to calculate the number of aligned reads^86^.

We used DIAMOND (version 0.9.24)^87^ to perform alignment of LRAC sequences (with default settings except -f 100 -p 40 -v --log --long-reads -c1 -b12) against the NBCI–NR database (February, 2019)^88^. From the MEGAN Community Edition suite (version 6.17.0)^89^ we used daa-meganizer (run with default settings except --longReads, --lcaAlgorithm longReads, --lcaCoveragePercent 51, --readAssignmentModealignedBases and the following settings for mapping files: --acc2taxaprot_acc2tax-Nov2018X1.abin, --acc2eggnogacc2eggnog-Oct2016X.abin, --acc2interpro2go acc2interpro-June2018X.abin, --acc2vfdb acc2vfdb-Feb2019.map) to format the .daa output file for use in the MEGAN GUI (version 6.17.0). Within MEGAN, LRAC sequences were exported with the ‘Export Frame-Shift Corrected Reads’ option to obtain frame-shift corrected sequence. As described above, LRAC sequence that was at least 1Mbp in length were categorised as potential whole-chromosome sequence and denoted as *LR-chr* sequence. Five LR-chr sequences generated from Canu were not exported during when the ‘Export Frame-Shift Corrected Reads’ option was run in MEGAN, and we subsequently obtained these sequences using updated versions of the mapping files in daa-meganizer, as specified by the following arguments --acc2taxa prot_acc2tax-Jul2019X1.abin, --acc2eggnog acc2eggnog-Jul2019X.abin, --acc2interpro2go acc2interpro-June2018X.abin, --acc2vfdb acc2vfdb-Feb2019.map. One of these 5 LR-chr sequences was classified to be a putative genome annotated to genus Contendobacter (Table 1).

We processed LR-chr sequences with CheckM (version 1.0.11)^32^ and Prokka (version 1.13)^34^ to assess genome quality. LR-chr sequences that demonstrated CheckM-SCG completeness >90% and contamination <5% were classified as putative genomes. The entire set of putative genomes were de-replicated using the dRep (version 2.2.3) workflow^33^ with the following settings: -p 44 -comp 90 -con 5 -str 100 --genomeInfo. We performed taxonomic annotation on recovered genome sequence using GTDB-Tk (version v0.3.2 and GTDB-Tk reference data version r89, running default parameters except --cpus 40 -x fasta)^37^. Coverage profiles were generated from both long read and short read data, by mapping each of these to LR-chr sequences using minimap2 (version 2.17) using the following flags -ax map-ont -a -t 20 for long read data, and -ax sr -a -t 20 for short read data. Sorted .bam files were subsequently processed using bedtools genomeCoverageBed (version 2.26.0) with the following flags -d. We extracted 16S-SSU-rRNA genes as identified with Prokka and annotated them against SILVA database (SURef_NR99_132_SILVA_13_12_17_opt.arb)^90^ using sina-1.6.0-linux^91^ running default settings except -t -v --log-file –meta-fmt csv and with --lca-fields set for all five databases, namely tax_slv, tax_embl, tax_ltp, tax_gg and tax_rdp.

### Analysis of short read sequence data

The raw FASTQ files were processed using cutadapt (version 1.14)^92^ with the following arguments: --overlap 10 -m 30 -q 20,20 --quality-base 33. We performed metagenome assemblies from short read data using SPAdes (version 3.12.0-Linux or 3.14.0-Linux, with default settings except -k 21,33,55,77,99,127 --meta)^93^ either as single sample assemblies, in the case of short read data from PAO1, PAO2 and PAO4, or as co-assembly of all short read in the case of the PAO3A and PAO3B samples. The contigs generated from short read data at least 500 bp in length are hereafter denoted as short read assembled contigs (SRAC). We identified putative member genomes using MetaBAT2^94^, after filtering for contigs at least 2000 bp in length. We identified 16S genes within contigs using the --search16 module of USEARCH (version 10.0.240, 64 bit)^95^, and annotated them using the SILVA::SINA webserver (using default parameters)^91^. For each identified bin we performed genome quality estimation using CheckM (version 1.0.11). We performed taxonomic annotation on recovered member genomes using GTDB-Tk (version 0.3.2 and GTDB-Tk reference data version r89, running default parameters except --cpus 40 -x fasta. See Supplementary Data 10).

### Comparative analysis of long and short read assemblies

We used BLASTN (version 2.7.1+)^96^ to examine the degree of sequence alignment between LRAC and SRAC sequences. We treated the LRAC as the subject sequences and the SRAC as the query sequences, using default BLASTN parameters (except -outfmt 6). From the BLASTN tabular output, we retained the highest bit-score from each unique combination of query and subject pair. In order to identify the short reads bin(s) that are cognate to a given LR-chr sequence, we then computed the concordance statistic (*κ*), as previously described by us^16^, for all combinations of short read contigs (categorised by bin membership) and LRAC sequence, that were present in the BLASTN output. We then compute the following component statistics:

1. $\widehat{{\mathit{pid}}}$: The mean of the percent identity (PID), calculated across alignments, and quantified as a proportion. $\widehat{pid}$ is defined on the interval [0,1]
2. $\widehat{{\mathit{al2ql}}}$: The mean of the quotient of the alignment length to the query length, calculated across alignments, and quantified as a proportion. $\widehat{{\mathit{al2ql}}}$ is always ≥0 and while values >1 can be observed, in practice the maximum observed value be ~1.
3. *p*_*srac*_: the quotient of the number of short read contigs in the bin that produce alignments and the total number of short read contigs in the bin. *p*_*srac*_ is defined on interval [0,1]
4. *p*_*aln*_: the proportion of the long read contig that is covered by an alignment. *p*_*aln*_ is defined on the interval [0,1].

Collectively these statistics contain information on how well a set of short read contigs will tile a LR-chr sequence, namely, completeness of coverage (captured by *p*_*srac*_ and *p*_*aln*_), as well as quality of the alignments (captured by $\widehat{{\mathit{pid}}}$ and $\widehat{{\mathit{al2ql}}}$). We can hypothesise that if the majority of the contigs in a short read MAG completely covered a LR-chr sequence with high quality alignments, we would predict all four of these statistics would hold values close to unity. A simple extension of this prediction is to calculate the mean of the four statistics, which we denote as the concordance statistic, $\kappa =(\widehat{{\mathit{pid}}}+\widehat{{\mathit{al2ql}}}+{p}_{{\mathrm{srac}}}+{p}_{{\mathrm{aln}}})/4$, which provides a single number to screen large numbers of pairwise combinations of short read and long read derived MAG in an efficient way. The concordance statistic (*κ*) was computed using all alignments, as well as after filtering for near-full length alignments (defined as *al*2*ql*≥0.95). We provide an R package srac2lrac to compute *κ* (along with all component statistics) following calculation of BLASTN–like alignment statistics and definition of short read bins.

### Analysis of effects of frame-shift correction on coding sequence

We analysed the distribution of ratio of predicted gene length to the length of the nearest orthologous gene^97^, before and after the application of frame-shift correction procedures, as well as for two other sequence correction algorithms, namely Medaka (version 0.11.5)^98^ and Racon (version 1.4.3)^99^. In the first instance, we employ a single round of correction and in the case of Racon we do not use short read data for correction (in order to maintain independence of each data type, as in the case of the concordance statistic calculations). MEGAN-LR frame-shift correction procedure uses the results of alignments made against RefSeq NR, we did not compare against this same database to avoid positively biasing the performance, rather we used predicted genes from the cognate short read assemblies as a database of genes to use as subject sequences. Specifically, we took the protein coding sequence of each ORF in each of four versions of the genome generated above, and performed homology search of each sequence against the short read assembly ORF database using DIAMOND (version 0.9.24, running in blastp mode with default parameters except -f 6 qseqid qlen slen sseqid sallseqid -p 40 -v --log --max-target-seqs 1). We then calculated the quotient of the length of the query sequence to the length of subject sequence holding the maximum bit-score (best hit). We ran CheckM on each of the four versions of each putative genome, as described above. We also examined the common practice of applying multiple rounds of correction using four sequential applications of Racon followed by one application of Medaka (denoted as ‘multiple’ from hereon): as above we do not employ short read sequence when running Racon.

### Procedures for refining draft genomes

In LR-chr sequence we screened regions of potential mis-assembly by identifying genomic intervals of at least 10 bp in length, where long read coverage was either abnormally high or abnormally low, defined as >1.5 of the median coverage and <0.5 of the median coverage, respectively. We then examined alignments of both long and short read data to the genomes using the Integrated Genome Viewer (IGV version 2.4.14)^100^ to identify low coverage regions that showed evidence of misconnection between reads, or weakly supported connection, or in the case of high coverage regions, to disambiguate types of read connections likely to arise from non-cognate sources. We generated VCF files for short read alignments using BCFtools (version 1.9 run with flags -mv)^101^ to identify likely single nucleotide variants and presence of insertion/deletion variants, and subsequently used the aligned short read contig sequences to remove false nucleotide calls. We then align the entire genome against itself using BLASTN to check the integrity of the corrected genome sequence. For completeness, we have have provided raw LR-chr sequence, frame-shift-corrected sequence and, for the subset of genomes subjected to further refinement, the fully completed versions.

### Identification and analysis of non-chromosomal replicons and short microbial chromosomes

To screen for the presence of plasmid and phage genomes, we examined all circular (based on ’suggestCircular=yes’ in the header from canu assembly) LRAC sequences less than 1 Mbp in length. We compared these LRAC sequences to check which of them were similar and had high Average Nucleotide identity (ANI) using the dRep (version 2.2.3) ’compare’ workflow with default parameters (except -p 10 -d --S_algorithm ANIn). We predicted open reading frames and annotated them using Prokka (version 1.13) with default settings (except --metagenome --addmrna --addgenes --cpu 5) and screened for the presence of KEGG Ortholog genes by mapping the predicted proteins to KEGG prokaryotic gene database (2017 release) using blastp in diamond (version v0.9.18). We performed taxonomic annotation using GTDB-Tk (version 0.3.2) ’classify_wf’ workflow running default parameters except --cpus 40 -x fasta for PAO4-tig00000333. We used CheckV (version 0.6.0)^63^ to identify viruses and to estimate their sequence quality using the ’end_to_end’ workflow with default parameters (except -t 20 -d checkv-db-v0.6). We also mapped the LRAC sequences to databases of plasmids (ftp://ftp.ncbi.nlm.nih.gov/refseq/release/plasmid/—downloaded on April 30, 2020) and viruses (ftp://ftp.ncbi.nlm.nih.gov/refseq/release/viral—downloaded on May 18, 2020 and IMG/VR downloaded on June 9, 2020) using BLASTN (from ncbi-blast-2.7.1+, running default parameters except -outfmt 6 with the addition of qlen slen qcovs qcovhsp qcovus) to check for alignment to known plasmids and viruses. Results from the ’qcovs’ format specifier in BLASTN was used to estimate the Query Coverage Per Subject.

### Hybrid metagenome assembly workflows

We performed hybrid metagenome assemblies on long and short read data using hybridSPAdes^65^ and OPERA-MS^18^. SPAdes (version 3.14.1-Linux) was run with default settings except -k 21,33,55,77,99,127 --meta. Paired short reads were provided with -1, -2 options and long reads were provided with --nanopore option. OPERA-MS (version 0.9.0) was run with default settings except --num-processors 44 and short reads already assembled using SPAdes were employed. The long reads were provided with the --long-read option. Contigs from hybrid assemblies at least 500 bp in length were denoted as hybrid assembled contigs (HYAC). The number of short and long reads used in each hybrid assembly was estimated by mapping short and long reads separately to HYAC sequences with minimap2 (version 2.17) and using samtools-1.10 to calculate the number of aligned reads. HYAC sequence that was at least 1 Mbp in length were categorised as potential whole-chromosome sequence and denoted as HY-chr sequence. HY-chr sequences were processed with CheckM (version 1.1.3) to assess genome quality. HY-chr sequences that demonstrated CheckM-SCG completeness >90% and contamination <5% were classified as putative genomes. We performed taxonomic annotation on recovered genome sequence using GTDB-Tk (version v1.3.0 and GTDB-Tk reference data version r95, running default parameters except --cpus 40 -x fasta). Coverage profiles were generated from both long read and short read data, by mapping each of these to HY-chr sequences with minimap2 (version 2.17) using the following flags -ax map-ont -a -t 40 for long read data and -ax sr -a -t 40 for short read data. Sorted .bam files were subsequently processed using bedtools genomeCoverageBed(version 2.26.0) with the following flag -d.

### Reporting summary

Further information on research design is available in the Nature Research Reporting Summary linked to this article.

## Supplementary information

Supplementary Information

Supplementary Data 1

Supplementary Data 2

Supplementary Data 3

Supplementary Data 4

Supplementary Data 5

Supplementary Data 6

Supplementary Data 7

Supplementary Data 8

Supplementary Data 9

Supplementary Data 10

Reporting Summary

## Data Availability

Raw sequence data from long and short read sequencing are available at NCBI via the following BioProject accessions: *PAO1*: PRJNA509764; *PAO2*: PRJNA611629; *PAO3A and PAO3B*: PRJNA606905 and *PAO4*: PRJNA607349. The chromosomal sequences are available at NCBI via the Genbank accession numbers listed in Table 1. Data products from this study are contained in two Zenodo submissions (one made before initial submission, the second following review): the first submission (10.5281/zenodo.3695987) includes: (1) LRAC sequence from each dataset; (2) whole-genome sequence (FASTA), genome quality statistics (CheckM) and genome annotation data (Prokka) from 21/22 genomes listed in Table 1 for each of the five correction procedures; (3) short read assembled sequence and binning results; (4) concordance statistic data and results; (5) short and long read per-base coverage data for 21/22 genomes and (5) two manually corrected genomes of *Candidatus* Accumulibacter along with detailed notes explaining the procedures that were applied. The second Zenodo submission (10.5281/zenodo.4317309) contains equivalent data for the Contendobacter genome and outputs from both hybrid metagenome workflows.

## Code availability

The R code for performing the concordance statistic analysis are available at https://github.com/rbhwilliams/srac2lrac including test data and scripts taken from the PAO2 data.
