# Supplementary material for: Recovery of complete genomes and non-chromosomal replicons from activated sludge enrichment microbial communities with long read metagenome sequencing
Source: NPJ Biofilms Microbiomes. 2021 Mar 16;7:23. doi: 10.1038/s41522-021-00196-6 (PMC7966762; doi:10.1038/s41522-021-00196-6)
Supplement: Supplementary file 1 — Supplementary Information [file 41522_2021_196_MOESM1_ESM.pdf]

Supplementary Material for *Recovery of complete genomes and non-chromosomal replicons from activated sludge enrichment microbial communities with long read metagenome sequencing*, Arumugam, Bessarab *et al.*

Table S1: Summary statistics of long read data by dataset

Summary measure	PAO1	PAO2	PAO3A	PAO3B	PAO4
Date of run	06/06/2018	09-10/04/2018	06/08/2018	07/08/2018	02/10/2019
Basecaller	ont-guppy-cpu_3.2.2_linux64	ont-guppy-cpu_3.2.2_linux64	ont-guppy-cpu_3.2.1_linux64	ont-guppy-cpu_3.2.1_linux64	ont-guppy-cpu_3.3.0_linux64
#Reads after basecalling	695,231	88,285	599,701	795,231	1,683,426
#Reads after adapter trimming	695,348	87,440	599,675	795,220	1,684,574
Total size of Reads (bp)	6,352,608,600	1,148,777,355	5,714,650,294	6,824,737,520	11,912,102,097
Longest Read (bp)	67,768	140,657	926,504	629,419	285,399
Shortest Read (bp)	1	1	1	1	1
#Reads>500	690,938	76,062	586,906	778,288	1,622,061
#Reads>1000	680,417	71,795	576,813	757,762	1,537,598
#Reads>10k	246,720	38,506	239,434	248,600	371,326
#Reads>100k	0	22	34	24	4
#Reads>1M	0	0	0	0	0
Mean (bp)	9,136	13,138	9,530	8,582	7,071
N50 (bp)	9,988	27,030	10,658	9,849	9,200
NCBI BioProject	PRJNA509764	PRJNA611629	PRJNA606905	PRJNA606905	PRJNA607349

Table S2: Summary statistics for long read assemblies from three assembly workflows categorised by data set

Workflow	PAO1			PAO2			PAO3A			PAO3B			PAO4		
	Canu_v1.8	Unicycler_v0.4.7	Flye_v2.4	Canu_v1.8	Unicycler_v0.4.7	Flye_v2.4	Canu_v1.8	Unicycler_v0.4.7	Flye_v2.4	Canu_v1.8	Unicycler_v0.4.7	Flye_v2.4	Canu_v1.9	Unicycler_v0.4.8	Flye_v2.4
#Input Reads	695,348	695,348	695,348	87,440	87,440	87,440	599,675	599,675	599,675	795,220	795,220	795,220	1,684,574	1,684,574	1,684,574
#Contigs	7,298	1,745	2,653	677	143	258	5,288	1,407	2,417	7,109	2,168	3,314	5,778	1,215	1,391
Total size of Contigs (bp)	238,017,517	106,910,637	178,268,711	49,846,700	32,750,654	51,446,988	215,029,381	115,565,249	183,865,684	293,784,509	177,451,963	246,070,543	150,772,785	96,963,001	115,165,901
Longest Contig (bp)	5,182,841	5,188,404	5,188,470	5,014,776	5,084,209	4,316,856	7,641,424	4,696,726	4,701,758	7,092,454	4,703,771	5,217,752	6,232,277	4,240,027	4,640,521
Shortest Contig (bp)	1,054	809	712	1,055	3,655	626	1,038	1,524	560	1,017	894	528	996	862	733
#Contigs>500	7,298	1,745	2,653	677	143	258	5,288	1,407	2,417	7,109	2,168	3,314	5,778	1,215	1,391
#Contigs>1000	7,298	1,744	2,652	677	143	256	5,288	1,407	2,414	7,109	2,167	3,309	5,777	1,213	1,388
#Contigs>10k	4,011	1,717	2,533	436	138	233	3,391	1,392	2,282	4,329	2,149	3,080	2,233	1,187	1,311
#Contigs>100k	351	142	286	58	78	123	303	186	331	413	374	447	134	206	155
#Contigs>1M	14	10	7	9	3	7	20	14	12	33	12	20	14	5	14
Mean (bp)	32,614	61,267	67,195	73,629	229,026	199,407	40,664	82,136	76,072	41,326	81,851	74,252	26,094	79,805	82,794
N50 (bp)	96,302	104,314	83,753	729,548	368,048	496,681	121,512	183,372	107,425	173,626	163,443	119,307	173,745	158,520	199,280
WallClock Time (seconds)	75,705	19,368	9,089	376,437	5,091	1,711	44,650	18,971	7,250	39,011	22,258	8,105	243,508	35,707	12,362
#Cores	NA	20	20	NA	20	20	NA	20	20	NA	20	20	NA	44	20
Maxvmem (GB)	155.90	78.09	155.90	109.22	18.81	64.39	414.10	70.15	143.04	299.26	73.54	168.13	303.85	357.08	286.26
#Reads Used in assembly*	577,472	524,026	548,397	56,721	51,436	56,014	514,118	484,409	501,217	687,039	650,051	667,262	1,489,180	1,451,835	1,466,912

*Obtained by mapping nanopore reads to LRAC using minimap2 (see Materials and Methods)

For Canu, default setting of genomeSize parameter was 100Mbp, except PAO2 for which is was set to 4Mbp. For Flye the genome-size parameter was always set to 100Mbp

NA - 'useGrid=false' option was used

Table S3: CheckM summary statistics of LR-chr from three assembly workflows categoried by data set

	PAO1			PAO2			PAO3A			PAO3B			PAO4		
	**Canu**	**Flye**	**Unicycler**	**Canu**	**Flye**	**Unicycler**	**Canu**	**Flye**	**Unicycler**	**Canu**	**Flye**	**Unicycler**	**Canu**	**Flye**	**Unicycler**
**Number of sequences**															
**#Contigs>1MB**	14	7	10	9	7	3	20	12	14	33	20	12	14	14	5
**#Contigs>1MB with MEGAN-LR Frameshift correction**	14	7	10	9	7	3	20	11	12	33	20	12	14	14	5
**#High quality MAGs (Completeness >90 and contamination <5)**	7	3	5	2	1	2	6	2	2	4	6	4	5	3	2
**CheckM Completeness (%)**															
**Mean**	65.94	88.54	69.83	56.99	48.36	91.87	54.84	61.90	48.97	52.69	50.18	61.34	70.16	57.70	57.88
**Min**	4.17	76.35	20.07	13.79	15.52	89.34	0.00	14.43	12.07	15.52	0.00	8.33	23.96	31.16	25.80
**Max**	97.53	93.59	98.09	94.49	92.18	95.80	99.04	95.32	96.55	98.10	96.93	98.90	99.14	94.06	96.34
**CheckM Contamination (%)**															
**Mean**	0.63	0.28	1.07	0.46	0.67	0.36	4.18	0.46	0.97	2.42	0.76	0.54	12.76	0.95	0.38
**Min**	0.00	0.00	0.00	0.00	0.00	0.00	0.00	0.00	0.00	0.00	0.00	0.00	0.00	0.00	0.00
**Max**	1.98	1.11	6.74	2.27	2.00	0.66	75.22	2.73	7.52	56.97	10.18	2.73	78.79	8.62	0.98

Table S4: Number of reads used in generating recovered *genomes* categorised by data set

Genome	#Input Reads	#Reads used in assembly	#LR mapped to the genome[b]	#LR mapped to genome after subsetting from LR2contigs .bam[c]	%From mapping to genome	%From subsetting
PAO1-tig00000001	695,348	577,472	255,609	220,448	44.3	38.2
PAO1-tig00000003	695,348	577,472	12,166	7,098	2.1	1.2
PAO1-tig00000117	695,348	577,472	11,101	7,580	1.9	1.3
PAO1-tig00026549	695,348	577,472	41,751	15,911	7.2	2.8
PAO1-tig00026557	695,348	577,472	28,521	16,827	4.9	2.9
PAO1-tig00026560	695,348	577,472	32,268	28,480	5.6	4.9
PAO1-tig00198536	695,348	577,472	27,267	23,563	4.7	4.1
**Total**	**695,348**	**577,472**	**408,683**	**319,907**	**70.8**	**55.4**
PAO2-tig00000001	87,440	56,721	20,997	14,580	37.0	25.7
PAO2-tig00000013	87,440	56,721	15,634	7,251	27.6	12.8
**Total**	**87,440**	**56,721**	**36,631**	**21,831**	**64.6**	**38.5**
PAO3A-tig00000003	599,675	514,118	95,781	49,716	18.6	9.7
PAO3A-tig00000011	599,675	514,118	68,997	32,592	13.4	6.3
PAO3A-tig00000024	599,675	514,118	19,643	5,090	3.8	1.0
PAO3A-tig00000209	599,675	514,118	52,303	16,186	10.2	3.1
PAO3A-tig00018026	599,675	514,118	125,187	106,395	24.3	20.7
PAO3A-tig00139797	599,675	514,118	52,917	20,773	10.3	4.0
**Total**	**599,675**	**514,118**	**414,828**	**230,752**	**80.7**	**44.9**
**PAO3B-tig00000003[a]**	**795,220**	**687,039**	**129,258**	**105,890**	**18.8**	**15.4**
PAO3B-tig00000024	795,220	687,039	63,688	17,214	9.3	2.5
PAO3B-tig00000027	795,220	687,039	47,875	12,108	7.0	1.8
**PAO3B-tig00157979[a]**	**795,220**	**687,039**	**60,017**	**24,862**	**8.7**	**3.6**
**Total**	**795,220**	**687,039**	**300,838**	**160,074**	**43.8**	**23.3**
PAO4-tig00000001	1,684,574	1,489,180	256,088	43,078	17.2	2.9
PAO4-tig00000030	1,684,574	1,489,180	143,439	59,942	9.6	4.0
PAO4-tig00000046	1,684,574	1,489,180	709,008	257,789	47.6	17.3
PAO4-tig00000079	1,684,574	1,489,180	24,278	15,976	1.6	1.1
PAO4-tig00000228	1,684,574	1,489,180	104,705	12,409	7.0	0.8
**Total**	**1,684,574**	**1,489,180**	**1,237,518**	**389,194**	**83.1**	**26.1**

[a] Redundant genomes included here to correctly estimate proportion of reads generating genomes in PAO3B data
[b] All long reads from the corresponding dataset mapped to the each genome. *#LR*: number of long reads
[c] Subset of mapped reads obtained from .bam file mapping all long reads to all LRAC generated from the assembly.

Table S5: Summary statistics for short read assemblies categorised by data set

Assembly Statistics	PAO1[a]	PAO2[a]	PAO3A, PAO3B Coassembly[a]	PAO4[b]
#Input Reads	43,856,872	47,972,460	312,124,162	47,173,360
#Contigs	539,404	259,279	905,357	224,641
Total size of Contigs (bp)	687,037,129	432,809,862	1,584,814,703	326,649,599
Longest Contig (bp)	2,684,500	1,730,458	1,530,474	2,088,122
Shortest Contig (bp)	500	500	500	500
#Contigs>500	536,933	258,454	902,774	223,983
#Contigs>1000	156,620	75,955	317,494	69,187
#Contigs>10k	4,267	4,153	16,811	1,982
#Contigs>100k	175	302	771	152
#Contigs>1M	1	3	4	1
Mean (bp)	1,274	1,669	1,750	1,454
N50 (bp)	1,482	3,142	3,061	1,906
WallClock Time (seconds)	48,968	31,535	170,876	22,110
#Cores	24	30	40	44
Maxvmem (GB)	80.20	58.19	184.02	95.86
#Reads Used in assembly*	39,841,222	45,634,652	304,067,129	45,041,741

[a] Constructed SRAC using SPAdes-3.12.0

[b] Constructed SRAC using SPAdes-3.14.0

*mapped Short Reads to SRAC using bowtie2

Table S6: Summary statistics for hybrid metagenome assemblies from two assembly workflows categoried by data set

Dataset	PAO1		PAO2		PAO3A		PAO3B		PAO4	
Workflow	OPERA-MS-v0.9.0	SPAdes_v3.14.1	OPERA-MS-v0.9.0	SPAdes_v3.14.1	OPERA-MS-v0.9.0	SPAdes_v3.14.1	OPERA-MS-v0.9.0	SPAdes_v3.14.1	OPERA-MS-v0.9.0	SPAdes_v3.14.1
#Input Reads - Long Reads	695,348	695,348	87,440	87,440	599,675	599,675	795,220	795,220	1,684,574	1,684,574
#Input Reads - Short Reads	43,856,872	43,856,872	47,972,460	47,972,460	312,124,162	312,124,162	312,124,162	312,124,162	47,173,360	47,173,360
#Contigs	454,419	455,487	251,940	250,105	881,507	877,437	867,644	864,163	161,128	160,127
Total size of Contigs (bp)	739,541,050	727,338,430	446,457,681	435,600,413	1,600,878,232	1,594,101,557	1,611,216,935	1,599,717,183	361,380,261	365,472,408
Longest Contig (bp)	3,013,135	2,681,211	3,551,406	2,990,671	3,789,289	3,050,167	2,107,369	3,391,433	3,009,781	4,072,768
Shortest Contig (bp)	500	500	500	500	500	500	500	500	500	500
#Contigs>500	452,062	453,281	251,121	249,303	878,938	874,891	865,089	861,627	160,555	159,606
#Contigs>1000	113,795	139,394	70,868	70,050	301,109	298,811	292,761	293,474	39,763	49,857
#Contigs>10k	9,254	6,363	3,868	3,731	15,192	16,056	15,865	16,721	4,574	5,237
#Contigs>100k	343	237	368	116	974	926	1,030	930	288	146
#Contigs>1M	10	7	6	9	19	20	24	28	13	14
Mean (bp)	1,627	1,597	1,772	1,742	1,816	1,817	1,857	1,851	2,243	2,282
N50 (bp)	3,812	2,977	4,135	3,844	3,511	3,561	3,932	3,911	15,147	8,021
WallClock Time (seconds)	85,208	104,906	52,150	41,792	265,622	306,870	265,505	194,405	23,910	65,852
#Cores	44	25	44	25	44	25	44	44	44	25
Maxvmem (GB)	113.88	189.72	42.00	112.61	114.83	406.75	115.13	407.73	78.07	79.20
#Long Reads Used in assembly*	635,411	635,443	67,199	67,224	548,200	548,332	733,278	733,597	1,523,052	1,523,747
#Short Reads Used in assembly**	40,734,601	40,811,485	46,203,792	46,213,027	307,682,499	308,274,531	307,786,095	308,271,664	45,530,389	45,585,411

*Obtained by mapping long reads to HYAC using minimap2 (see Materials and Methods)

**Obtained by mapping short reads to HYAC using minimap2 and calculated number of reads from samtools flagstat (see Materials and Methods)

Note: OPERA-MS-v0.9.0 Wall Clock time for PAO3A and PAO3B excludes pilon polishing stage

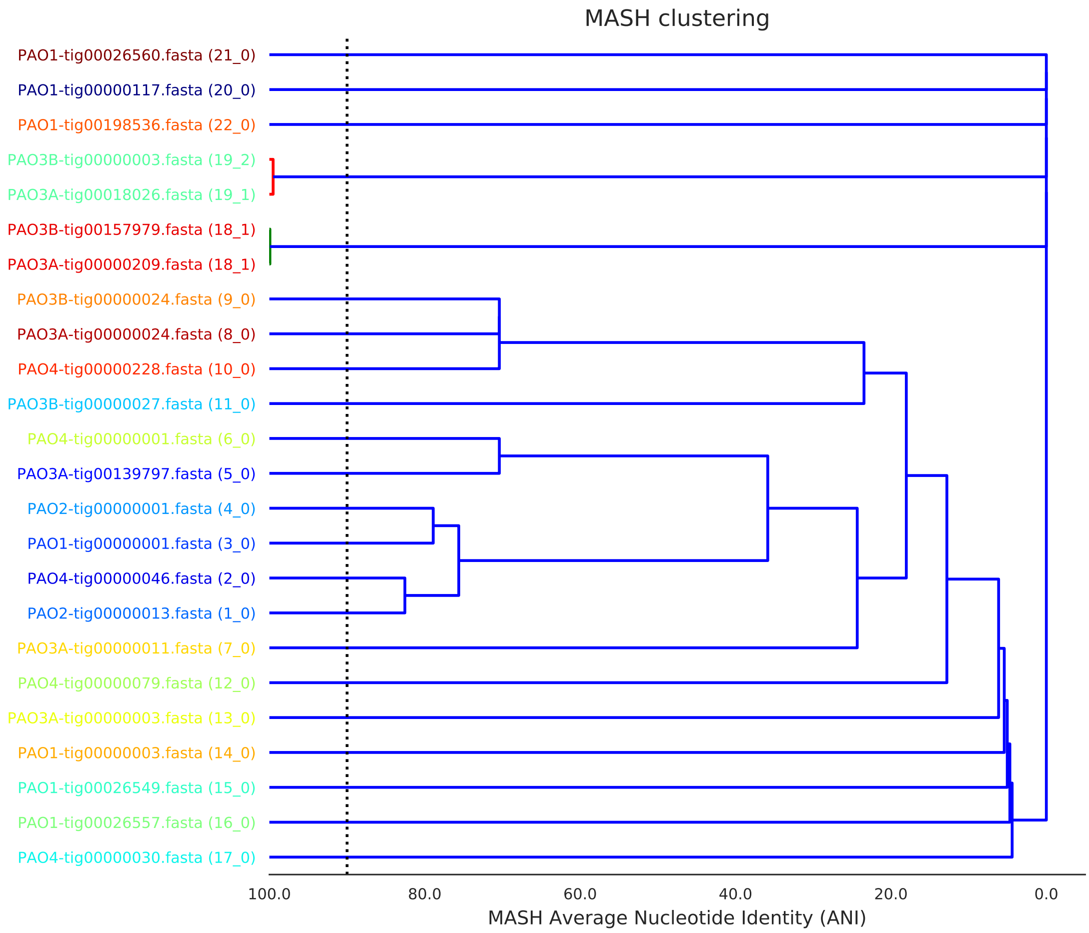

Supplementary Figure 1: Dendrogram generated from MASH analysis (`dRep`) of 24 putative genomes recovered in this study.

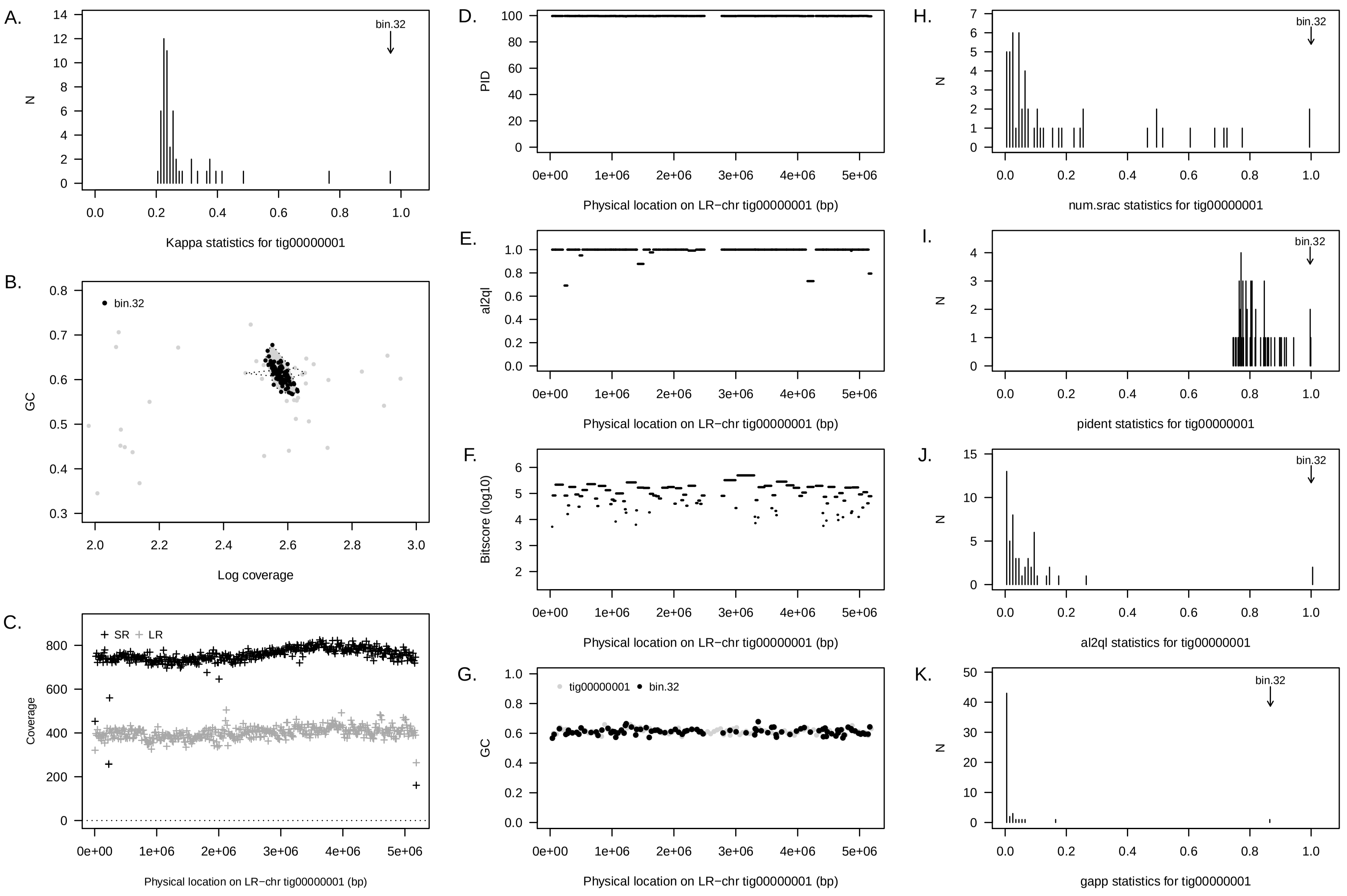

Supplementary Figure 2: Summary of concordance statistic analysis for an LR–chr (`tig00000001`) from the PAO1 reactor community (annotated to *Candidatus* Accumulibacter) and a short read metagenome assembled genome from the same reactor community (`bin 32`). See Figure 1 for interpretation guide.

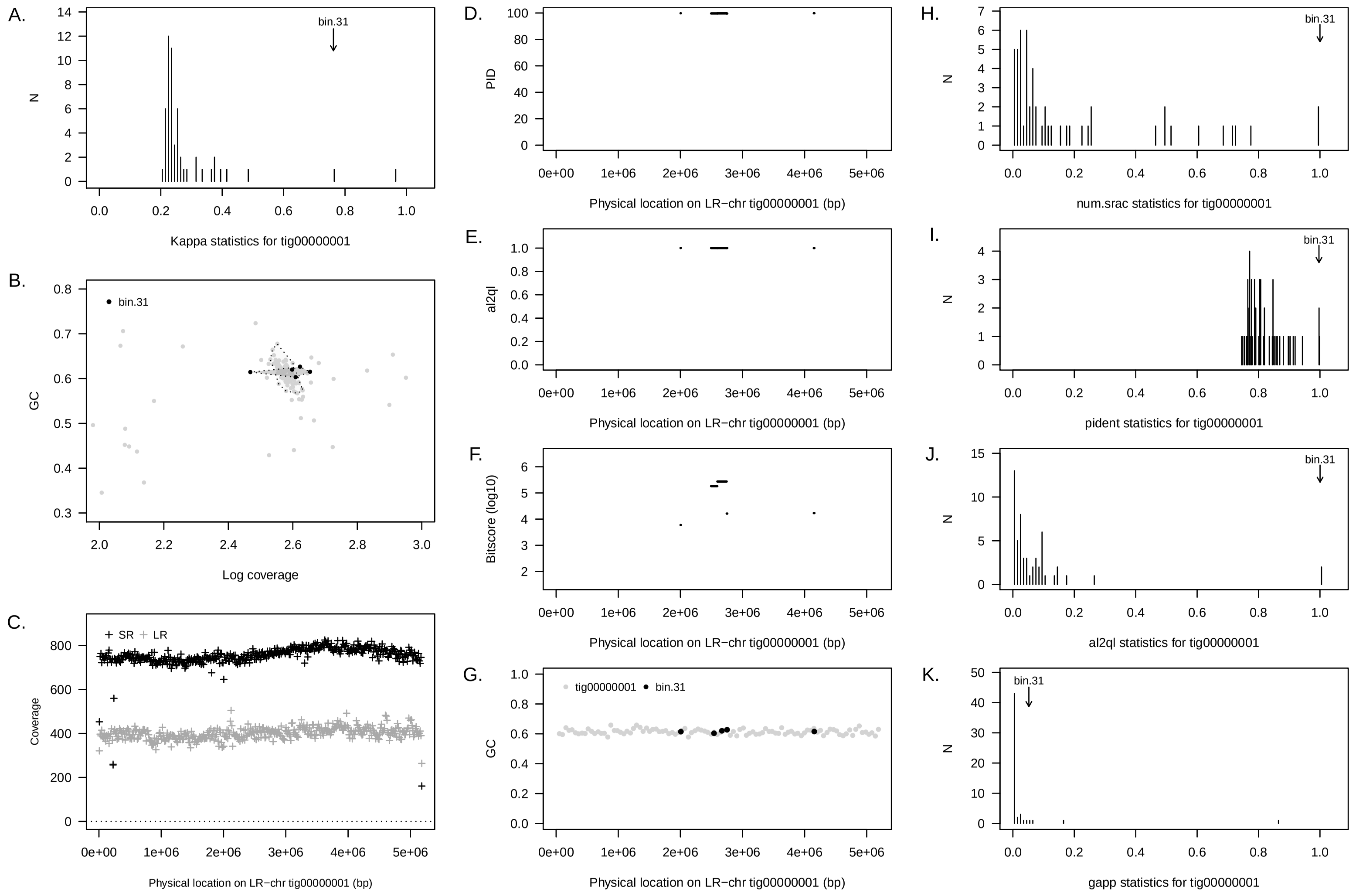

Supplementary Figure 3: Summary of concordance statistic analysis for an LR–chr (`tig00000001`) from the PAO1 reactor community (annotated to *Candidatus* Accumulibacter) and a short read metagenome assembled genome from the same reactor community (`bin 31`). See Figure 1 for interpretation guide. In this case, it appears as if (short read) bin 31 is split from the main bin 32 that provides the largest κ score. Note that bin 31 fills the alignment gap observed from bin 32.

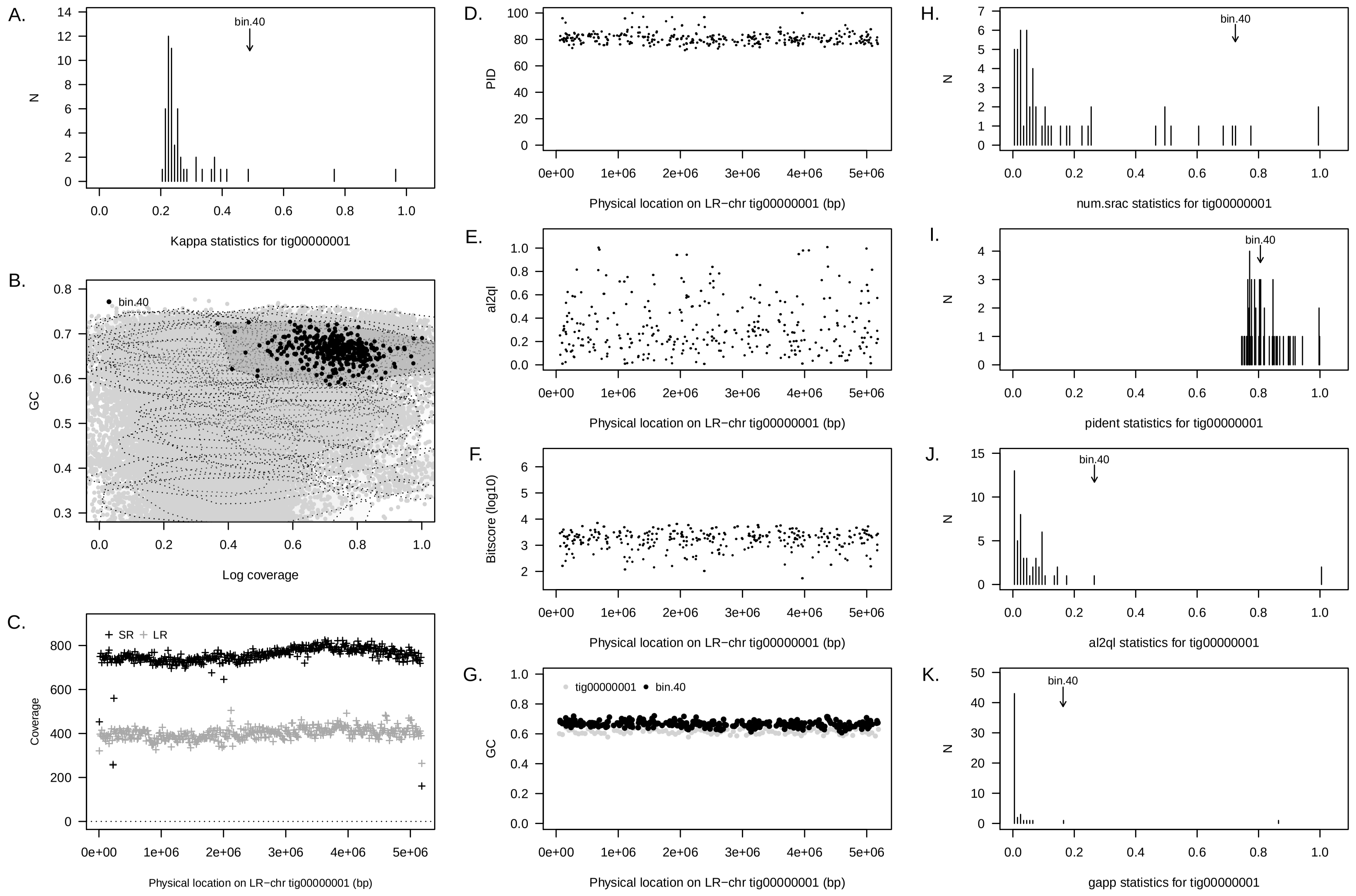

Supplementary Figure 4: Summary of concordance statistic analysis for an LR–chr (`tig00000001`) from the PAO1 reactor community (annotated to *Candidatus* Accumulibacter) and a short read metagenome assembled genome from the same reactor community (`bin 40`). See Figure 1 for interpretation guide. In this case, we show the results for a lower abundance short read bin annotated to *Candidatus* Accumulibacter.

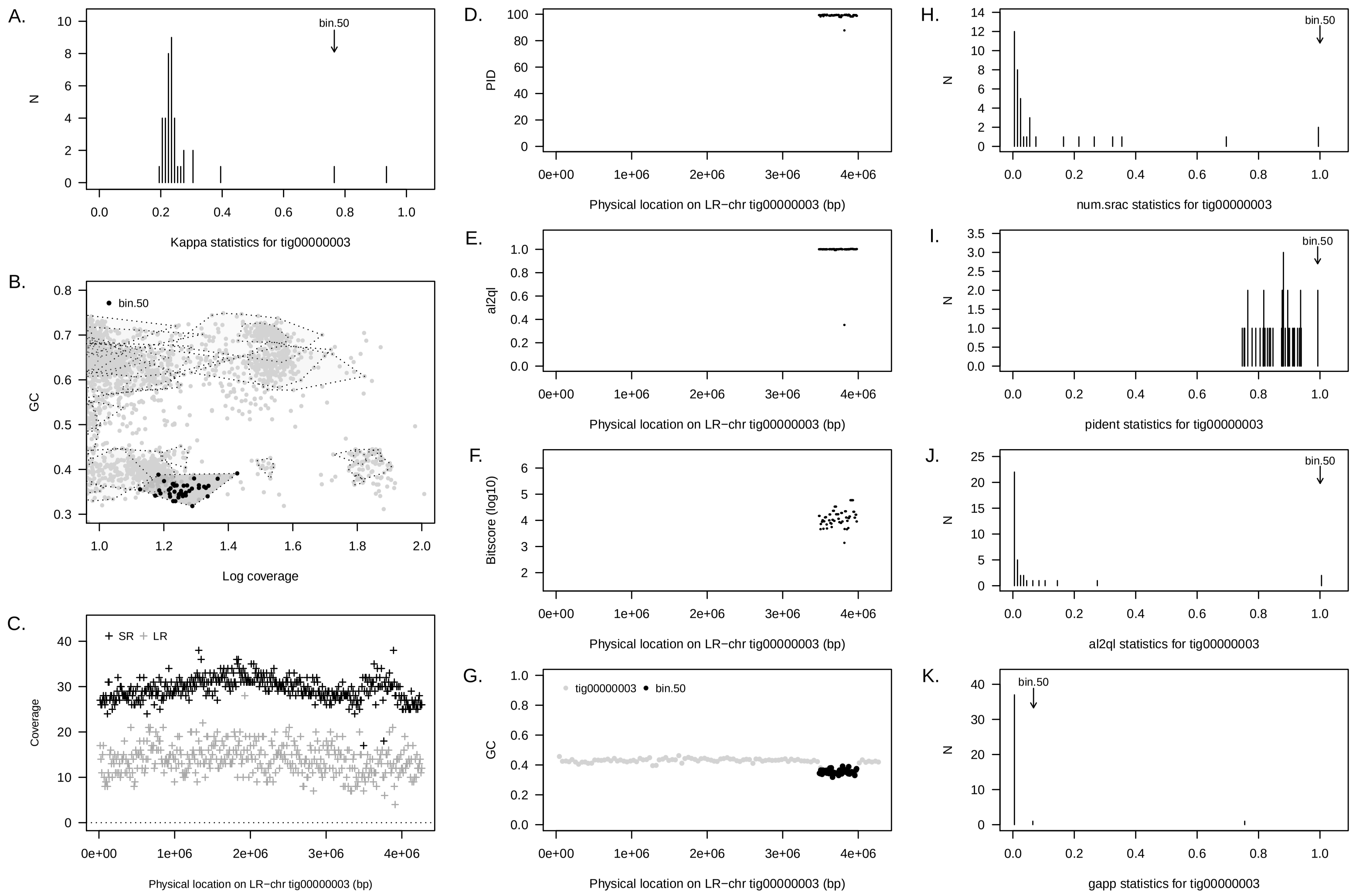

Supplementary Figure 5: Summary of concordance statistic analysis for an LR–chr (`tig00000003`) from the PAO1 reactor community, annotated to a member of genus OLB11 (family: Chitinophagaceae), and a short read metagenome assembled genome from the same reactor community (`bin 50`). See Figure 1 for interpretation guide. As in the case of PAO1-tig00000001, it appears as if multiple short bins, artefactually split by the Metabat2, represent the cognates of this long read genome (see next figure)

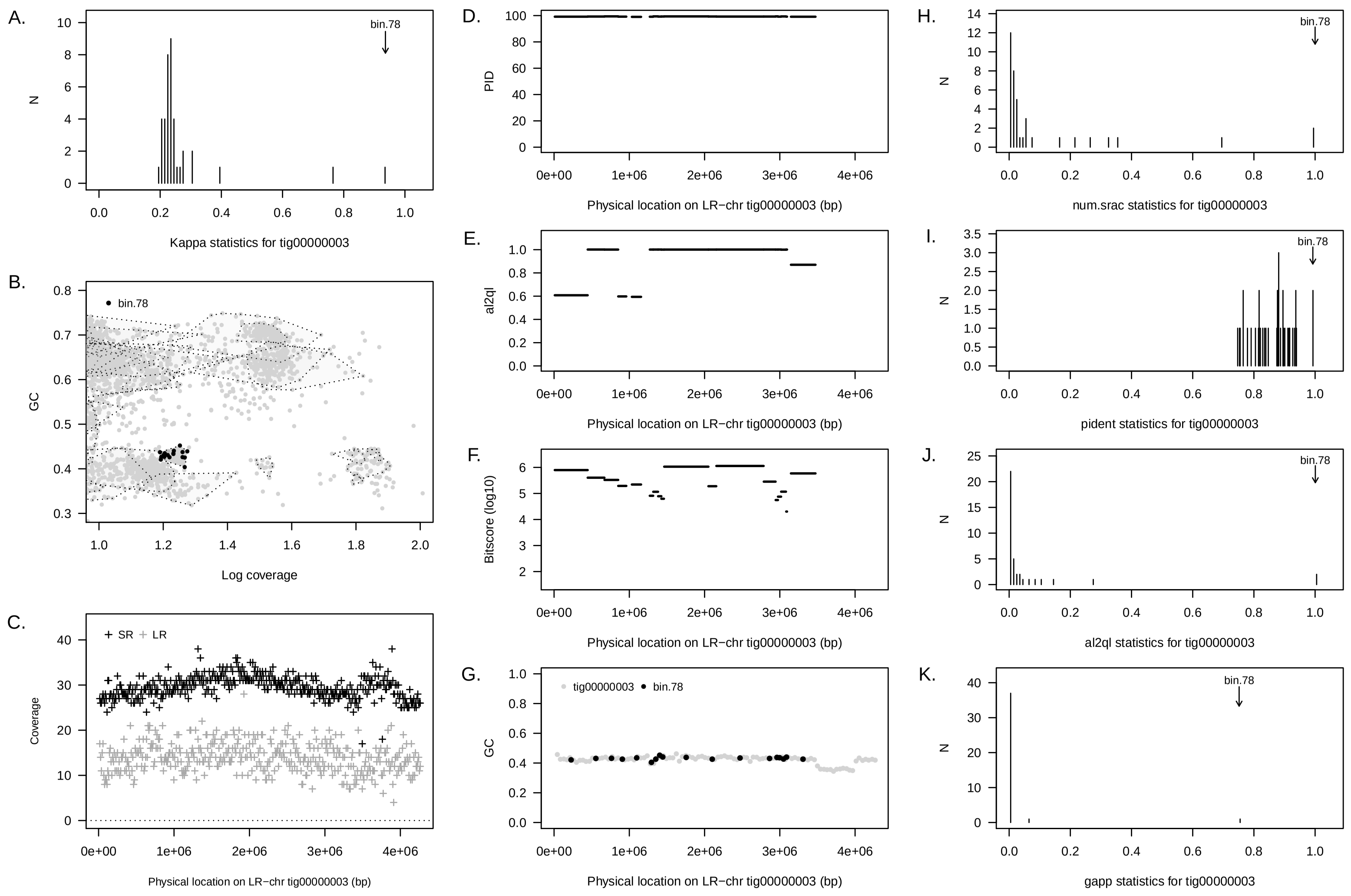

Supplementary Figure 6: Summary of concordance statistic analysis for an LR–chr (`tig00000003`) from the PAO1 reactor community, annotated to a member of genus OLB11 (family: Chitinophagaceae), and a short read metagenome assembled genome from the same reactor community (`bin 78`). See Figure 1 for interpretation guide.

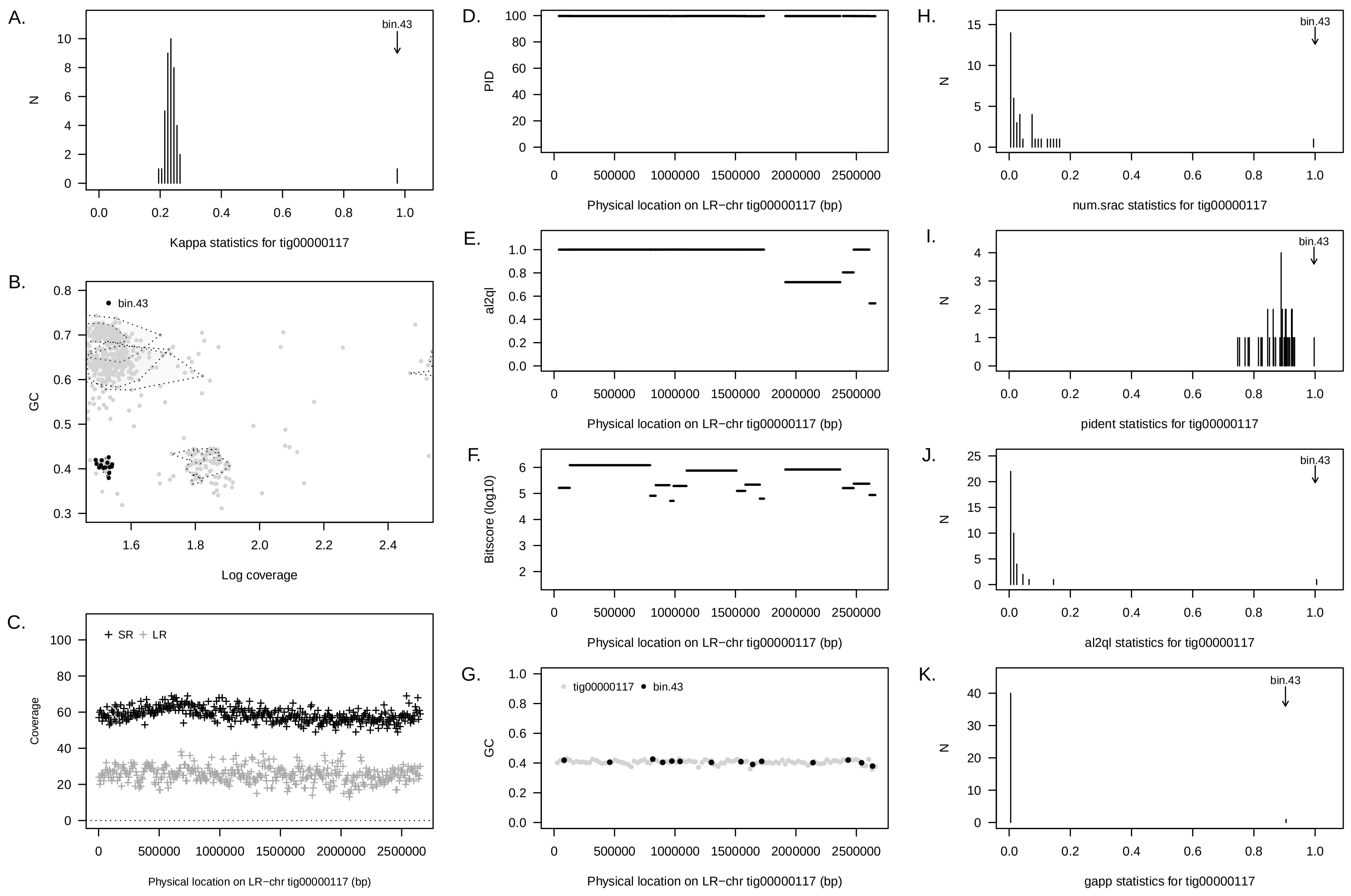

Supplementary Figure 7: Summary of concordance statistic analysis for an LR–chr (`tig00000117`) from the PAO1 reactor community, annotated to a member of genus UBA6002 (class: Gammaproteobacteria), and a short read metagenome assembled genome from the same reactor community (`bin 43`). See Figure 1 for interpretation guide.

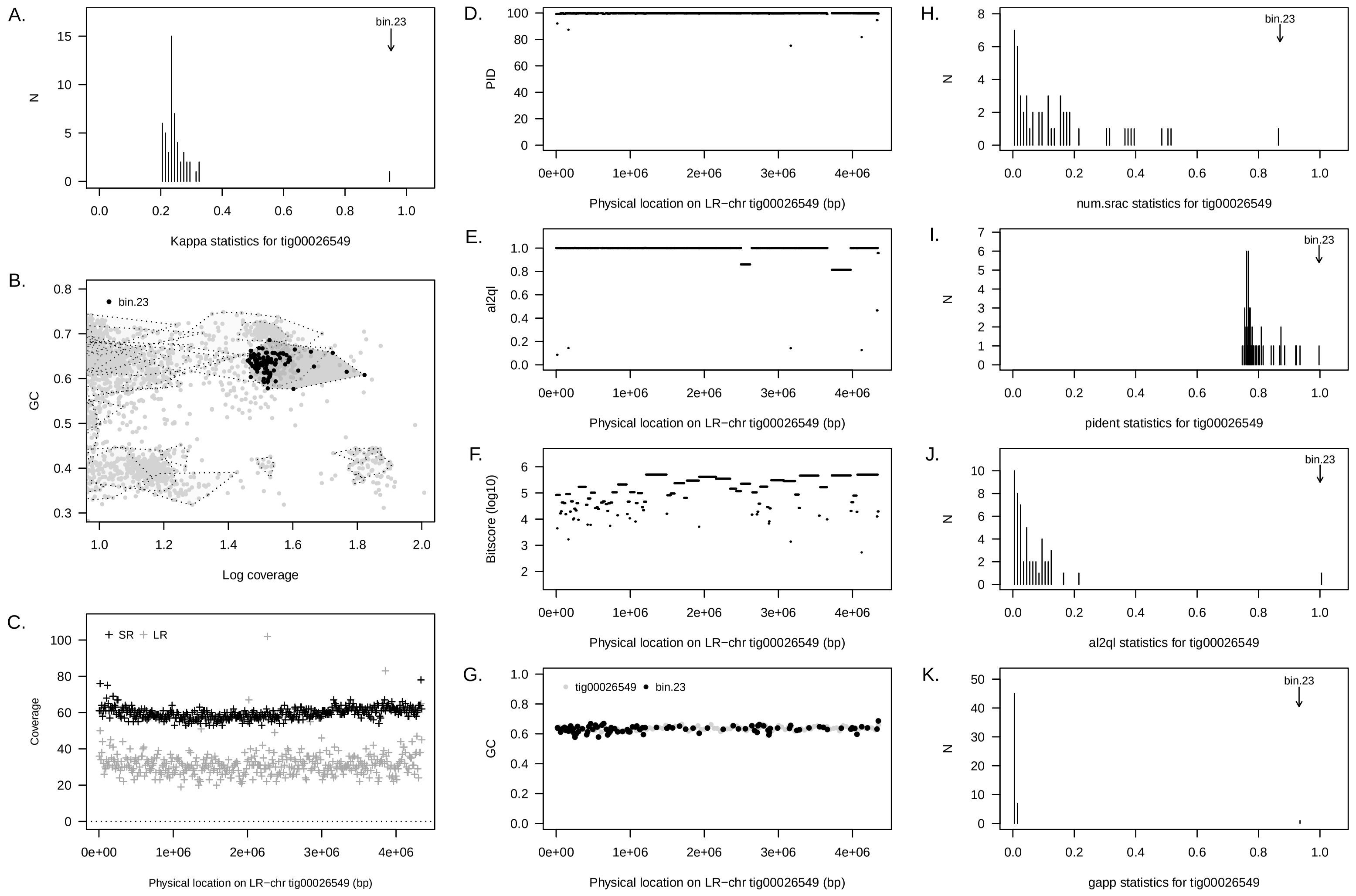

Supplementary Figure 8: Summary of concordance statistic analysis for an LR–chr (`tig00026549`) from the PAO1 reactor community and a short read metagenome assembled genome from the same reactor community (`bin 43`). See Figure 1 for interpretation guide. This genome is likely to be that of a member of genus *Defluviicoccus* known to exhibit the glycogen accumulating organism (GAO) phenotype.

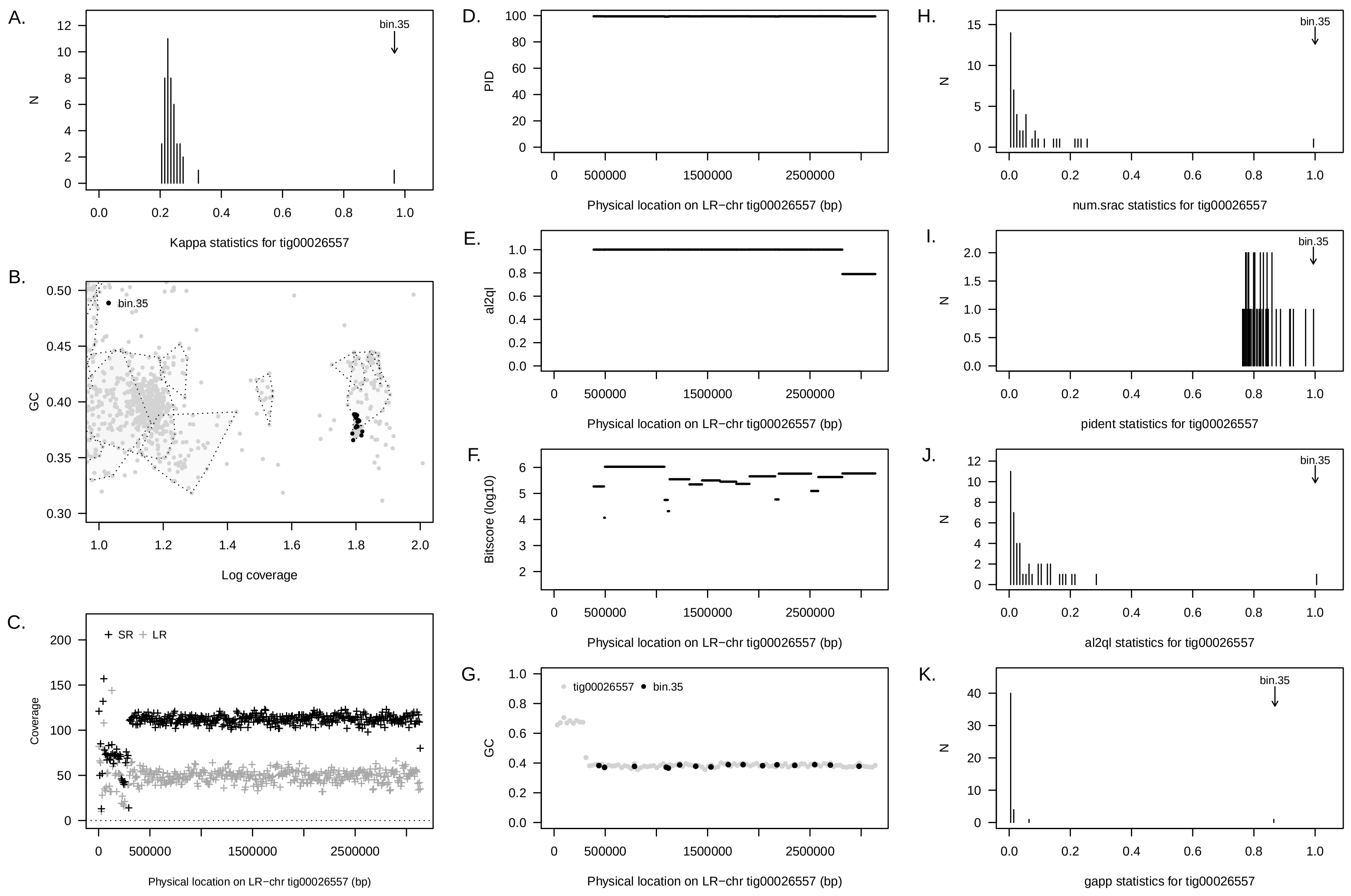

Supplementary Figure 9: Summary of concordance statistic analysis for an LR–chr (`tig00026557`) from the PAO1 reactor community, annotated to family *Parachlamydiae* and a short read metagenome assembled genome from the same reactor community (`bin 35`). See Figure 1 for interpretation guide.

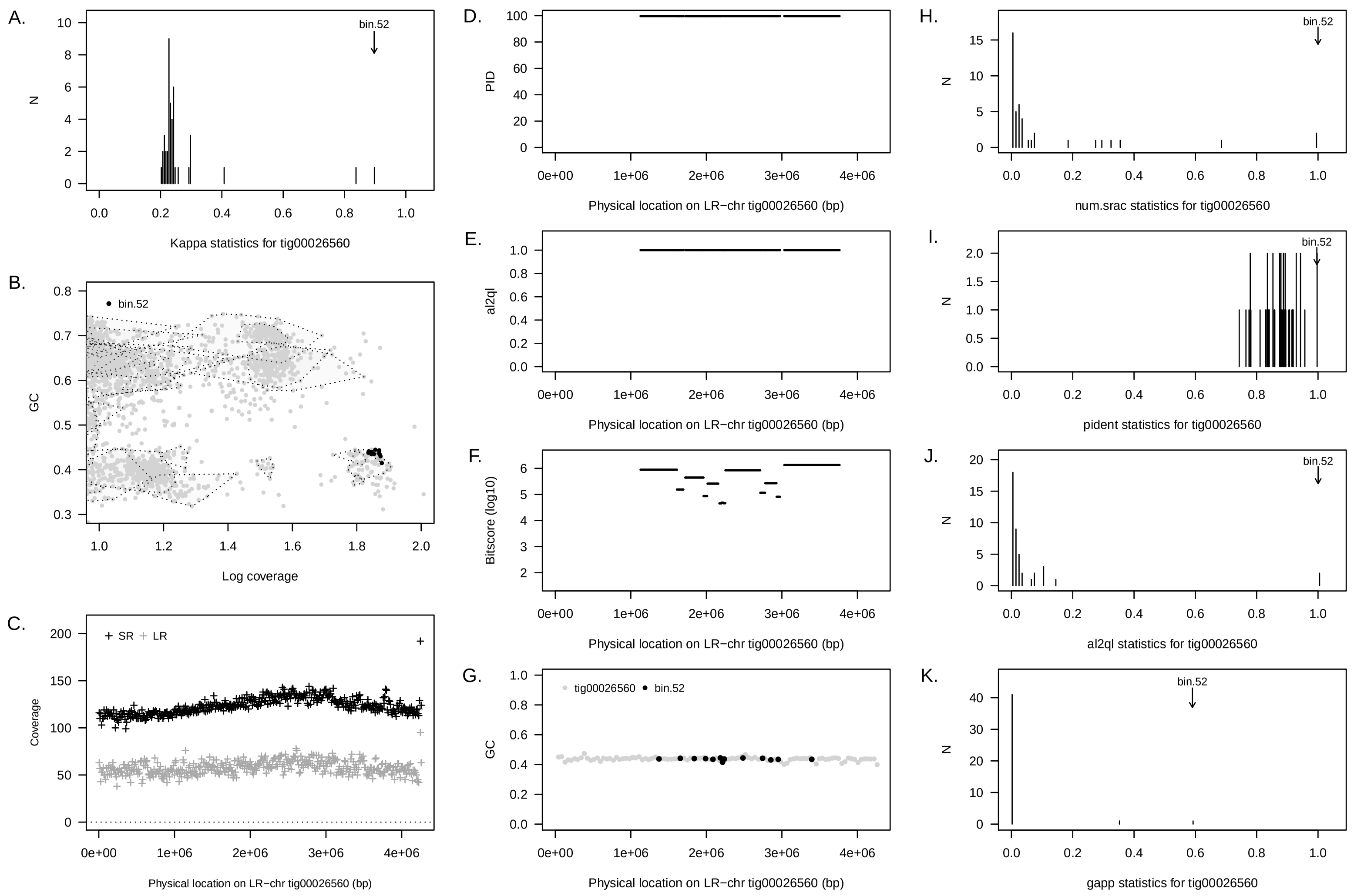

Supplementary Figure 10: Summary of concordance statistic analysis for an LR–chr (`tig00026560`) from the PAO1 reactor and a short read metagenome assembled genome from the same reactor community (`bin 52`). See Figure 1 for interpretation guide.

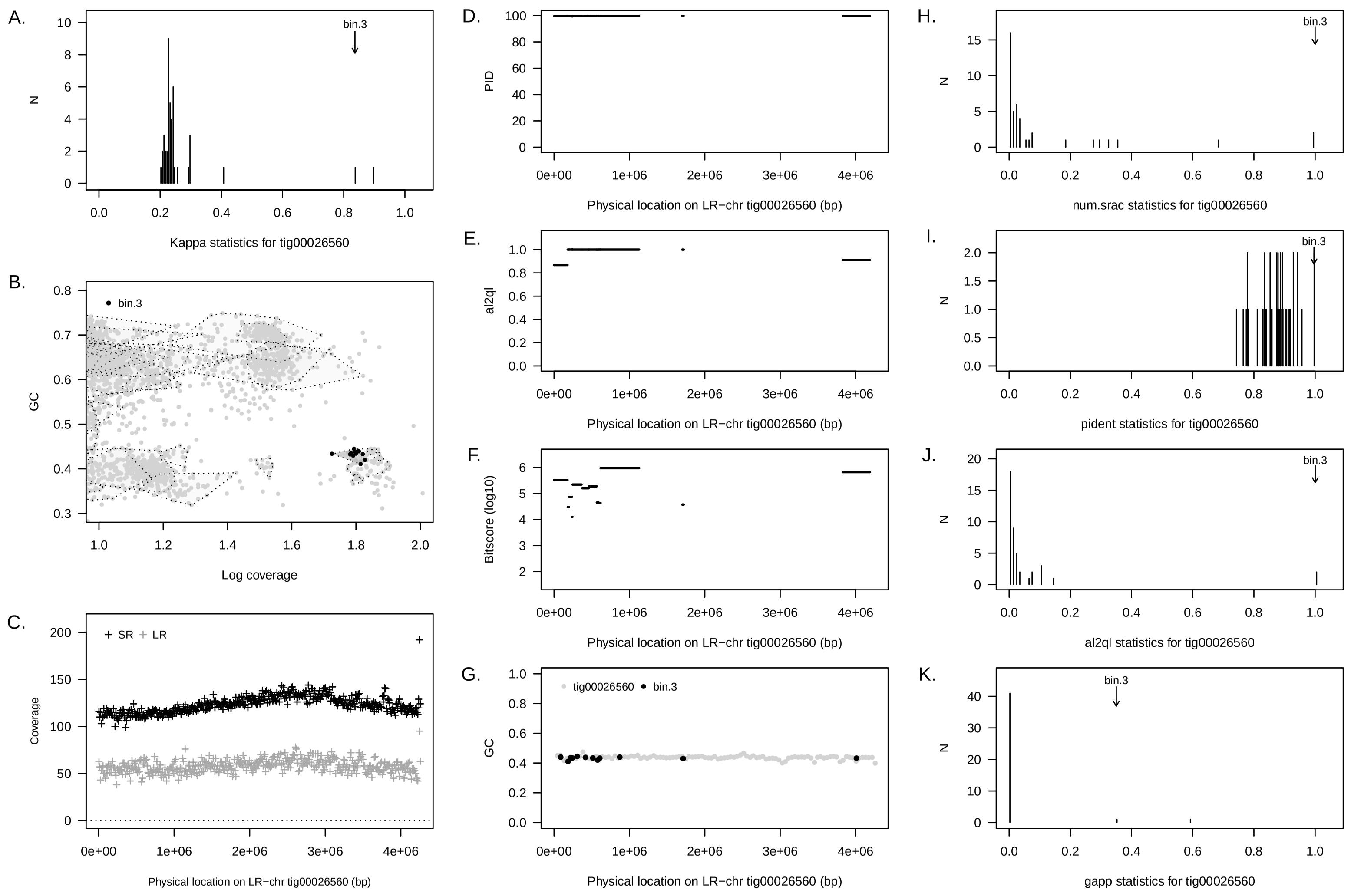

Supplementary Figure 11: Summary of concordance statistic analysis for an LR–chr (`tig00026560`) from the PAO1 reactor community and a short read metagenome assembled genome from the same reactor community (`bin 3`). See Figure 1 for interpretation guide.

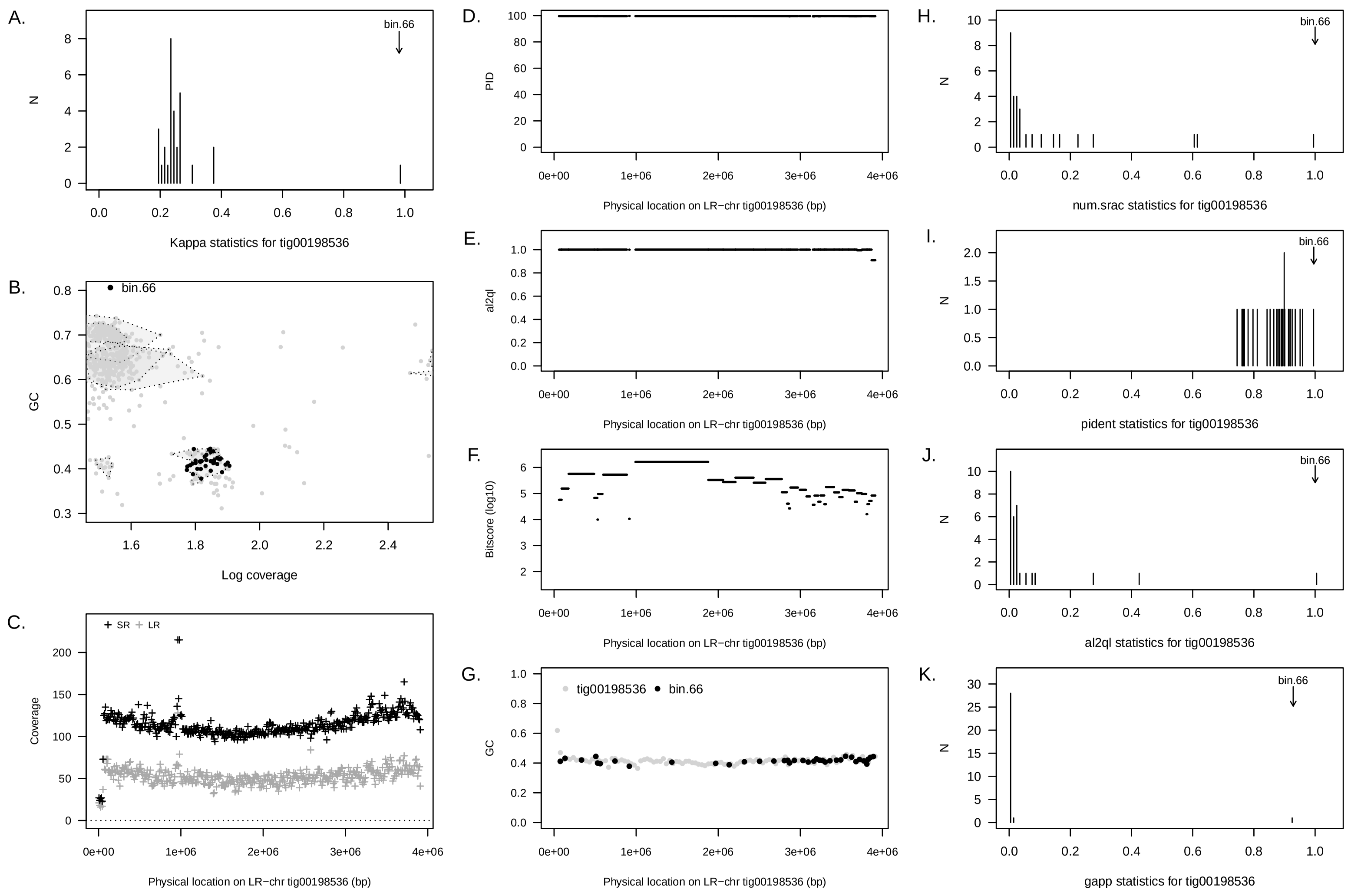

Supplementary Figure 12: Summary of concordance statistic analysis for an LR–chr (`tig00198536`) from the PAO1 reactor community and a short read metagenome assembled genome from the same reactor community (`bin 66`). See Figure 1 for interpretation guide.

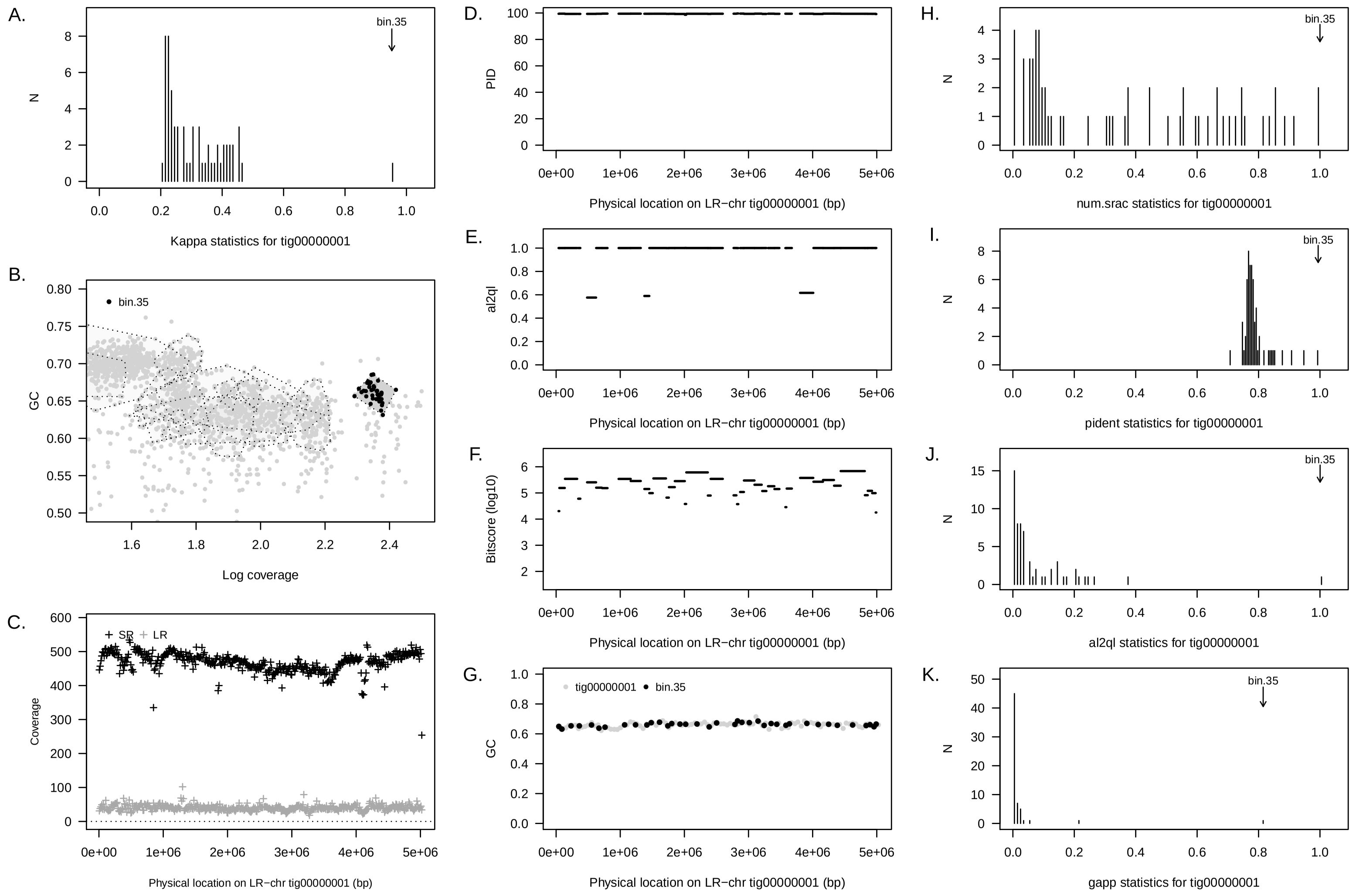

Supplementary Figure 13: Summary of concordance statistic analysis for an LR–chr (`tig00000001`) from the PAO2 reactor community and a short read metagenome assembled genome from the same reactor community (`bin 35`). See Figure 1 for interpretation guide. This genome is annotated to *Candidatus* Accumulibacter and has been subjected to further manual refinement. See main text for details.

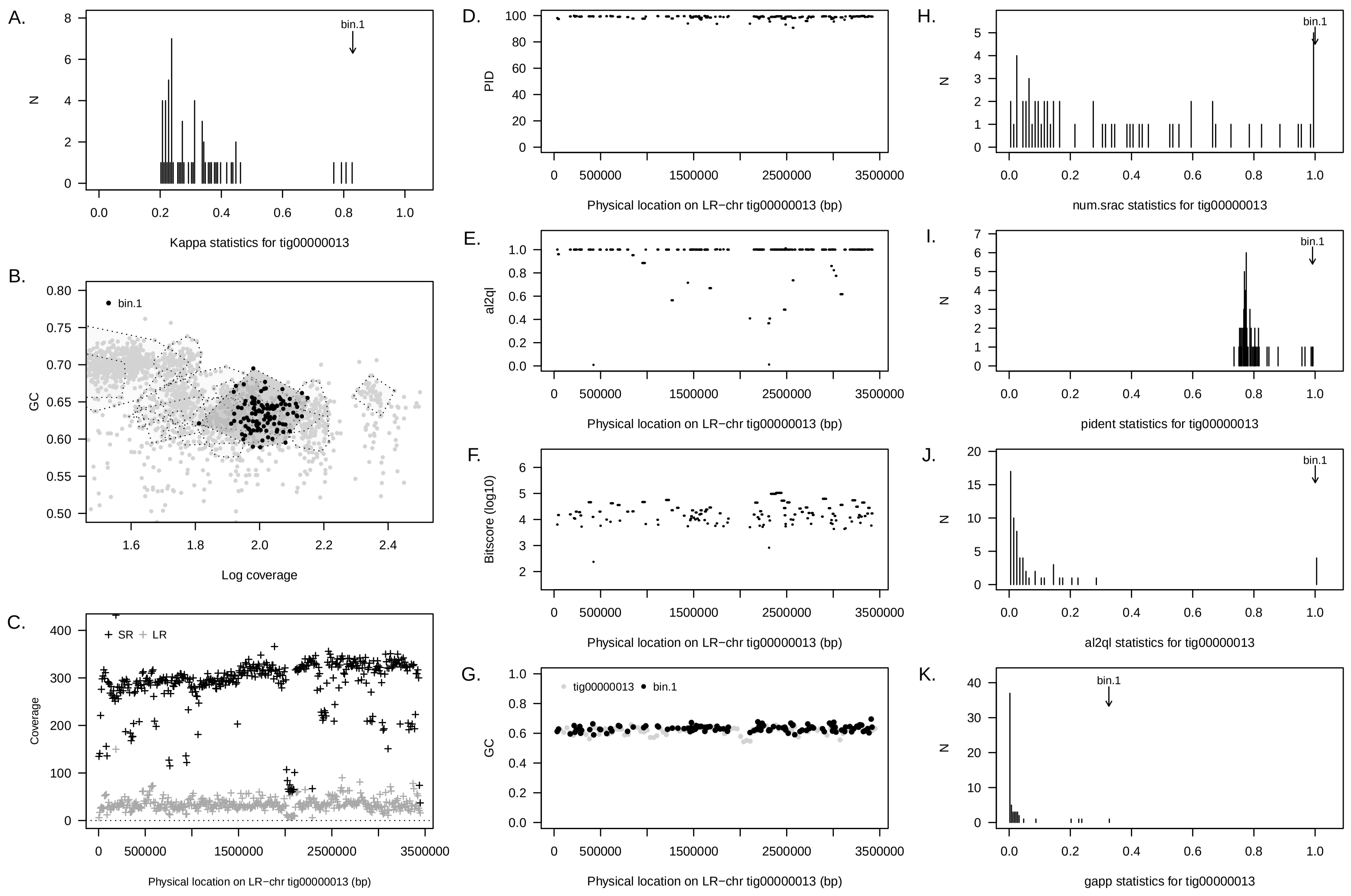

Supplementary Figure 14: Summary of concordance statistic analysis for an LR–chr (`tig00000013`) from the PAO2 reactor community and a short read metagenome assembled genome from the same reactor community (`bin 3`). See Figure 1 for interpretation guide.

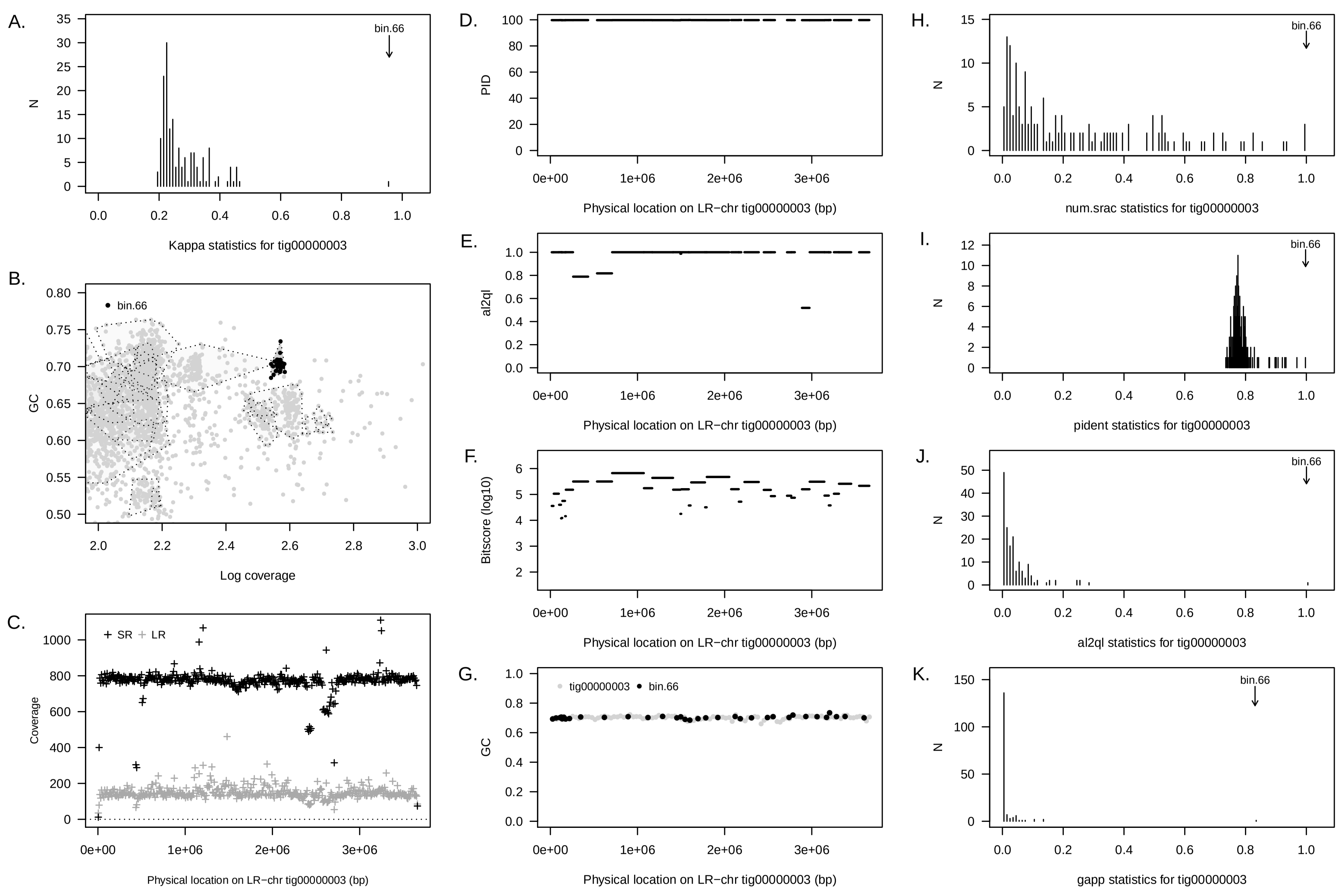

Supplementary Figure 15: Summary of concordance statistic analysis for an LR–chr (`tig00000003`) from the PAO3A reactor community and a short read metagenome assembled genome from the same reactor community (`bin 66`). See Figure 1 for interpretation guide.

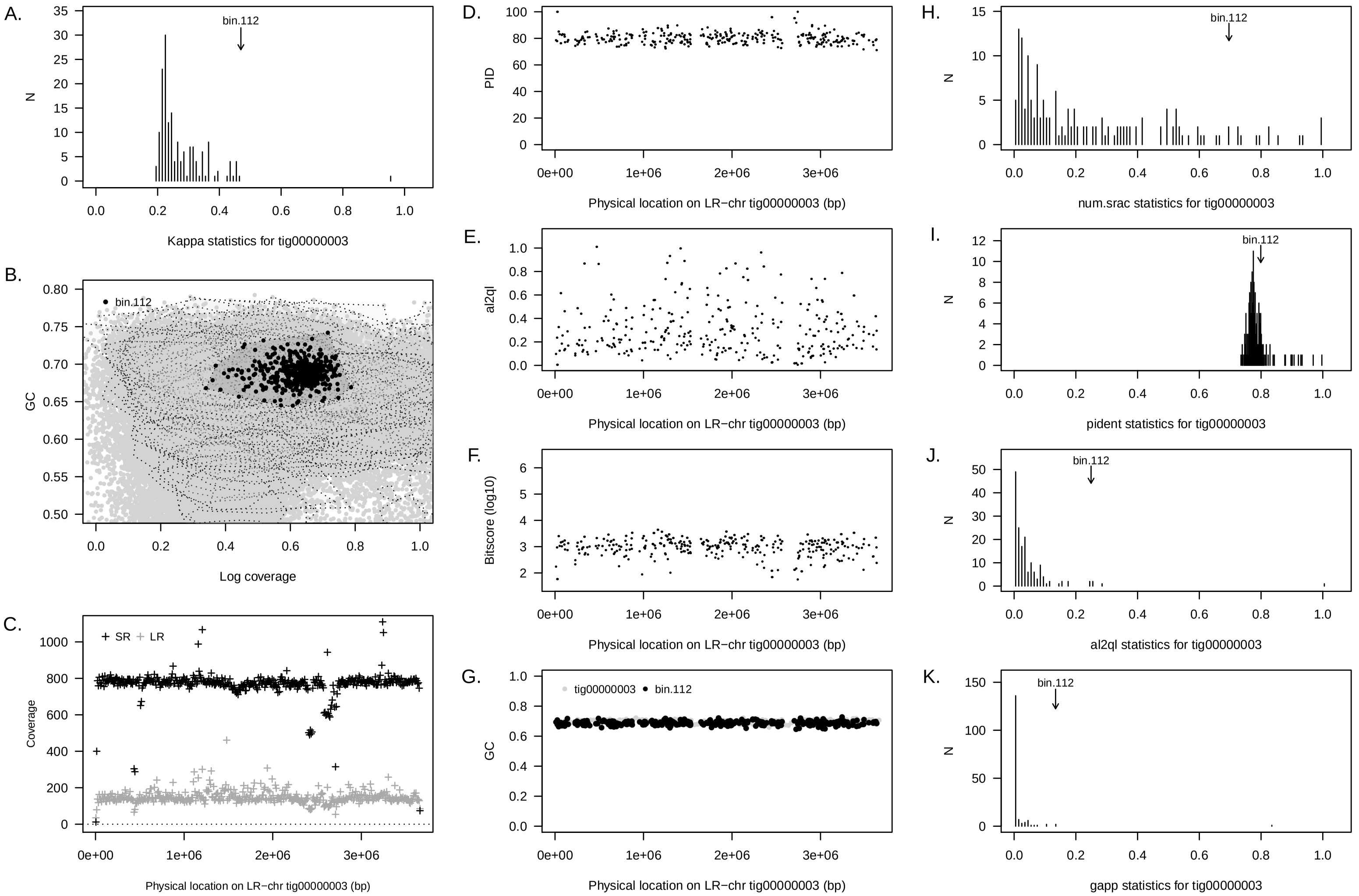

Supplementary Figure 16: Summary of concordance statistic analysis for an LR–chr (`tig00000003`) from the PAO3A reactor community and a short read metagenome assembled genome from the same reactor community (`bin 112`). See Figure 1 for interpretation guide.

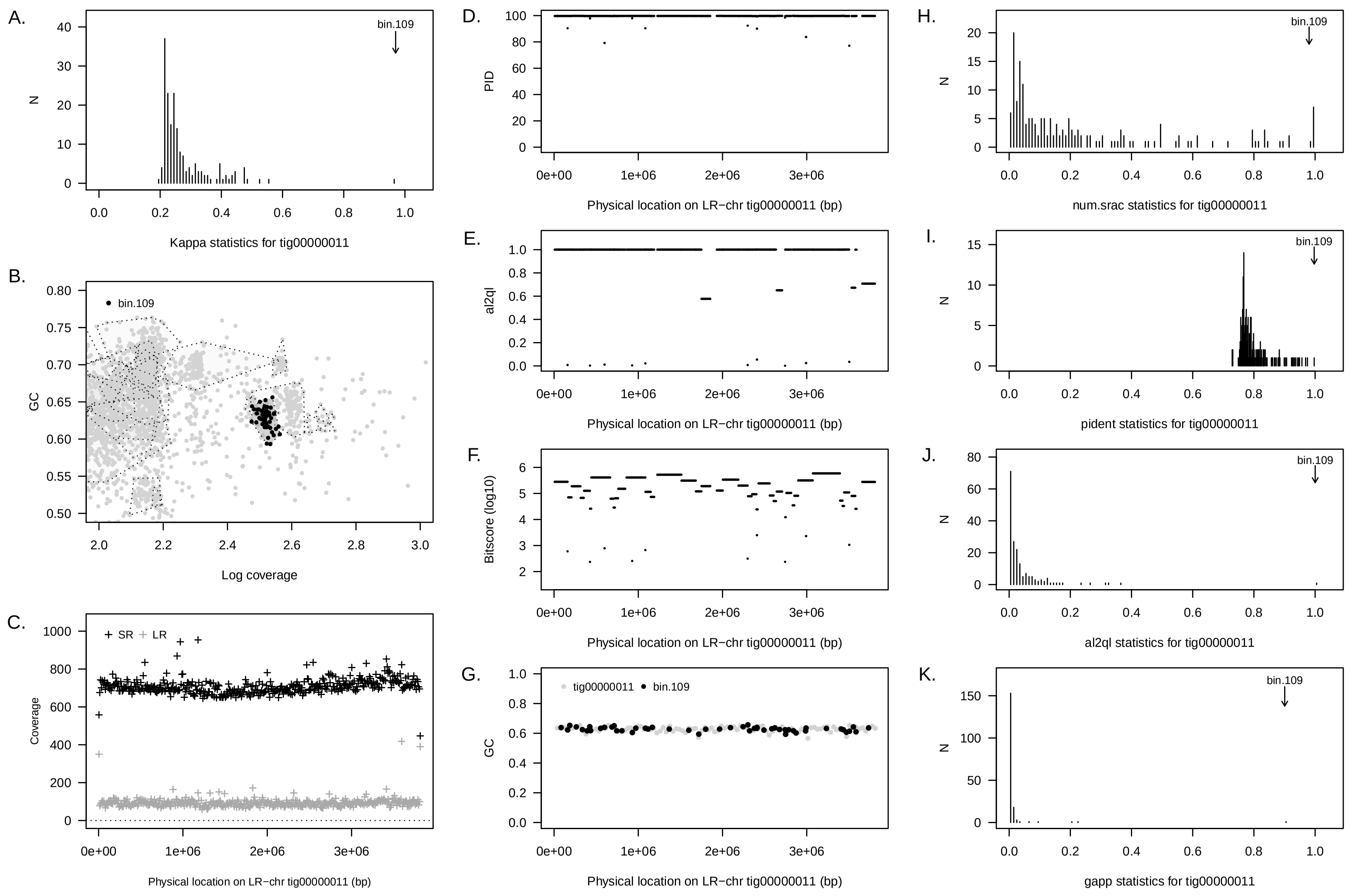

Supplementary Figure 17: Summary of concordance statistic analysis for an LR–chr (`tig00000011`) from the PAO3A reactor community and a short read metagenome assembled genome from the same reactor community (`bin 109`). See Figure 1 for interpretation guide.

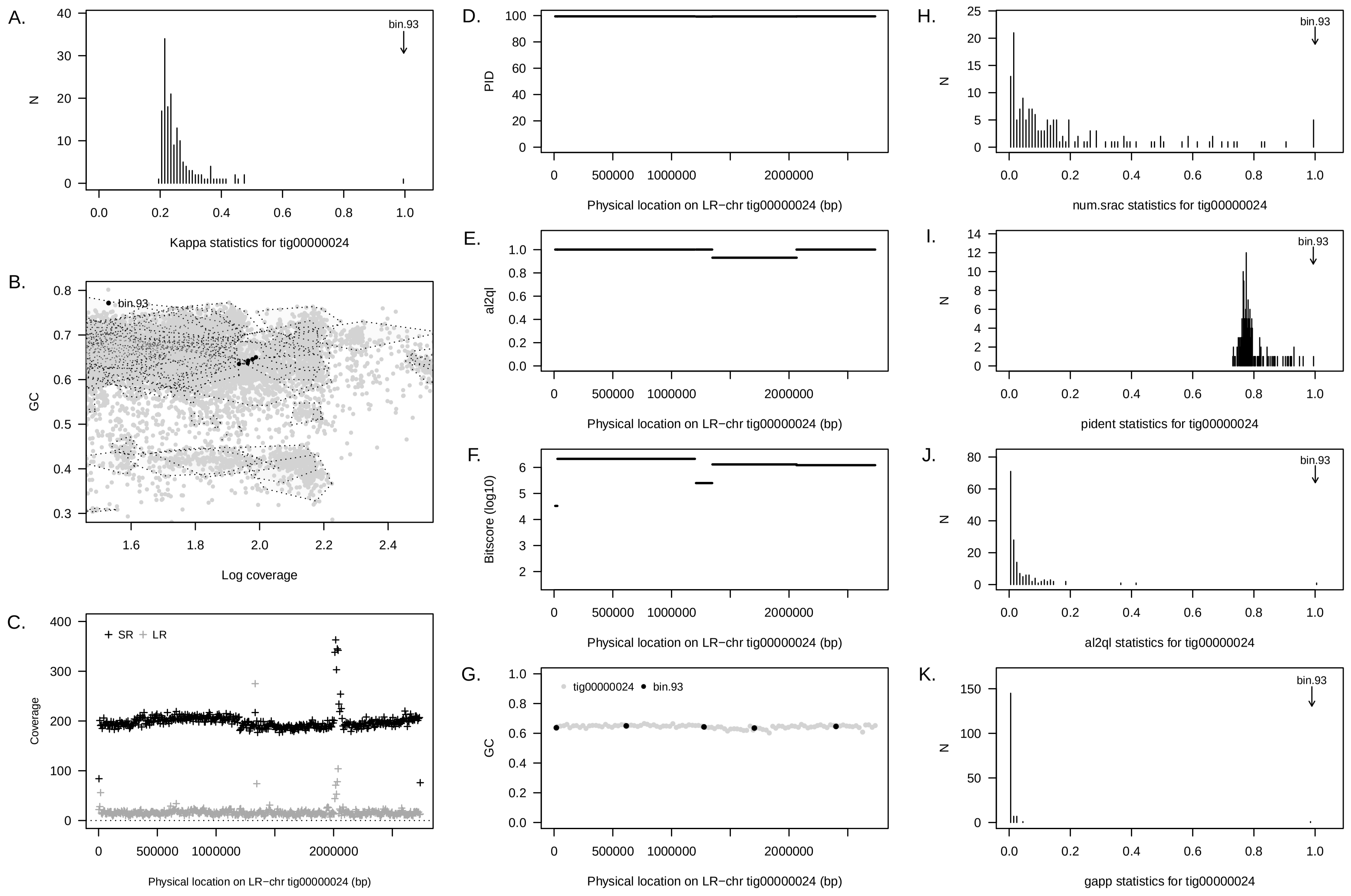

Supplementary Figure 18: Summary of concordance statistic analysis for an LR–chr (`tig00000024`) from the PAO3A reactor community and a short read metagenome assembled genome from the same reactor community (`bin 93`). See Figure 1 for interpretation guide.

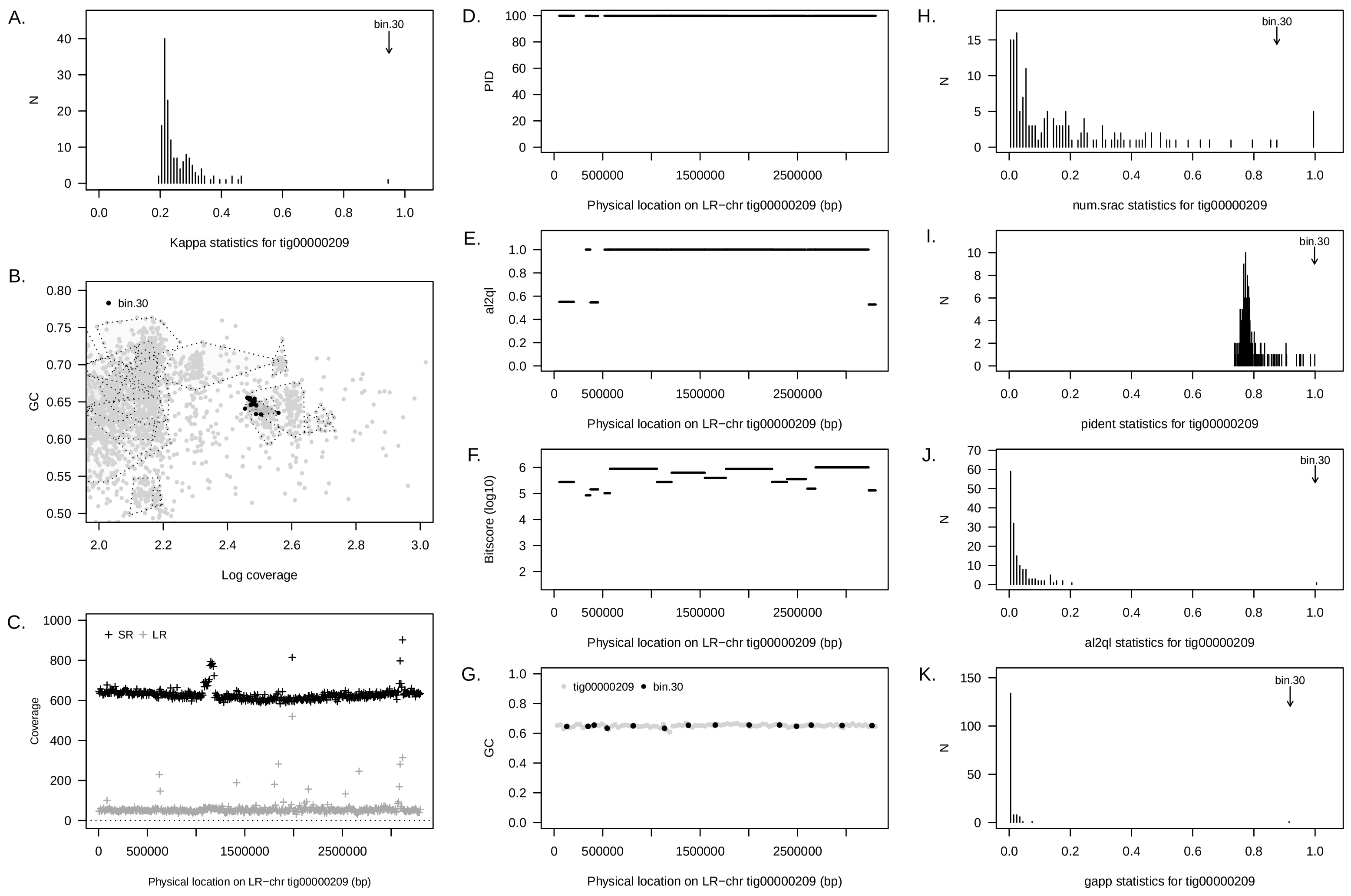

Supplementary Figure 19: Summary of concordance statistic analysis for an LR–chr (`tig00000209`) from the PAO3A reactor community and a short read metagenome assembled genome from the same reactor community (`bin 30`). See Figure 1 for interpretation guide.

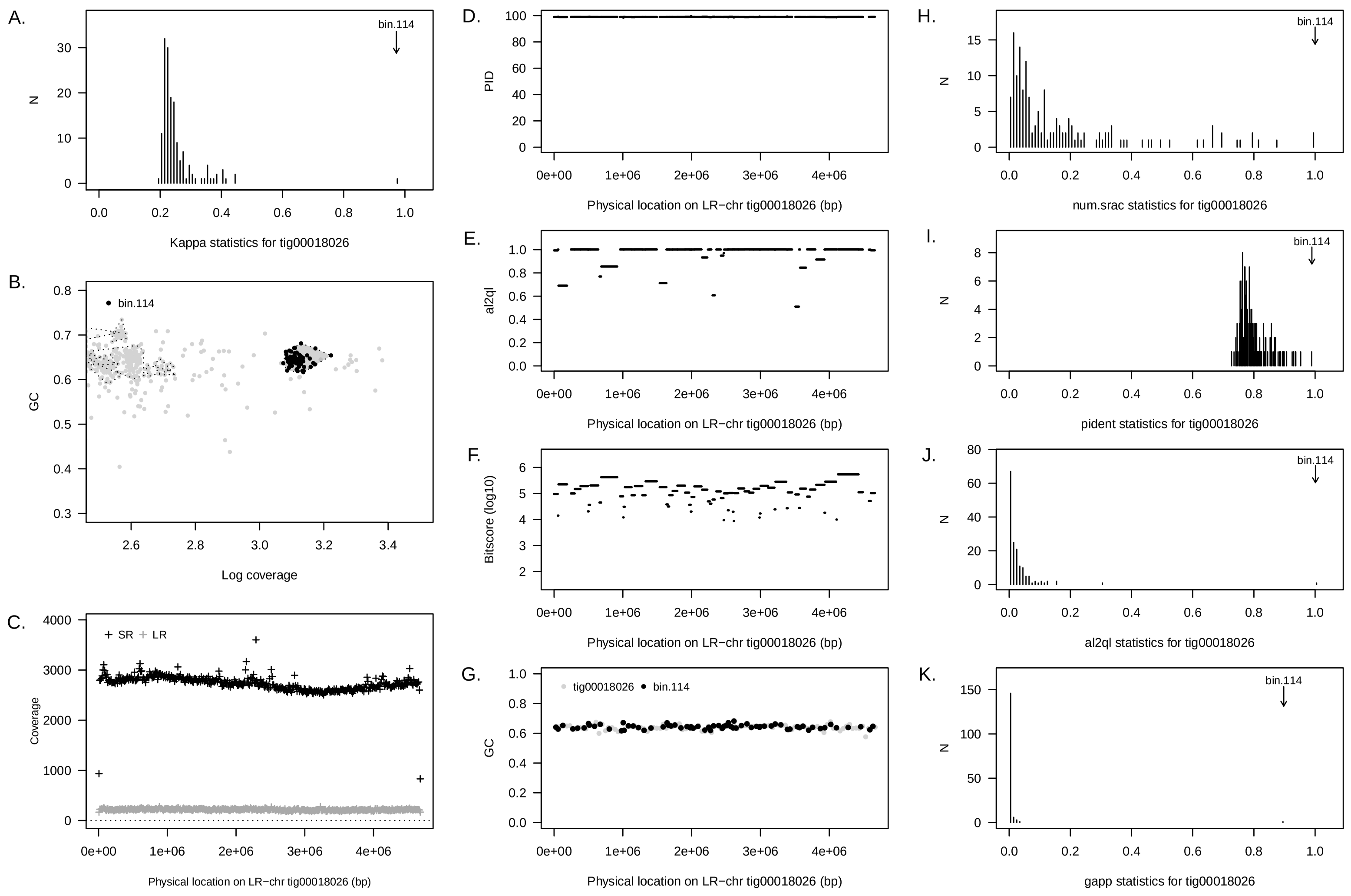

Supplementary Figure 20: Summary of concordance statistic analysis for an LR–chr (`tig00018026`) from the PAO3A reactor community and a short read metagenome assembled genome from the same reactor community (`bin 114`). See Figure 1 for interpretation guide.

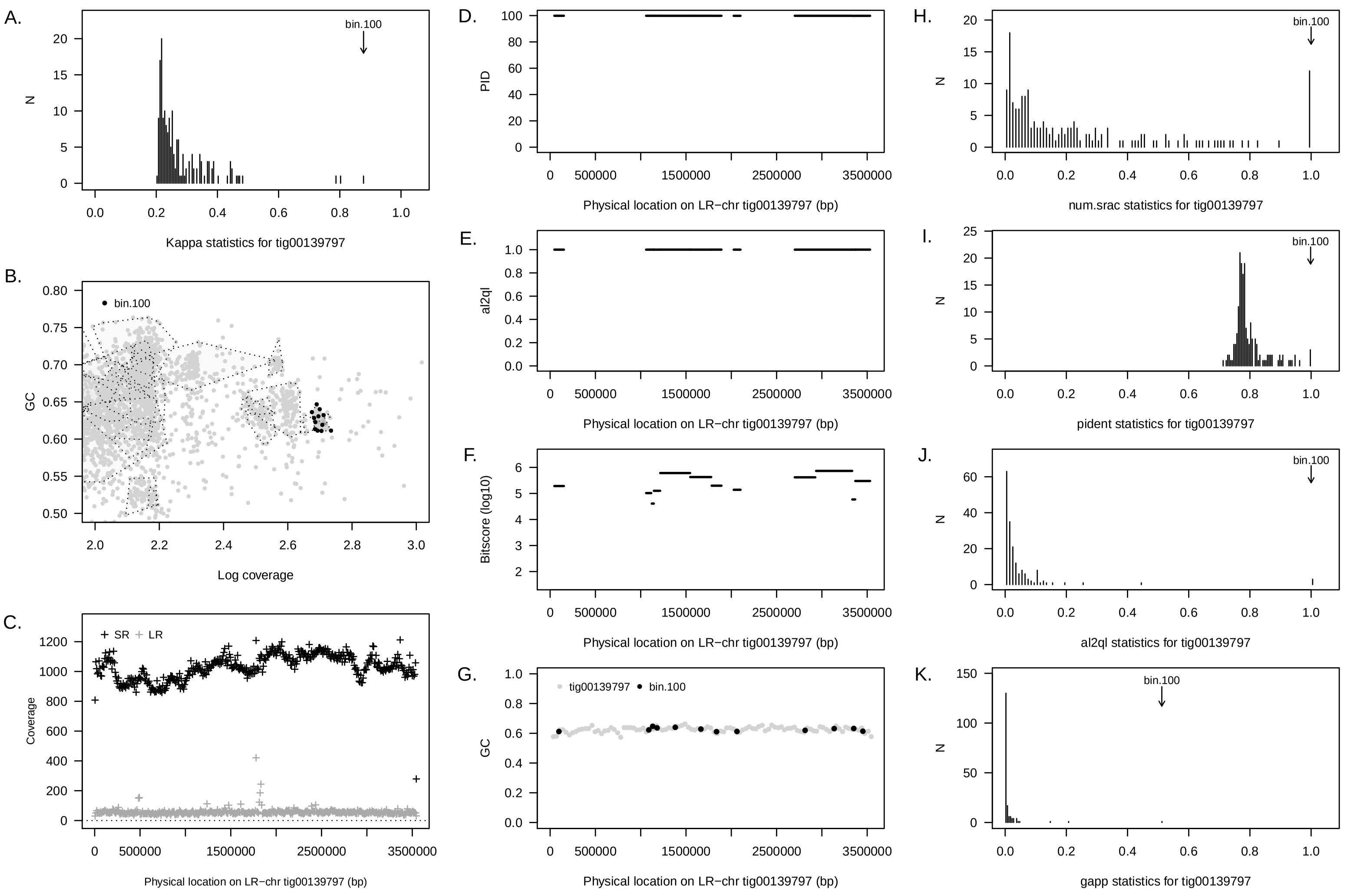

Supplementary Figure 21: Summary of concordance statistic analysis for an LR–chr (`tig00139797`) from the PAO3A reactor community and a short read metagenome assembled genome from the same reactor community (`bin 100`). See Figure 1 for interpretation guide.

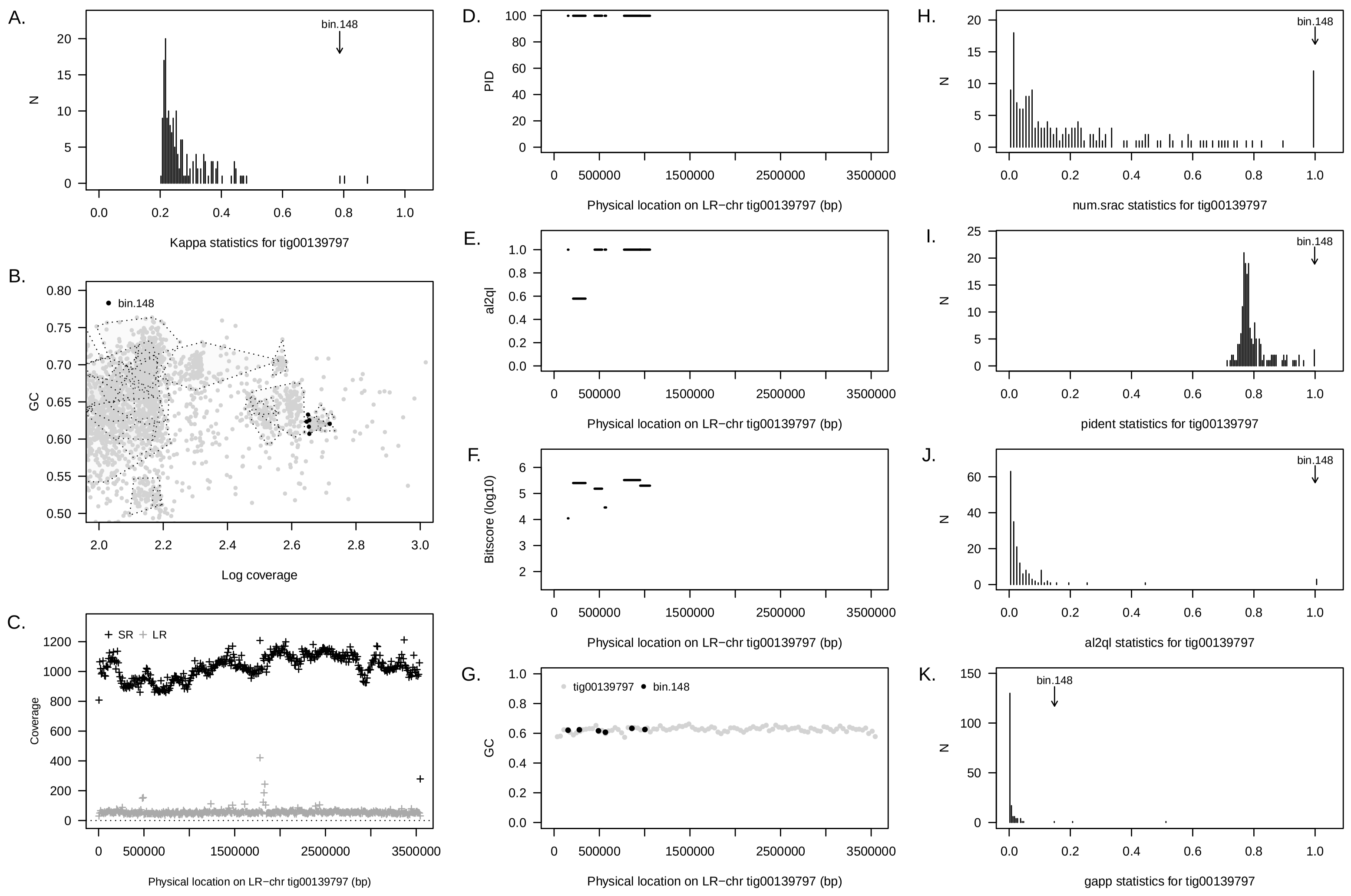

Supplementary Figure 22: Summary of concordance statistic analysis for an LR–chr (`tig00139797`) from the PAO3A reactor community and a short read metagenome assembled genome from the same reactor community (`bin 148`). See Figure 1 for interpretation guide.

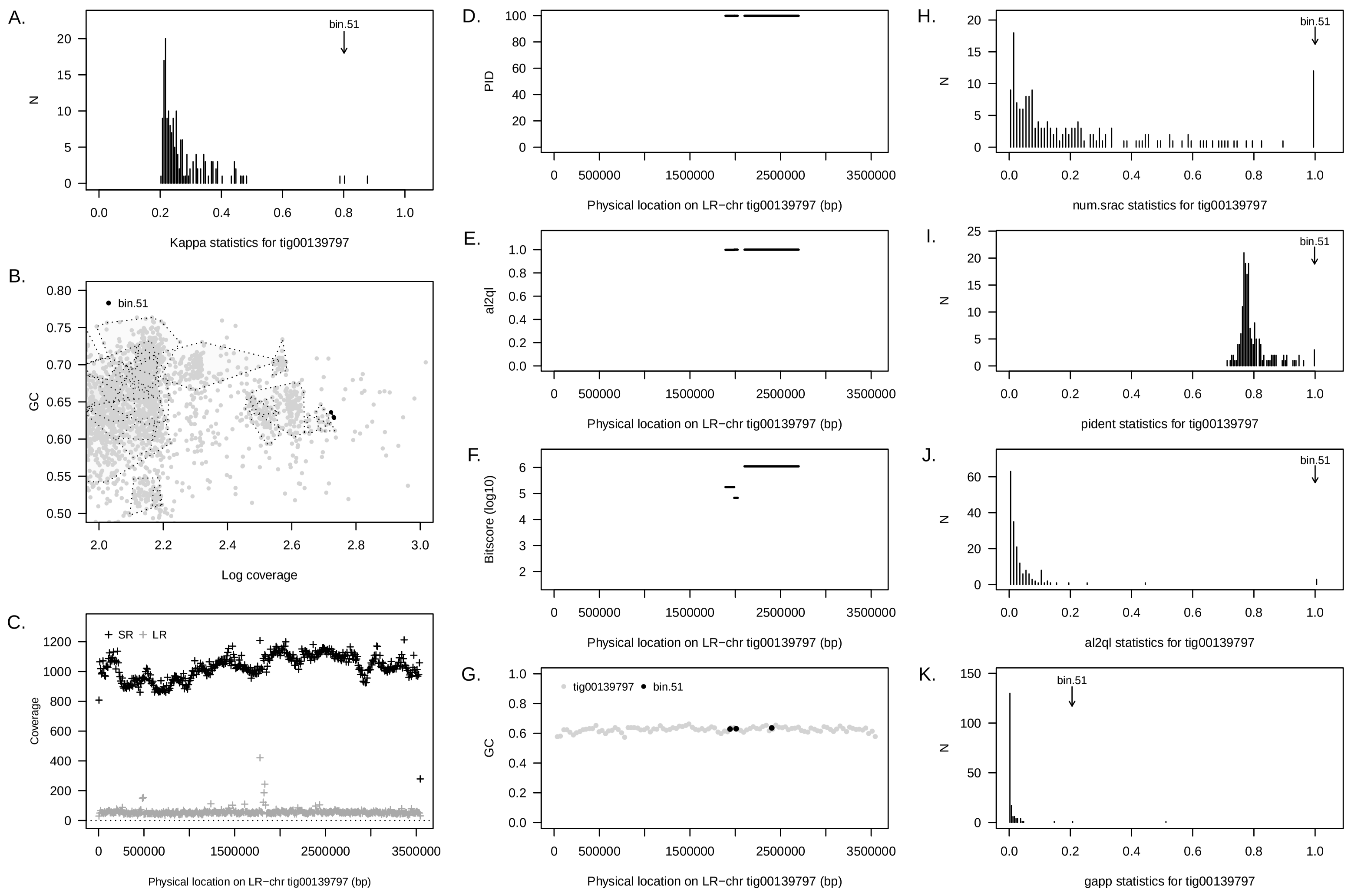

Supplementary Figure 23: Summary of concordance statistic analysis for an LR–chr (`tig00139797`) from the PAO3A reactor community and a short read metagenome assembled genome from the same reactor community (`bin 51`). See Figure 1 for interpretation guide.

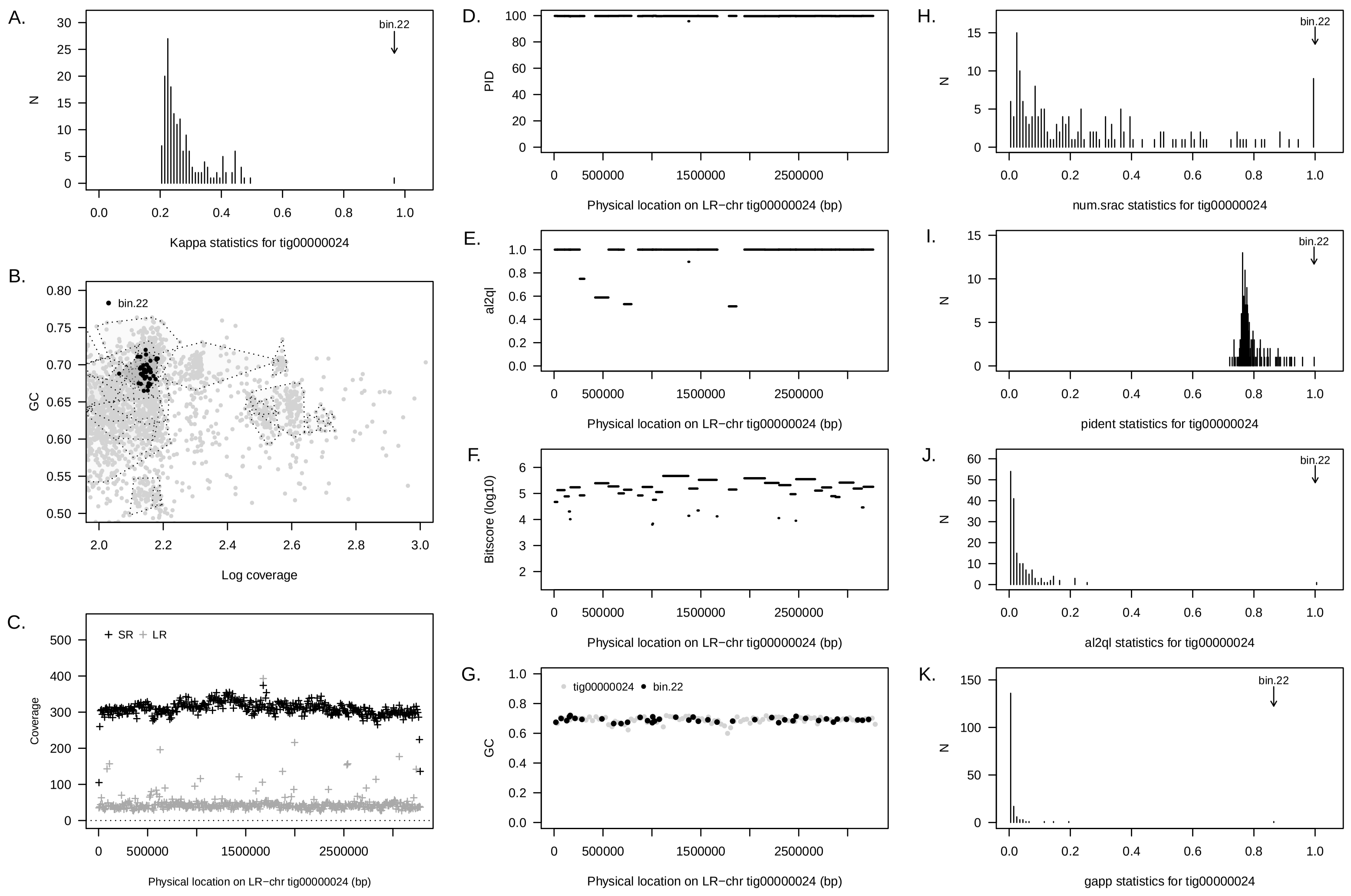

Supplementary Figure 24: Summary of concordance statistic analysis for an LR–chr (`tig00000024`) from the PAO3B reactor community and a short read metagenome assembled genome from the same reactor community (`bin 22`). See Figure 1 for interpretation guide.

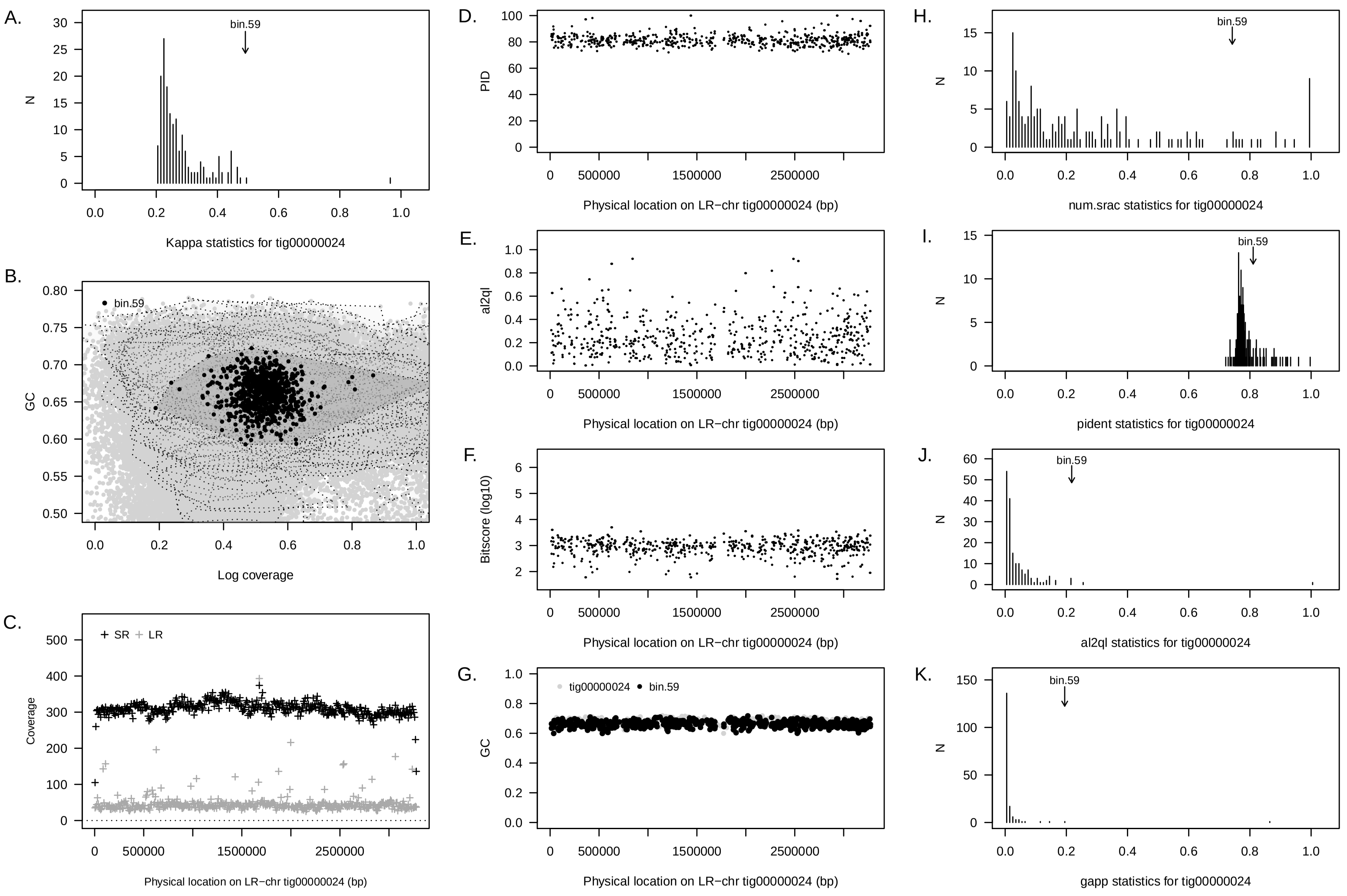

Supplementary Figure 25: Summary of concordance statistic analysis for an LR–chr (`tig00000024`) from the PAO3B reactor community and a short read metagenome assembled genome from the same reactor community (`bin 59`). See Figure 1 for interpretation guide.

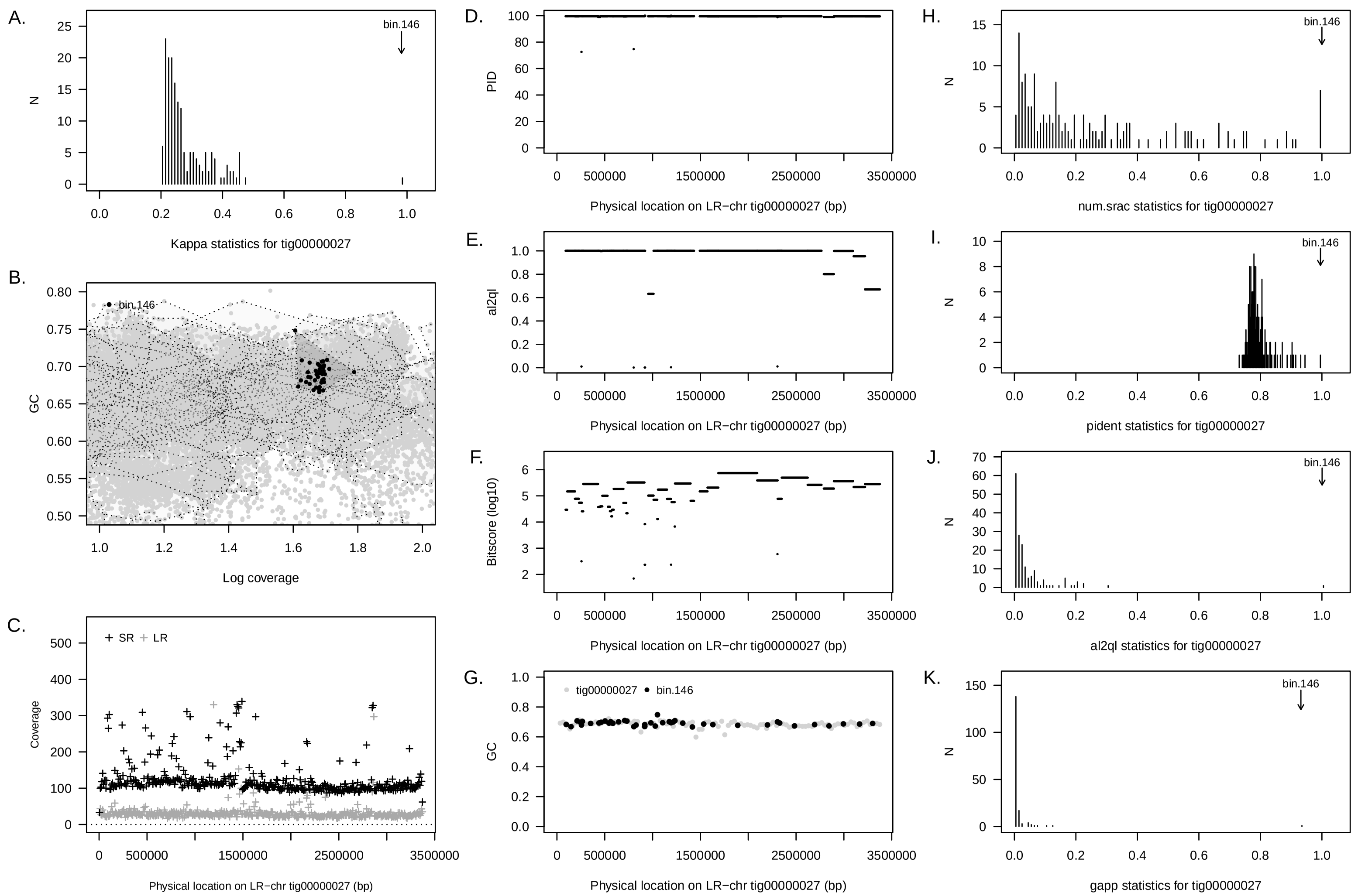

Supplementary Figure 26: Summary of concordance statistic analysis for an LR–chr (`tig00000027`) from the PAO3B reactor community and a short read metagenome assembled genome from the same reactor community (`bin 146`). See Figure 1 for interpretation guide.

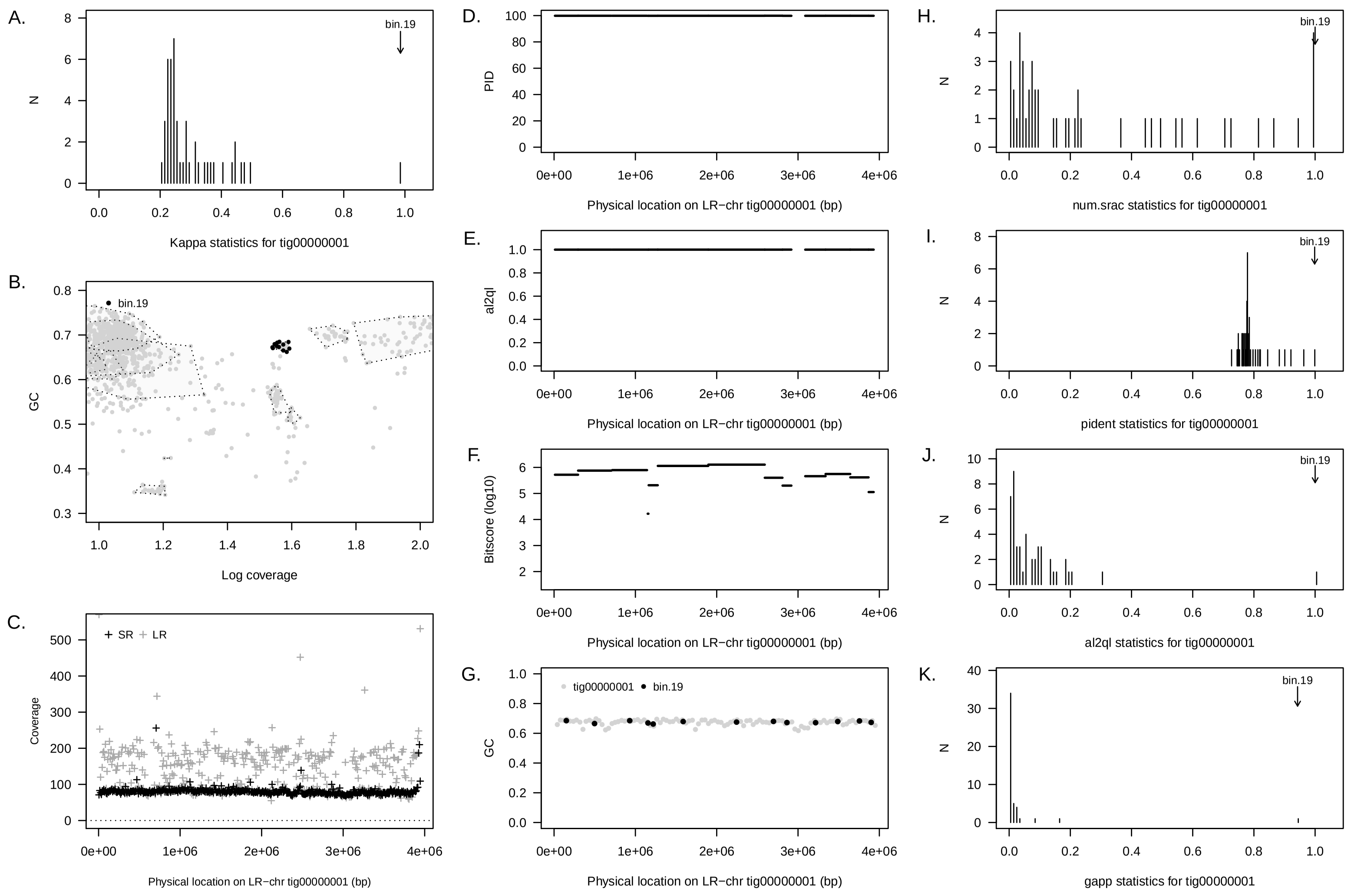

Supplementary Figure 27: Summary of concordance statistic analysis for an LR–chr (`tig00000001`) from the PAO4 reactor community and a short read metagenome assembled genome from the same reactor community (`bin 19`). See Figure 1 for interpretation guide.

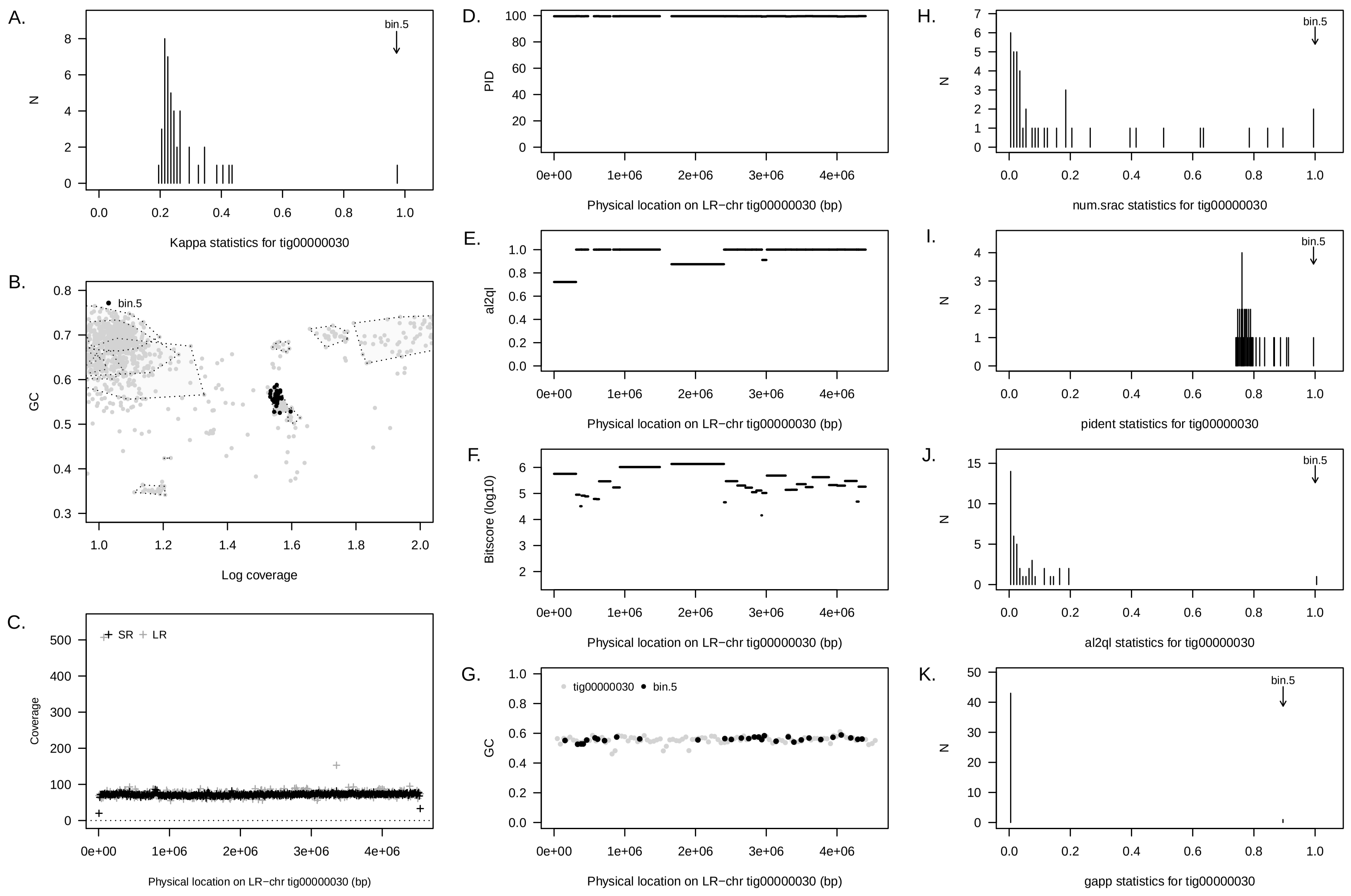

Supplementary Figure 28: Summary of concordance statistic analysis for an LR–chr (`tig00000030`) from the PAO4 reactor community and a short read metagenome assembled genome from the same reactor community (`bin 5`). See Figure 1 for interpretation guide.

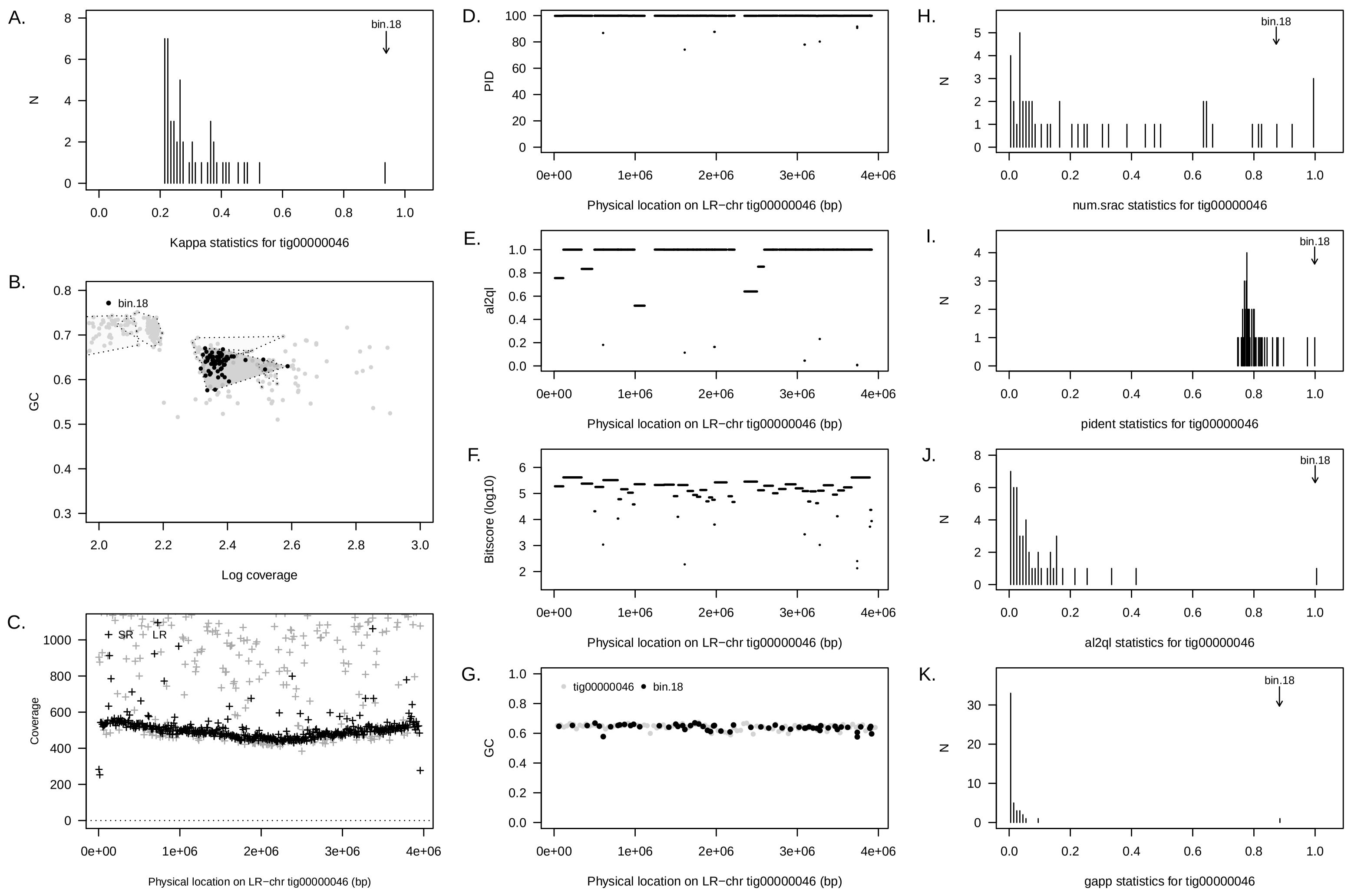

Supplementary Figure 29: Summary of concordance statistic analysis for an LR–chr (`tig00000046`) from the PAO4 reactor community and a short read metagenome assembled genome from the same reactor community (`bin 18`). See Figure 1 for interpretation guide.

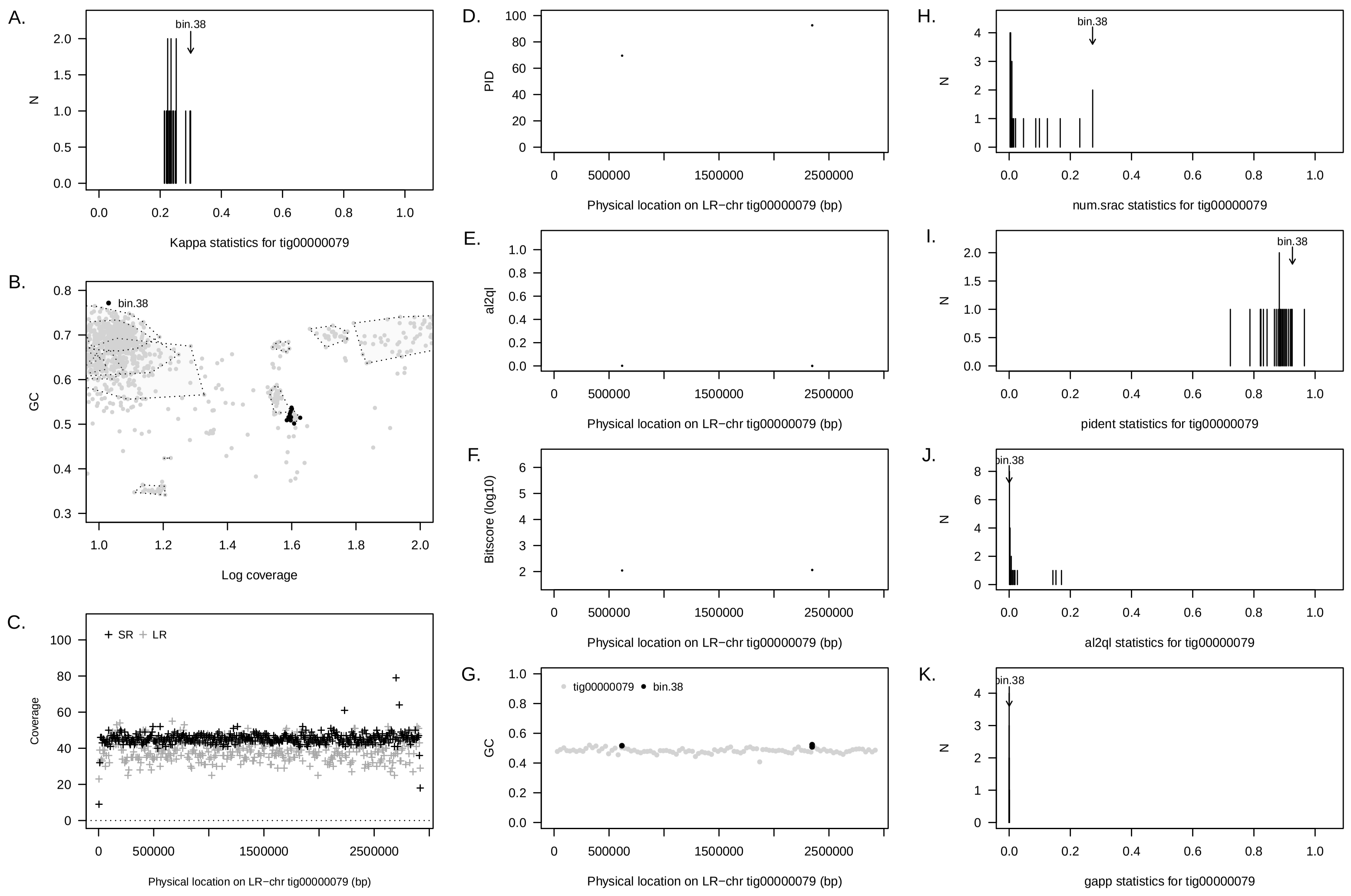

Supplementary Figure 30: Summary of concordance statistic analysis for an LR–chr (`tig00000079`) from the PAO4 reactor community and a short read metagenome assembled genome from the same reactor community (`bin 38`). See Figure 1 for interpretation guide.

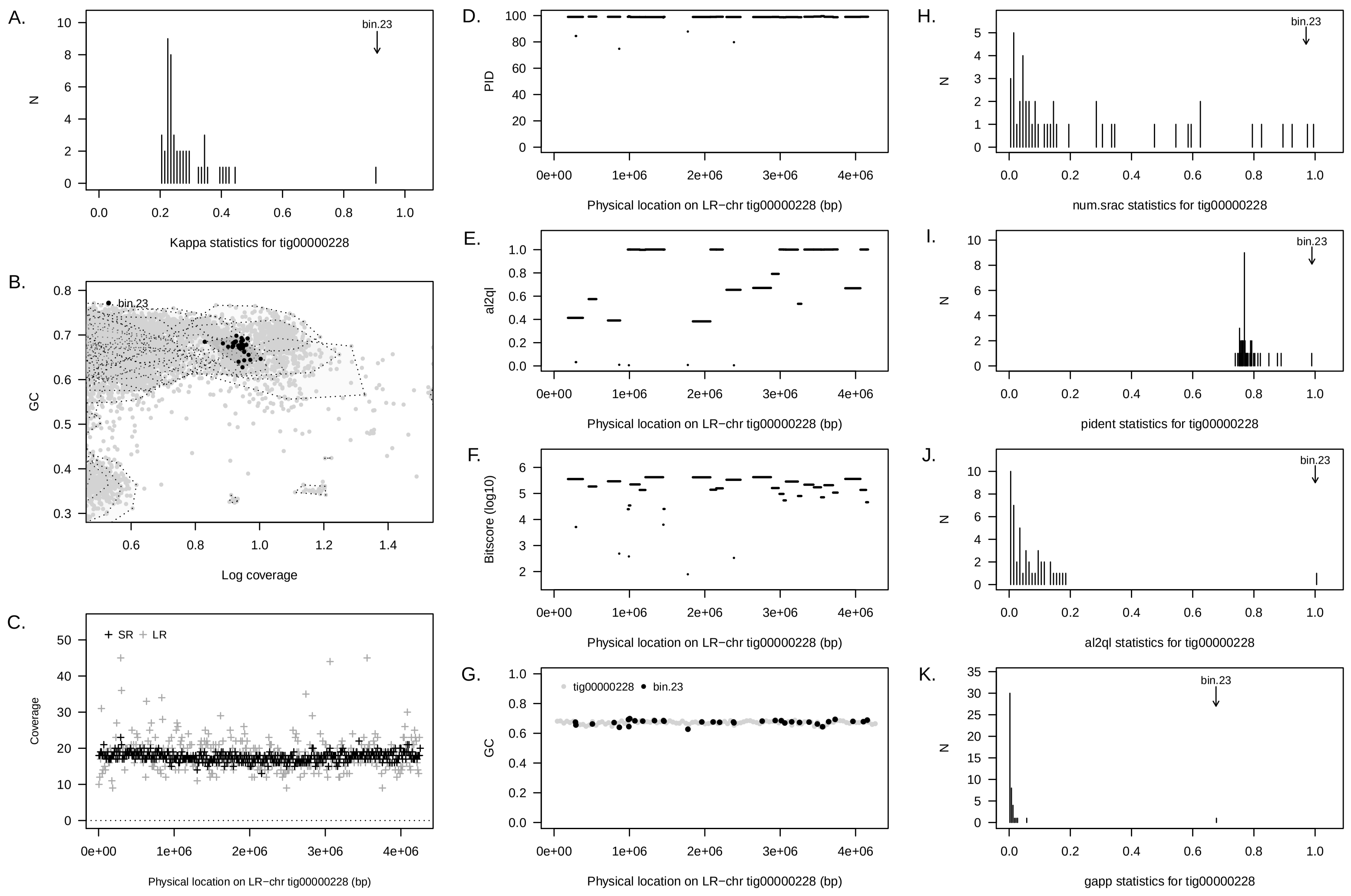

Supplementary Figure 31: Summary of concordance statistic analysis for an LR–chr (`tig000000228`) from the PAO4 reactor community and a short read metagenome assembled genome from the same reactor community (`bin 23`). See Figure 1 for interpretation guide.

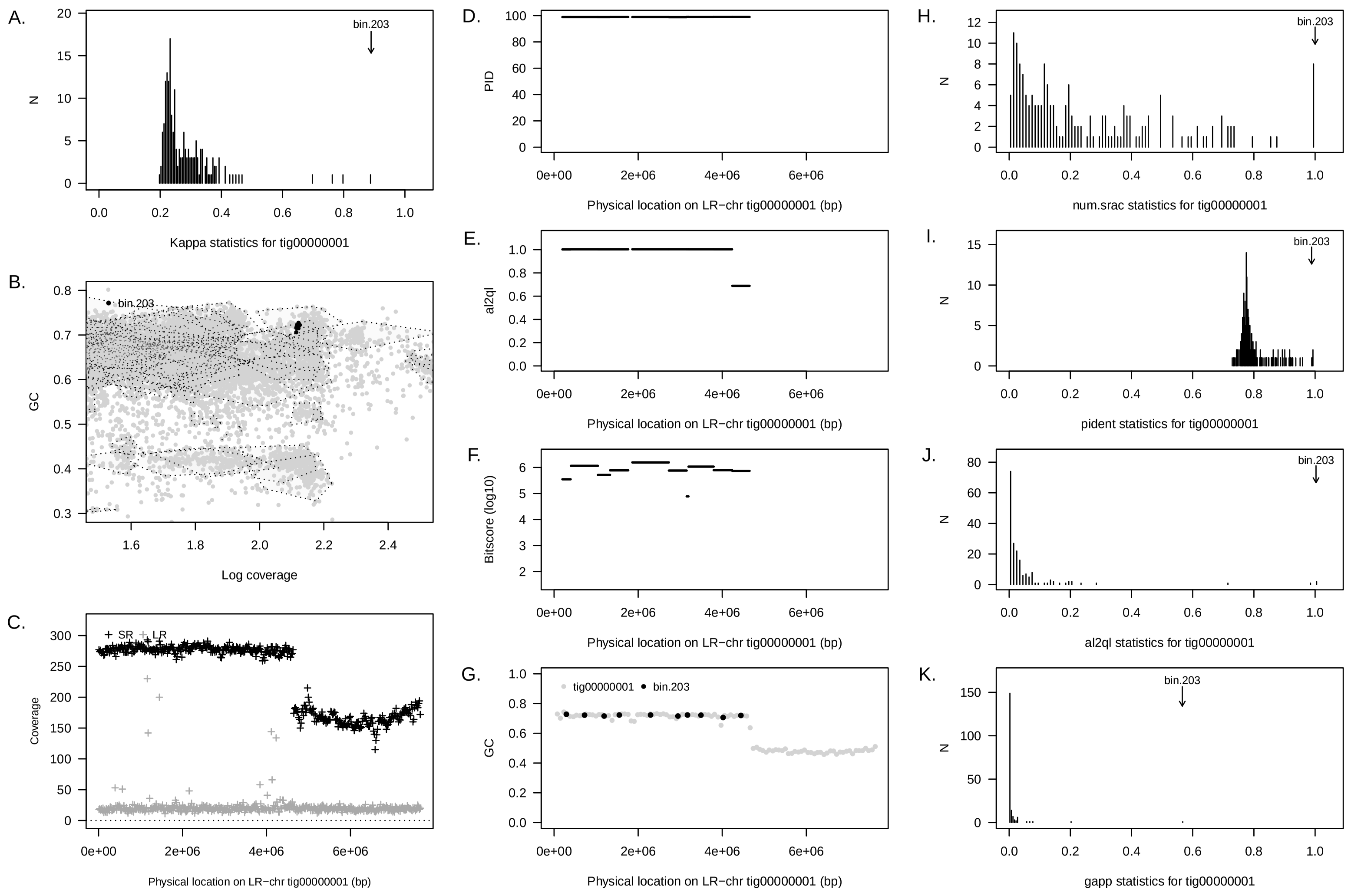

Supplementary Figure 32: Summary of concordance statistic analysis for an artefactual LR–chr (`tig000000001`) from the PAO3A reactor community and a short read metagenome assembled genome from the same reactor community (`bin 203`). See Figure 1 for interpretation guide.

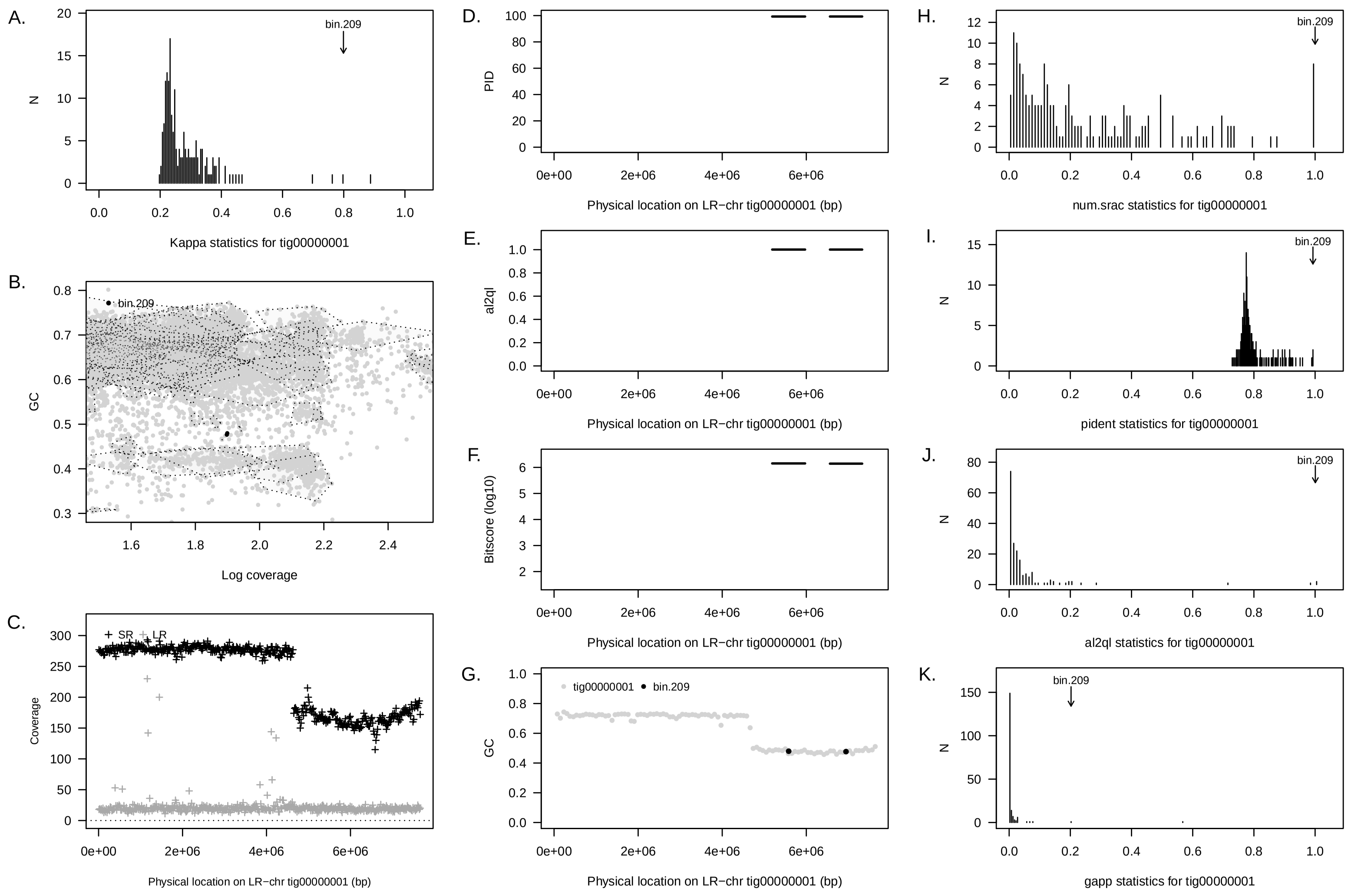

Supplementary Figure 33: Summary of concordance statistic analysis for an artefactual LR–chr (`tig000000001`) from the PAO3A reactor community and a short read metagenome assembled genome from the same reactor community (`bin 209`). See Figure 1 for interpretation guide.

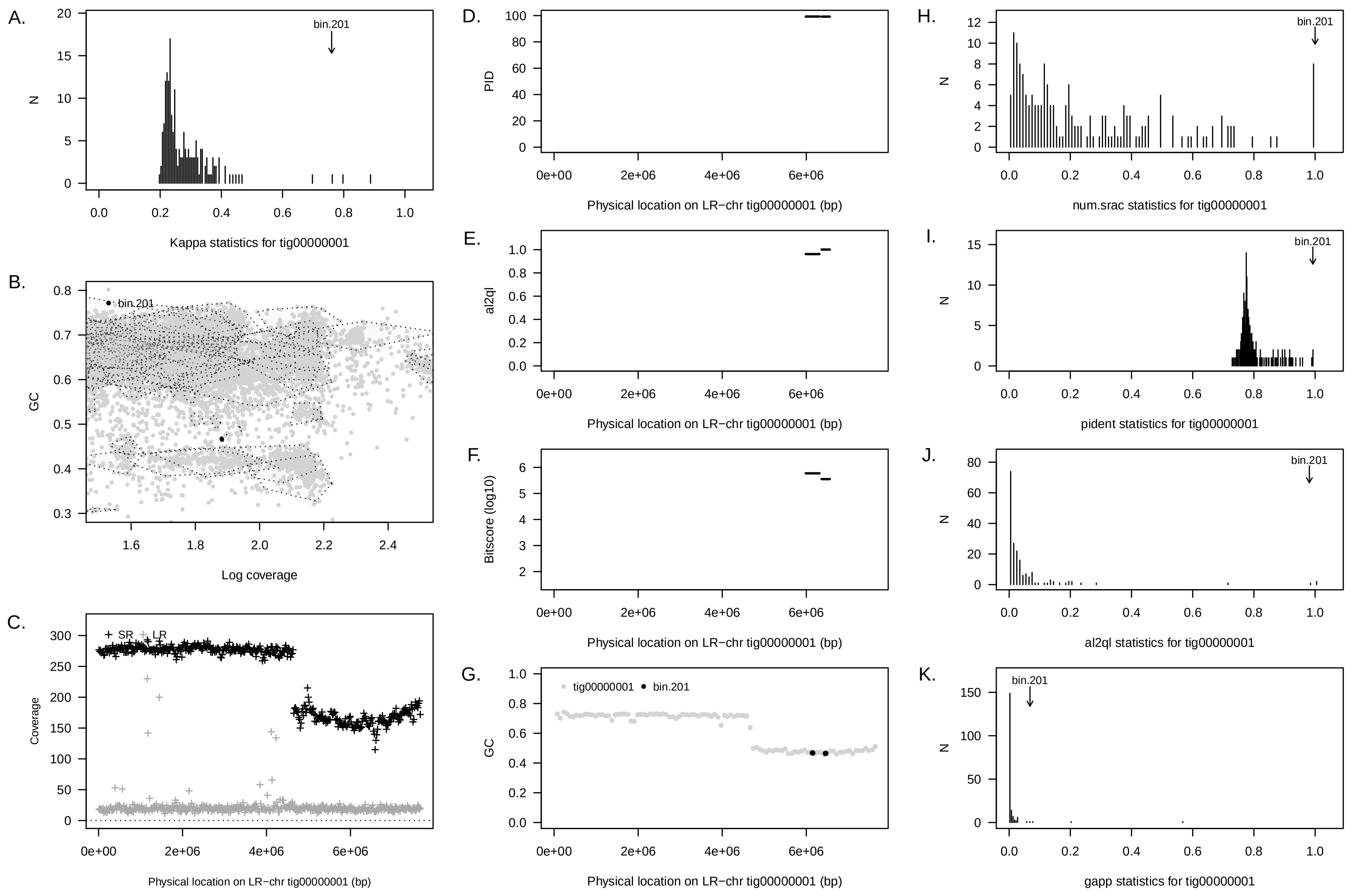

Supplementary Figure 34: Summary of concordance statistic analysis for an artefactual LR–chr (`tig000000001`) from the PAO3A reactor community and a short read metagenome assembled genome from the same reactor community (`bin 201`). See Figure 1 for interpretation guide.

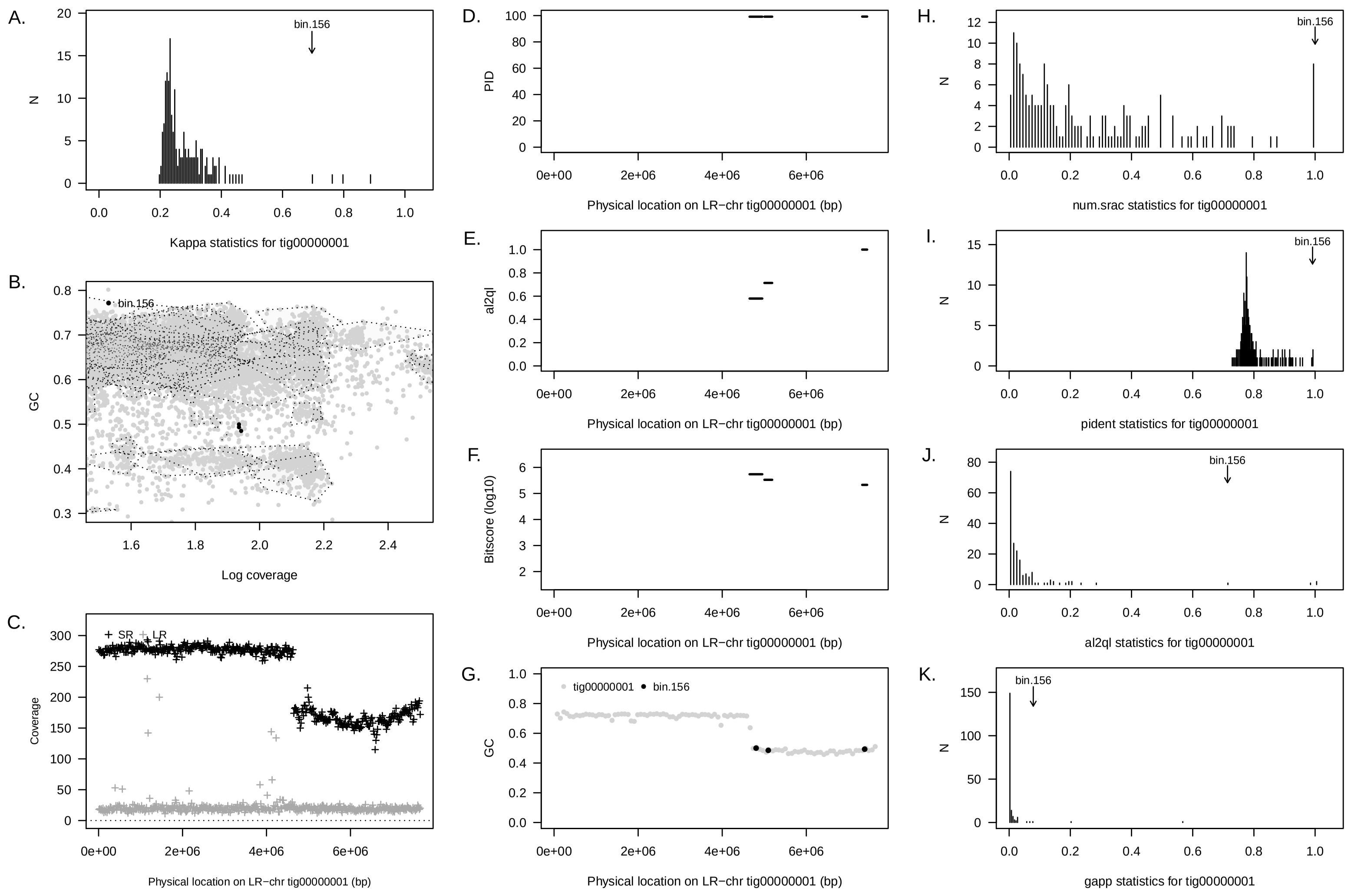

Supplementary Figure 35: Summary of concordance statistic analysis for an artefactual LR–chr (`tig000000001`) from the PAO3A reactor community and a short read metagenome assembled genome from the same reactor community (`bin 156`). See Figure 1 for interpretation guide.

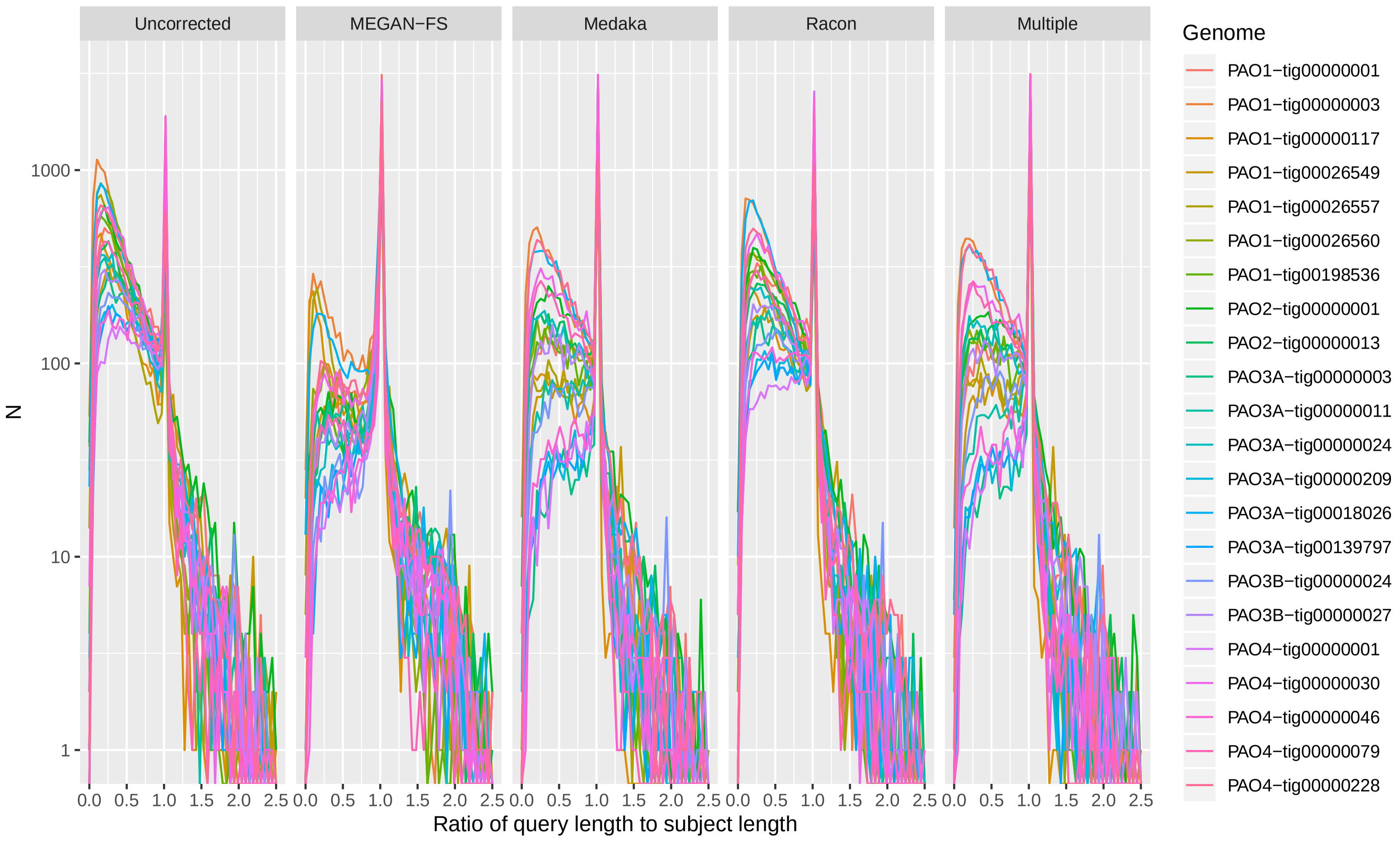

Supplementary Figure 36: Modified version of **Figure 2** using a log-scale on y–axis

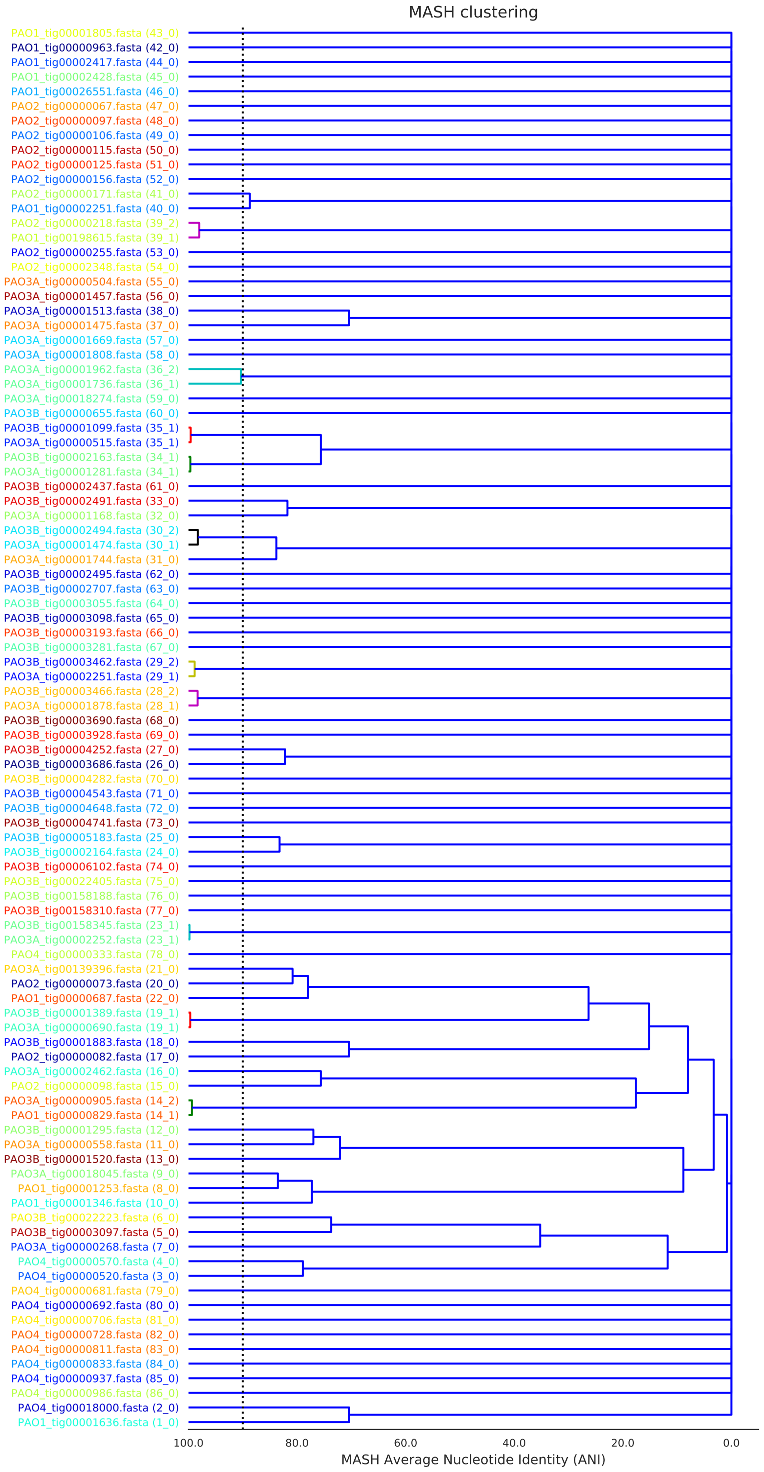

Supplementary Figure 37: Dendrogram generated from MASH analysis (`dRep`) of circular LRAC sequences less than 1Mbp in length.

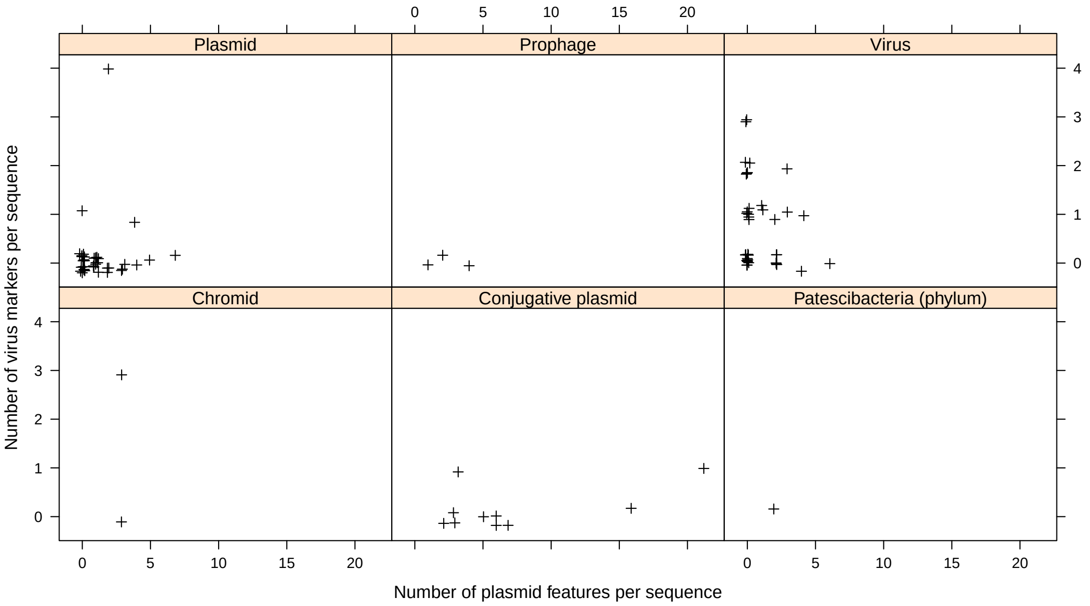

Supplementary Figure 38: Relationship between number of virus–associated genetic features and plasmid–associated genetic features for circular LRAC sequences less than 1Mbp in length, categorised by sequence type (four short sequences likely to be artefacts are not included).

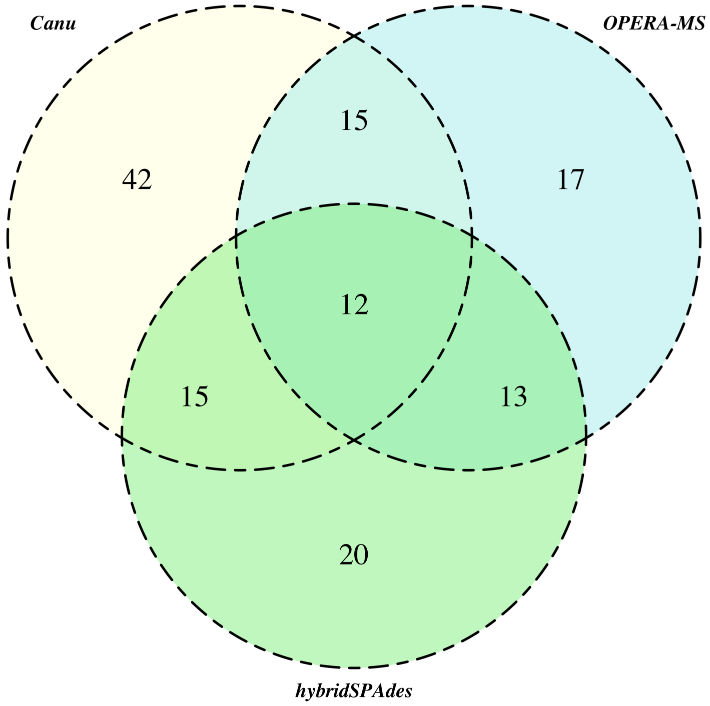

Supplementary Figure 39: Number of secondary clusters (dRep) formed by 240 LR–chr and HY-chr sequences categorised by contributing workflow.

Captions for Supplementary Data Sets

Supplementary Data 1
Summary of concordance statistic analysis for recovered genomes.

Supplementary Data 2
Taxonomic annotations of recovered genomes using GTDB–Tk.

Supplementary Data 3
Taxonomic annotations for 16S-SSU rRNA genes identified in recovered genomes.

Supplementary Data 4
Comparison of whole–genome taxonomic annotation from GTDB with 16S–SSU rRNA gene annotation for recovered genomes.

Supplementary Data 5
Annotation results for 96 short circular LRAC sequences <1Mbp in length (columns E-F from Prokka; G-P from KEGG annotations; Q-AB from CheckV.

Supplementary Data 6
BlastN results of circular sequences <1Mbp mapped to RefSeq plasmid database.

Supplementary Data 7
BlastN results of circular sequences <1Mbp mapped to RefSeq viral database (Worksheet 1) and IMG/VR database (Worksheet 2).

Supplementary Data 8
Genome quality statistics and taxonomic annotation of HY–chr sequences obtained from hybrid workflows, and inter–relationships with LR–chr sequence from Canu.

Supplementary Data 9
Number of HY– and/or LR–chr sequences from dRep secondary cluster analysis.

Supplementary Data 10
Taxonomic annotations of short read bins using GTDB–Tk (results from each datasets are provided in separate worksheets)

GenBank assembly accession identifiers used in Figure 3

SCELSE1 (GCA_005524045.1)
BA94 (GCA_000585095.1)
UBA2327 (GCA_002345025.1)
SK11 (GCA_000584995.1)
UBA8770 (GCA_003487685.1)
UBA11070 (GCA_003535635.1)
UBA9001 (GCA_003542235.1)
UBA2315 (GCA_002345285.1)
SK12 (GCA_000585015.1)
UBA6585 (GCA_003535635.1)
Banfield (GCA_001897745.1)
UBA11064 (GCA_003538495.1)
BA91 (GCA_000585035.2)
Mardanov (GCA_005889575.1)
SK02 (GCA_000584975.2)
HKU2 (GCA_000987395.1)
UBA5574 (GCA_002425405.1)
SK01 (GCA_000584955.2)
UW1 (GCA_000024165.1)
UBA2783 (GCA_002352265.1)
BA92 (GCA_000585055.1)
HKU1 (GCA_000987445.1)
CANDO2 (GCA_009467885.1)
Camejo (GCA_003332265.1)
UBA6658 (GCA_002455435.1)
BA93 (GCA_000585075.1)
CANDO1 (GCA_009467855.1)
Ca. Accumulibacter aalborgensis (GCA_900089955.1)
UBA704 (GCA_002304785.1)
