# Supplementary material for: Recovery of complete genomes and non-chromosomal replicons from activated sludge enrichment microbial communities with long read metagenome sequencing
Source: NPJ Biofilms Microbiomes. 2021 Mar 16;7:23. doi: 10.1038/s41522-021-00196-6 (PMC7966762; doi:10.1038/s41522-021-00196-6)
Supplement: Supplementary file 12 — Reporting Summary [file 41522_2021_196_MOESM12_ESM.pdf]

nature research

Corresponding author(s): Rohan Benjamin Hugh WILLIAMS

Last updated by author(s): Dec 16, 2020

Reporting Summary

Nature Research wishes to improve the reproducibility of the work that we publish. This form provides structure for consistency and transparency in reporting. For further information on Nature Research policies, see our Editorial Policies and the Editorial Policy Checklist.

Statistics

For all statistical analyses, confirm that the following items are present in the figure legend, table legend, main text, or Methods section.

n/a	Confirmed	
☐	☒	The exact sample size (*n*) for each experimental group/condition, given as a discrete number and unit of measurement
☐	☒	A statement on whether measurements were taken from distinct samples or whether the same sample was measured repeatedly
☒	☐	The statistical test(s) used AND whether they are one- or two-sided<br>*Only common tests should be described solely by name; describe more complex techniques in the Methods section.*
☒	☐	A description of all covariates tested
☒	☐	A description of any assumptions or corrections, such as tests of normality and adjustment for multiple comparisons
☐	☒	A full description of the statistical parameters including central tendency (e.g. means) or other basic estimates (e.g. regression coefficient) AND variation (e.g. standard deviation) or associated estimates of uncertainty (e.g. confidence intervals)
☒	☐	For null hypothesis testing, the test statistic (e.g. *F*, *t*, *r*) with confidence intervals, effect sizes, degrees of freedom and *P* value noted<br>*Give P values as exact values whenever suitable.*
☒	☐	For Bayesian analysis, information on the choice of priors and Markov chain Monte Carlo settings
☒	☐	For hierarchical and complex designs, identification of the appropriate level for tests and full reporting of outcomes
☒	☐	Estimates of effect sizes (e.g. Cohen's *d*, Pearson's *r*), indicating how they were calculated

Our web collection on statistics for biologists contains articles on many of the points above.

Software and code

Policy information about availability of computer code

Data collection

Data were obtained using off-the-shelf software running on Oxford Nanopore Technologies MinION and Illumina MiSeq and HiSeq2500 sequencing platforms.

Data analysis

Analysis of long read data
Basecalling: guppy version 1.8 or 1.9 (see manusucript)
Adaptor trimming: Porechop version 0.0.2
Assembly: Canu v1.8 or v1.9 (see manuscript), Unicycler v0.4.7 or version 0.4.8 (see manuscript); Flye version 2.4.
Annotation long read assembled contigs: DIAMOND version 0.9.24, MEGAN Community Edition version 6.17.0
Genome quality and annotation: CheckM version 1.0.11, Prokka version 1.13, dRep version 2.2.3, GTDB-Tk version 0.3.2, minimap version 2.17, bedtools::genomeCoverageBed version 2.26.0, sina-1.6.0-linux.

Analysis of short read data
Read QC/filtering: cutadapt version 1.14
Metagenome assembly: SPAdes version 3.12.0-Linux or version 3.14.0-Linux; see manuscript)
Genome binning: MetaBAT2
Genome quality and annotation: CheckM version 1.0.11, GTDB-Tk version 0.3.2.

Comparative analysis of short and long read assemblies
BLASTN version 2.7.1+, custom R package named srac2lrac (available at https://github.com/rbhwilliams/srac2lrac (see manuscript).

Long read error correction
Medaka version 0.11.5, Racon version 1.4.3.

Genome refinement
IGV version 2.4.14, BCFtools version 1.9

Detection of non-chromosomal replicons
CheckV version 0.6.0

Hybrid metagenome assembly, and related analysis
SPAdes version 3.14.1-Linux, OPERA-MSversion 0.9.0, minimap version 2.17, CheckM version 1.1.3, GTDB-Tk version 1.3.0, bedtools genomeCoverageBed version 2.26.0

General data handling, analysis and data visualisation
R statistical computing environmental version 3.6.1.

For manuscripts utilizing custom algorithms or software that are central to the research but not yet described in published literature, software must be made available to editors and reviewers. We strongly encourage code deposition in a community repository (e.g. GitHub). See the Nature Research guidelines for submitting code & software for further information.

Data

Policy information about availability of data

All manuscripts must include a data availability statement. This statement should provide the following information, where applicable:

- Accession codes, unique identifiers, or web links for publicly available datasets
- A list of figures that have associated raw data
- A description of any restrictions on data availability

Raw sequence data from long and short read sequencing is available at NCBI via the following BioProject accessions: PAO1: PRJNA509764; PAO2: PRJNA611629; PAO3A and PAO3B}: PRJNA606905 and PAO4: PRJNA607349. The chromosomal sequences are available at NCBI via the Genbank accession numbers listed in Table 1. Data products from this study are contained in two Zenodo submissions (one made before initial submission, the second following review): the first submission (https://doi.org/10.5281/zenodo.3695987) includes: 1) LRAC sequence from each dataset; 2) whole genome sequence (FASTA), genome quality statistics (CheckM) and genome annotation data (Prokka) from 21/22 genomes listed in Table 1 for each of the five correction procedures; 3) short read assembled sequence and binning results; 4) concordance statistic data and results; 5) short and long read per--base coverage data for 21/22 genomes and 5) two manually corrected genomes of Candidatus Accumulibacter along with detailed notes explaining the procedures that were applied. The second Zenodo submission (https://doi.org/10.5281/zenodo.4317309) contains equivalent data for the Contedobacter genome and outputs from both hybrid metagenome workflows. The R code for performing the concordance statistic analysis are available at https://github.com/rbhwilliams/srac2lrac including test data and scripts taken from the PAO2 data.

Field-specific reporting

Please select the one below that is the best fit for your research. If you are not sure, read the appropriate sections before making your selection.

☐ Life sciences ☐ Behavioural & social sciences ☒ Ecological, evolutionary & environmental sciences

For a reference copy of the document with all sections, see nature.com/documents/nr-reporting-summary-flat.pdf

Ecological, evolutionary & environmental sciences study design

All studies must disclose on these points even when the disclosure is negative.

Study description	Sampling of four continuous-culture enrichment bioreactors for the purposes of evaluating the technical feasibility of performing long read metagenome assembly.
Research sample	We sampled a set of four continuous-culture enrichment bioreactors, established and operated by us. Each reactor had been inoculated with activated sludge from full-scale wastewater treatment plants in Singapore, and operated with under different conditions to try and enrich the microbial community biomass for specific species of polyphosphate accumulating organisms (PAO). Samples were obtained from each of these bioreactors to establish the technical feasibility of recovering genomes of the member species of these communities using long read sequencing.
Sampling strategy	Each bioreactor was sampled on a single occasion for the purpose of obtaining DNA for purposes of genome recovery.
Data collection	For the bioreactors described as PAO1, PAO2 and PAO4 2mL of suspended biomass was obtained from each bioreactor via in-built sampling ports and DNA extraction procedures commenced immediately following sampling. The samples were collected by author Bessarab together with authors Roy and Qiu. For the bioreactors described as PAO3A and PAO3B, samples had been snap frozen at -80 degrees Celsius by author Zuniga-Montanez and provided to author Bessarab. These samples were then thawed on ice prior to commencement of DNA extraction.
Timing and spatial scale	Each bioreactor was sampled on a single occasion for the purpose of obtaining representative DNA for purposes of genome recovery.
Data exclusions	No data exclusion: all collected samples were used for analysis.

Reproducibility	Not applicable as the stated purpose of the study is to establish the technical feasibility of recovering member genomes from individual microbial communities.
Randomization	Not applicable as no experimental treatments were delivered.
Blinding	Not applicable given the stated study design is a technical evaluation of genome recoverability.

Did the study involve field work? ☐ Yes ☒ No

Reporting for specific materials, systems and methods

We require information from authors about some types of materials, experimental systems and methods used in many studies. Here, indicate whether each material, system or method listed is relevant to your study. If you are not sure if a list item applies to your research, read the appropriate section before selecting a response.

Materials & experimental systems

n/a	Involved in the study
☒	☐ Antibodies
☒	☐ Eukaryotic cell lines
☒	☐ Palaeontology and archaeology
☒	☐ Animals and other organisms
☒	☐ Human research participants
☒	☐ Clinical data
☒	☐ Dual use research of concern

Methods

n/a	Involved in the study
☒	☐ ChIP-seq
☒	☐ Flow cytometry
☒	☐ MRI-based neuroimaging
